# Provenance, modification and use of manganese-rich rocks at Le Moustier (Dordogne, France)

**DOI:** 10.1371/journal.pone.0218568

**Published:** 2019-07-17

**Authors:** Africa Pitarch Martí, Francesco d’Errico, Alain Turq, Eric Lebraud, Emmanuel Discamps, Brad Gravina

**Affiliations:** 1 UMR 5199 CNRS, De la Préhistoire à l’Actuel: Culture, Environnement, et Anthropologie (PACEA), Université de Bordeaux, Pessac, France; 2 Seminari d'Estudis i Recerques Prehistòriques (SERP), Facultat de Geografia i Història, Departament d'Història i Arqueologia, Universitat de Barcelona, Montalegre, Barcelona, Spain; 3 SSF Centre for Early Sapiens Behavior (SapienCe), University of Bergen, Bergen, Norway; 4 Musée de Préhistoire, Sauveterre-la-Lémance, Lot-et-Garonne, France; 5 UMR 5026 CNRS, Institut de Chimie de la Matière Condensée de Bordeaux (ICMCB), Université de Bordeaux, Pessac, France; 6 UMR 5608 CNRS, Travaux et Recherches Archéologiques sur les Espaces, les Cultures et les Sociétés (TRACES), Université Toulouse Jean Jaurès Maison de la Recherche, Toulouse, France; Universita degli Studi di Milano, ITALY

## Abstract

The use of colouring materials by Neanderthals has attracted a great deal of attention in recent years. Here we present a taphonomic, technological, chemical-mineralogical and functional analysis of fifty-four manganese rich lumps recovered during past and on-going excavations at the lower rockshelter of Le Moustier (Dordogne, France). We compare compositional data for archaeological specimens with the same information for twelve potential geological sources. Morphometric analysis shows that material from Peyrony’s excavations before the First World War provides a highly biased picture of the importance of these materials for Mousterian groups. These early excavations almost exclusively recovered large modified pieces, while Mn-rich lumps from the on-going excavations predominantly consist of small pieces, only half of which bear traces of modification. We estimate that at least 168 pieces were not recovered during early work at the site. Neanderthals developed a dedicated technology for processing Mn-rich fragments, which involved a variety of tools and motions. Processing techniques were adapted to the size and density of the raw material, and evidence exists for the successive or alternating use of different techniques. Morphological, textural and chemical differences between geological and archaeological samples suggest that Neanderthals did not collect Mn-rich lumps at the outcrops we sampled. The association and variability in Mn, Ni, As, Ba content, compared to that observed at the sampled outcrops, suggests that either the Le Moustier lumps come from a unique source with a broad variation in composition, associating Mn, Ni, As, Ba, or that they were collected at different sources, characterized either by Mn-Ni-As or Mn-Ba. In the latter case, changes in raw material composition across the stratigraphy support the idea that Neanderthal populations bearing different stone tool technologies collected Mn fragments from different outcrops. Our results favour a use of these materials for multiple utilitarian and symbolic purposes.

## 1. Introduction

The material culture of Neanderthal and pre-Neanderthal populations living in Europe during the Middle and Upper Pleistocene is traditionally perceived as almost exclusively consisting of stone tools produced with different knapping and retouching techniques [1–5]. While wood-based technologies [6], colouring materials [7–8], abstract engravings [9] and burial practices [10–12] were demonstrated some time ago to be part of the Neanderthal behavioural repertoire, this evidence was considered too anecdotal to draw definitive conclusions on the complexity and variability of both the technical systems of these populations and their cognitive/symbolic capacities. In addition, modifications present on several objects, previously interpreted as engraved or worn as ornaments, were shown to be the consequence of natural phenomena [13]. Over the past decade, several new discoveries and reanalyses of old archaeological collections highlighted new cultural innovations and demonstrated the systematic nature of previously recorded ones [14–18]. It is now widely accepted that European Neanderthal populations hunted large mammals and exploited marine and plant resources, potentially even for medicinal purposes [19–22]. These groups also worked wood to fashion tools and hunting weapons [23–25], transformed bone into tools to process hides [26] or retouch stone tools [27–30], mastered fire for warmth and cooking [31–32], used pyrotechnology to render pitch from birch sap for tool hafting [33–34], and were able to adapt their clothing to different climates [35].

Multiple lines of evidence—the collection of crystals, fossils and marine shells [36–41], colouring of objects [36, 42–44], burial practices [45–49], engravings on objects and caves walls [50], possible mathematical notations [51], extraction of bird feathers and talons probably for body decorations [15–16, 52–58], the construction of a circular structure from intentionally broken stalagmites [59], and pigment use [60]—including potential abstract depictions on cave walls ([61–63] but see [64–66])—clearly show Neanderthal cultures to equally include symbolic dimension. Attested by a growing body of palaeogenetic evidence, repeated interbreeding between Neanderthals, Denisovans and modern humans left detectable traces in the modern human genome [67–69], phenotype [70] and immune system [71], demonstrating these human populations recognised each other as desirable companions and considered each other and their respective cultures as fundamentally human. However, aspects of Neanderthal cultures other than stone tool production systems are still poorly documented and understood.

The use of colouring materials–primarily iron and manganese oxy-hydroxides–is probably the element of Neanderthal cultural adaptation other than lithic technology that has attracted the most attention in recent years. These efforts have led to the publication of new discoveries [36, 44, 72–74] following the reappraisals of old collections [40, 74–76], attempts to identify the geological sources of colouring materials used by Neanderthals [74, 77–80], the documentation of traces of modification and use [40, 74–75, 80–82], as well as multiple hypotheses concerning the potential functions of these materials for Neanderthal societies, including camouflage [83–86], body painting, decorating skins and objects [40, 85], igniting fires [32, 87] and painting cave walls [36]. However, attempts to succinctly document the provenance, selection, processing and use of colouring materials at major stratified Mousterian sites remain rare.

Here we present a taphonomical, technological, compositional and functional analysis of unpublished Mn-rich lumps recovered during previous and on-going excavations at the lower rockshelter of Le Moustier in the Périgord region of southwestern France. The interest of this study, which presents the first systematic XRF analysis of Mn-rich fragments from a major Mousterian site, is threefold. First, as the various previous excavations at Le Moustier all used different methodologies and hence different recovery rates, the analysis of Mn-rich fragments from on-going excavations using modern methods allows us to assess potential biases in size and quantity of manganese lumps in the older collections. This has implications for the interpretation of Mn-rich objects from numerous sites investigated long ago with similar excavation methods and is therefore key for evaluating the importance of manganese oxy-hydroxides for Neanderthal societies. Second, our analysis both builds upon a recently published PIXE characterisation of Mn-rich sources from the region [78] and integrates four additional potential pigment sources. Third, it represents the first attempt to contrast the elemental and structural composition of Mn-rich pieces from a major site with their treatment and stratigraphic/cultural attribution with the aim of exploring behavioural consistencies and patterns of diachronic change in this fundamental aspect of Neanderthal cultural adaptations.

## 2. Materials and methods

### 2.1. Archaeological context

**Fig 1:**
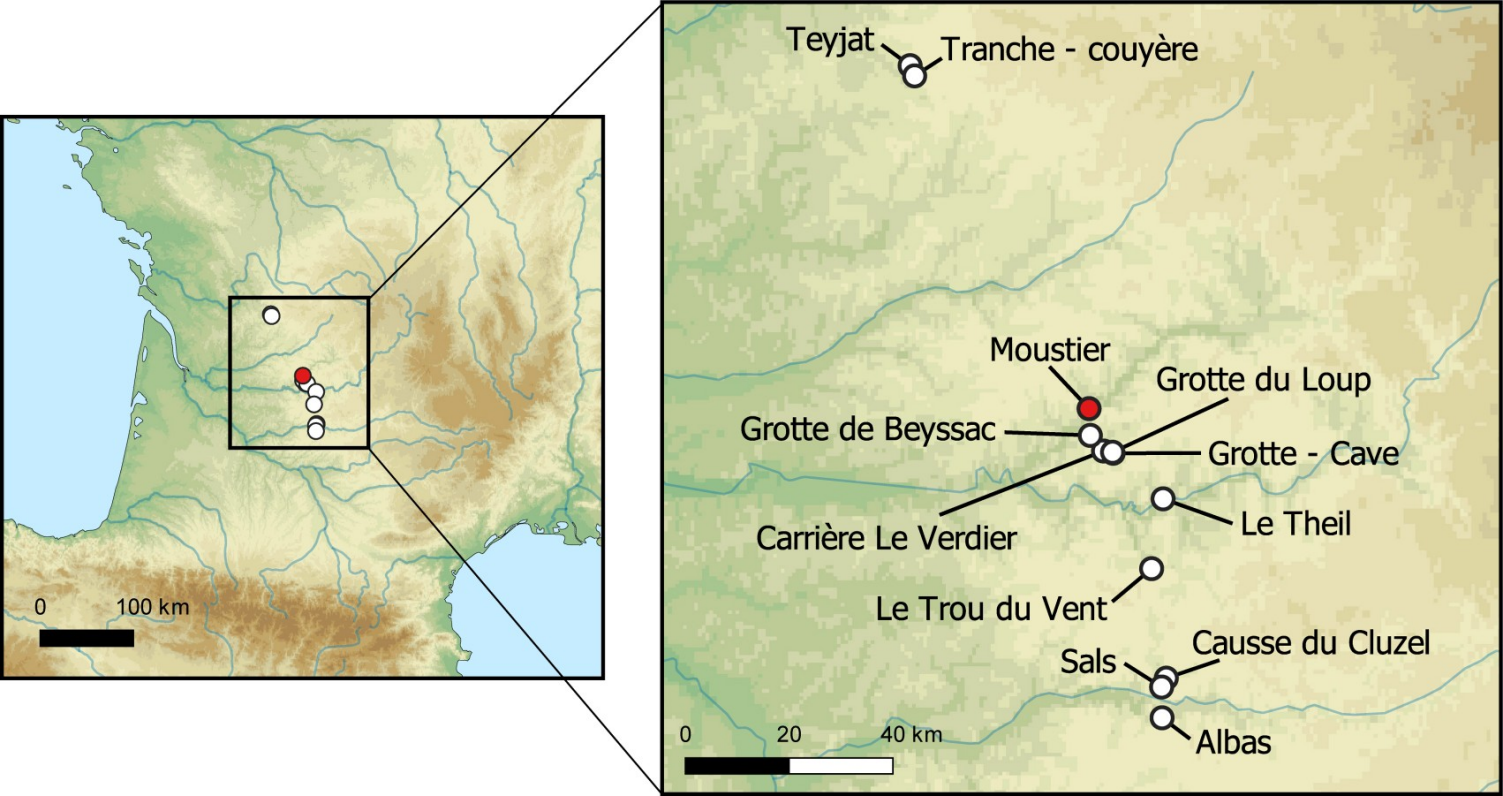
Location of Le Moustier and potential geological sources analysed in this study. Red triangle: Le Moustier; white circles: Mn-rich outcrops. Background map generated using GEBCO https://www.gebco.net and Natural Earth https://www.naturalearthdata.com datasets.

The site of Le Moustier (lat. 44.994243, long. 1.059741) is located in Saint-Léon-sur-Vézère, Dordogne, France, on the right bank of the Vézère River (Fig 1). It comprises three superimposed rockshelters: the *Trou de Bréchou* and the Upper and Lower rockshelters. Our work concentrates on the Lower Shelter of site (hereafter referred to as Le Moustier), which was first excavated by Otto Hauser and then by Denis Peyrony [87], who identified several Mousterian layers in the approximately 4 m thick archaeological sequence. Limited fieldwork by Laville and Rigaud [88] in the 1960s and a test pit by Geneste and Chadelle [89] in the 1980s provided more precise information concerning the geology, sedimentology, and chronology of the site. A summary of Le Moustier’s long excavation history has been published elsewhere [90]. The site is currently being re-excavated by two of us (BG, ED; Permit n° ALPC-AQ-2016-066 issued by the Préfet de la region Aquitaine–Limousin–Poitou-Charentes) in the larger framework of a multi-disciplinary project aimed at reanalysing and re-excavating a number of key Mousterian sequences in south-western France. Recent re-assessments of lithic and faunal assemblages from previous excavations at Le Moustier [90–94] produced a new interpretation of the site’s archeo-stratigraphic sequence, including the reattribution of several layers to particular lithic techno-complexes (LTC) (see [95] for details concerning the definition of LTC from south-western France) and the identification of previously undetected shifts in subsistence strategies. This new vision of the Le Moustier sequence was the impetus behind restarting excavations at the site in 2014.

Of specific interest to this study, revision of material recovered by Geneste and Chadelle led to Peyrony’s layer H being reassigned from Bordes’ “MTA-B” facies to the Discoid LTC. The same revision divided layer G into a lower (G1/G2) Levallois occupation overlain by one focused almost entirely on bifacial shaping (G3/G4). While new excavations are yet to reach layer G, the recently excavated lithic assemblage from stratigraphic units equivalent to the upper part of Peyrony’s layer H confirm this reattribution [95]. Finally, new Mn-rich rocks recovered from stratigraphic units correlatable with the base of Peyrony’s layer K are associated uniquely with Middle Palaeolithic artefacts. Of notable importance is the fact that the material recovered by Peyrony from his “Layer H”, which also produced most of the manganese lumps, was demonstrated to reflect several distinct occupation phases [90].

### 2.2. Archaeological collections

We identified eighteen black manganese lumps amongst material from Peyrony’s excavations [87, 96], two from Laville and Rigaud’s [89], and two from Geneste and Chadelle’s [89], all of which are housed at the *Musée National de la Préhistoire (MNP)*, Les Eyzies-de-Tayac, France. To this can be added thirty-two fragments recovered during on-going fieldwork (Table 1, Figure A in S1 Fig). The analysed sample therefore comprises 54 lumps of Mn-rich rocks. While only the layer attribution is known for the fragments recovered by Peyrony, those collected by Laville and Rigaud (L&R) and Geneste and Chadelle (G&Ch) can be attributed to sub-levels identified on the basis of sedimentological criteria [88]. Black manganese lumps recovered during the current field project come from stratigraphic units correlatable with both Peyrony’s stratigraphy and previously identified sub-levels. In addition to 3D piece-plotted material, manganese mineral fragments collected during the systematic sorting of the wet-sieve residues (4 and 2 mm meshes) can equally be attributed to a stratigraphic unit with the same precision. The correlation of new stratigraphic units identified in the field with those identified by Peyrony and Laville and Rigaud [97] therefore provide a stratigraphically secure sample for exploring Neanderthal pigment use at Le Moustier. After a preliminary analysis conducted at the MNP, we received permission to study selected pieces at the PACEA laboratory of the University of Bordeaux.

**Table 1:** Contextual information and analyses conducted on the Mn-rich black lumps from Le Moustier.

Piece	Excavation	Year	Archaeological context						Analyses					S3 Fig
			Layer (#)	Spit	US	Zone	Square	Quadrant	Optical microsc.	EDXRF	SEM-EDS	μ-RS	XRD	
MOU-MNP-01	P	1912–1914	B						x	x				
MOU-MNP-02	P	1912–1914	B						x	x				
MOU-MNP-03	P	1912–1914	B						x	x		x		A
MOU-MNP-04	P	1912–1914	B						x	x	x	x	x	B
MOU-MNP-05	P	1912–1914	B						x	x		x		C
MOU-MNP-06	P	1912–1914	F						x	x	x	x		D
MOU-MNP-07	P	1912–1914	J						x	x				
MOU-MNP-08	P	1912–1914	H						x	x				
MOU-MNP-09	P	1912–1914	H						x	x		x		E
MOU-MNP-10	P	1912–1914	H						x	x				
MOU-MNP-11	P	1912–1914	H						x	x		x		F
MOU-MNP-12	P	1912–1914	H						x	x		x	x	G
MOU-MNP-13	P	1912–1914	H						x	x				
MOU-MNP-14	P	1912–1914	H						x	x				
MOU-MNP-15	P	1912–1914	H						x	x				
MOU-MNP-16	P	1912–1914	H						x	x				
MOU-MNP-17	P	1912–1914	H						x	x				
MOU-MNP-18	P	1912–1914	H						x	x				
MOU-MNP-19	G & Ch	1982	G						x	x		x	x	H
MOU-MNP-20	G & Ch	1985	G						x	x		x	x	I
MOU-MNP-L&R-H7	L & R	1969	H (*)						x	x		x	x	J
MOU-MNP-L&R-H8	L & R	1969	H (*)						x	x		x	x	K
MOU-G&D-5983	G & D	2015	H (*)	7	B2	B	C50	a	x	x		x		L
MOU-G&D-6846-a	G & D	2015	H (*)	8	B3	B	C49	c	x	x		x		M
MOU-G&D-6846-b	G & D	2015	H (*)	8	B3	B	C49	c	x					
MOU-G&D-6846-c	G & D	2015	H (*)	8	B3	B	C49	c	x					
MOU-G&D-6857	G & D	2015	H (*)	8	B3	B	C49	a	x	x	x	x	x	N
MOU-G&D-7527-a	G & D	2015	H (*)	9	B3	B	C49	a	x	x		x		O
MOU-G&D-7527-b	G & D	2015	H (*)	9	B3	B	C49	a	x	x				
MOU-G&D-4584-a1	G & D	2015	K	14	A3	A	F47	-	x	x				
MOU-G&D-4584-a2	G & D	2015	K	14	A3	A	F47	-	x					
MOU-G&D-4584-a3	G & D	2015	K	14	A3	A	F47	-	x					
MOU-G&D-4584-a4	G & D	2015	K	14	A3	A	F47	-	x					
MOU-G&D-4584-a5	G & D	2015	K	14	A3	A	F47	-	x					
MOU-G&D-4584-a6	G & D	2015	K	14	A3	A	F47	-	x					
MOU-G&D-5255-a1	G & D	2015	K	15	A3	A	F47	-	x	x				
MOU-G&D-5255-a2	G & D	2015	K	15	A3	A	F47	-	x					
MOU-G&D-5902-a2	G & D	2015	K	16	A3	A	F47	-	x	x				
MOU-G&D-5902-a3	G & D	2015	K	16	A3	A	F47	-	x					
MOU-G&D-5902-a4	G & D	2015	K	16	A3	A	F47	-	x					
MOU-G&D-5902-a5	G & D	2015	K	16	A3	A	F47	-	x					
MOU-G&D-9796	G & D	2016	H (*)	15	B3	B	C49	a	x	x		x	x	P
MOU-G&D-11571	G & D	2016	H (*)	18	B3	B	C49	a	x	x		x	x	Q
MOU-G&D-8975	G & D	2016	H (*)	13	B3	B	C49	a	x	x				
MOU-G&D-9858-a	G & D	2016	H (*)	15	B3	B	C49	a	x	x				
MOU-G&D-9858-b	G & D	2016	H (*)	15	B3	B	C49	a	x	x				
MOU-G&D-9858-c	G & D	2016	H (*)	15	B3	B	C49	a	x					
MOU-G&D-9858-d	G & D	2016	H (*)	15	B3	B	C49	a	x					
MOU-G&D-11869	G & D	2016	H (*)	18	B3	B	C50	a	x	x				
MOU-G&D-11236-a	G & D	2016	H (*)	17	B3	B	C49	c	x	x				
MOU-G&D-11236-b	G & D	2016	H (*)	17	B3	B	C49	c	x					
MOU-G&D-12575-a	G & D	2016	H (*)	19	B3	B	C49	a	x	x				
MOU-G&D-12575-b	G & D	2016	H (*)	19	B3	B	C49	a	x					
MOU-G&D-13440	G & D	2016	H (*)	*recti-coupe*	B3	B	B50	d	x					

(#) According to Peyrony's stratigraphy (Peyrony, 1930)

(*) Recently excavated stratigraphic units that can be correlated with the summit of Peyrony's layer H. Excavation code—P: Peyrony; G & Ch: Geneste and Chadelle; L & R: Laville and Rigaud; G & D: Gravina and Discamps.

### 2.3. Geological samples

Data collated from the literature [98–100], the French Geological Survey (BRGM—*Bureau de Recherches Géologiques et Minières*) and historical archives (*Archives Départementales de la Dordogne*) helped identify Mn-rich formations in the Dordogne and Lot departements of southwestern France. Three areas were surveyed—the Nontronais, the area between the Vézère and the Dordogne Rivers, and the area between the Dordogne and Lot Rivers–for a total of twenty-two outcrops potentially containing Mn-rich materials. However, we were only able to field-check, georeference, sample and analyse twelve outcrops, which is nevertheless four more than in a previous study [78]) (Fig 1, Table 2). In order to evaluate variation in mineral composition within a given outcrop, several samples were collected, when possible, from each outcrop and special attention was paid in choosing samples representative of the morphological and textural variability of the outcrop. No specific permits were required for this study, and no endangered or protected species were involved.

**Table 2:** Information on Mn-rich outcrops analysed in this study.

Outcr code	Outcr name	Village	Reg	Geographic coordinates		BRGM map	Host formation	Dep. App.	Mine Quar.	N	Analyses				
				Lat	Long						OM	ICP-MS	ED-XRF	SEM-EDS	XRD
TEY	Teyjat	Teyjat	Do	45.5808328	0.596588	710	Quaternary colluvium	Pat.		1	x		x		x
TRA	Tranche -couyère	Saint Martin le Pin	Do	45.563271	0.608526	710	Tertiary sand and clay deposits alternating a Jurassic limestone	Nod.		1	x		x		x
BEY	Carrière Le Verdier	Les Eyzies de Tayac Sireuil	Do	44.92127	1.097517	807	Quaternary rock debris deposits	Nod.	x	1	x	x	x	x	x
VER	Grotte de Beyssac	Les Eyzies de Tayac Sireuil	Do	44.947805	1.064429	808	Cretacic calcarenite	Lens		6	x	x	x	x	x
CAV	Grotte—Cave	Saint André d’Allas	Do	44.917836	1.121022	808	Cretacic calcarenite	Crust		2	x		x	x	x
LOU	Grotte du Loup	Saint André d’Allas	Do	44.919497	1.11978	808	Cretacic calcarenite	Crust		4	x	x	x	x	x
SAR	Sarlat	Sarlat la Canéda	Do	NA	NA	808	Cretacic calcarenite	Lens		1	x		x	x	x
THE	Le Theil	Vitrac	Do	44.840691	1.24573	808	Cretacic limestone	Var.	x	3	x	x	x	x	x
BOU	Le Trou du Vent	Bouzic	Do	44.71912	1.221456	832	Jurassic limestone	Var.		4	x		x	x	x
CAU	Causse du Cluzel	Pontcirq	Lo	44.529568	1.263074	856	Jurassic limestone	Tab.	x	3	x	x	x	x	x
SAL	Sals	Labastide du Vert	Lo	44.514082	1.252215	856	Jurassic limestone	Tab.	x	3	x		x	x	x
ALB	Albas	Albas	Lo	44.460711	1.254982	856	Jurassic limestone	Var.	x	3	x		x	x	x

Outcr.: outcrop; Reg.: region; Lat.: latitude; Long.: longitud; BRGM: *Bureau de Recherches Géologiques et Minières*geological map. Scale: 1/50000; Dep. App.: deposit appearance; Pat.: patina; Nod.: nodule; Var.: variable; Tab.: tabular; Quar.: quarry; N: number of analysed samples; Do: Dordogne; Lo: Lot. NA: Not available.

### 2.4. Microscopic analysis

The archaeological lumps were examined and photographed with a motorised Leica Z6 APOA microscope equipped with a DFC420 digital camera in order to visually characterize raw materials and document traces of anthropogenic modification. In some cases, uploaded images were treated with the Leica Application Suite (LAS) equipped with the Multifocus module, and Leica Map DCM 3D computer software.

Variables recorded on archaeological Mn-rich lumps included length, width, thickness, weight, density (very low, low, medium, high, very high), hardness (soft, medium, hard), type of lump fragment (nodule, crust), morphology, hue, appearance, and rock structure (columnar, granular, laminated, massive, porous). Density, hue, and hardness were evaluated by visual inspection and stains produced while handling the objects. The type (grinding, notching, scraping, percussion) and intensity of anthropogenic modification (low, medium, intense, very intense), number of facets produced by grinding, facet morphology (flat, convex, concave), presence of striations on facets, and the striation orientation (parallel, oblique, perpendicular, two directions) were also recorded.

### 2.5. Geochemical characterization

Elemental analysis of the archaeological lumps was performed using a hand-held Ametek SPECTRO xSORT energy dispersive X-ray fluorescence (EDXRF) spectrometer equipped with a silicon drift detector (SDD), a low power W X-ray tube with an excitation source of 40 kV, and an X-ray beam of 8 mm. Spectra acquisition times were set to 60 s. Measurements were carried out with a constant working distance by using a positioning device consisting of a lead receptacle to which the spectrometer is fixed. Two to five measurements were taken on different flatter and cleaner areas of each archaeological piece. Element contents were calculated as the average of these acquisitions. The spectrometer is internally calibrated by an automated measure of the elemental content of a standard metal shutter. However, in order to more precisely quantify the elemental composition of the archaeological samples, which present an extremely variable Mn content, a dedicated calibration was developed. Based on Lucas-Tooth & Price’s method [101], our calibration uses five certified reference materials (CRM) with variable manganese oxide content and seven self-produced standards previously characterised by ICP-AES and ICP-MS at the *Service d’Analyse des Roches et Minéraux* (SARM, Nancy, France). Five of the self-produced standards (STD-01, STD-02, STD-03, STD-06, STD-07) were prepared with geological samples from Mn outcrops in the Dordogne and Lot regions, and two of them (STD-04, STD-05) with archaeological pieces found out of context (from the backdirt of Bordes’ excavations at Pech-de-l’Azé I). The corrections used for the calibrations are given in Table A in S1 Table. Calibration slopes were adjusted for twelve major, minor, and trace elements. The R^2^ of the final calibration curves is systematically higher than 0.98 (Figure B in S1 Fig). Results before and after calibration are provided in Tables B and C in S1 Table.

Selected archaeological and geological samples (see Tables 1 and 2), representative of the textural and elemental variability of both assemblages as identified by microscopic inspection and EDXRF, were studied with scanning electron microscopy coupled with energy dispersive X-ray spectroscopy (SEM-EDS), micro-Raman spectroscopy (μ-RS) and X-ray diffraction (XRD).

Elemental composition, morphology and distribution of minerals were studied with two SEM-EDS instruments. For geological samples, we used a FEI Quanta 200. The observations and analyses were conducted under a low vacuum mode with an accelerating voltage of 15 kV. Backscattered electron (BSE) images were collected with a SiLi detector, EDS analyses were carried out with a SDD-EDAX detector. Similar magnifications were used for the EDS analyses of each sample, and the working distance was kept constant (10 mm). Acquisition time was set to 100 s for each EDS spectrum. For archaeological specimens, we used a JEOL 6460 LV SEM coupled to a SDD semi-conductor (Oxford INCA 30 spectrometer). Backscattered electron images (BSE) and elemental analyses were also obtained under a low vacuum mode—allowing imaging and analyses without any specific preparation of samples- with an accelerating voltage of 20 kV. The other analytical conditions were the same used for the geological samples. The mineralogical composition of crystals and grains in modified and unmodified Mn-rich lumps was determined by μ-RS using a SENTERRA Dispersive Raman Microscope (Bruker) equipped with an internal calibration system. The working area was examined using an integrated colour camera. Measurements were acquired with a 532 nm laser and a power of 0.2 mW in order to avoid thermal transformation of mineral phases. The spectra were recorded with an integration time varying from 5 to 10 s, in a spectral range from 100 to 2200 cm^-1^, and with a number of co-additions varying between 5 to 10 depending on the presence of fluorescence radiation and signal-to-noise ratio. Data were collected and treated with the software package OPUS 7.2. Mineral identification was based on the comparison of the recorded spectra with those of available spectra libraries [102–105]. For an overall assessment of the mineral phases present in the geological samples and the archaeological pieces, XRD was also performed by using two diffractometers: in the first case we used a PANalytical X’pert MPD-PRO diffractometer (Bragg Brentano Theta-Theta geometry), with a Cu Kα anticathode (λ = 1.5418 Å). The working tension and intensity were set at 45 kV and 40 mA, respectively, and the time of analysis was between 3 and 4 h, depending on the sample. Samples were previously ground and homogenized with an agate mortar. In the second case, data was collected with a Bruker D8 Advance diffractometer, equipped with a PSD Lynxeye detector and operating with a Cu Kα radiation (λ = 1.5405 Å). A Bragg-Brentano geometry was used on the surface of the archaeological pieces. In order to limit the divergence of the incident rays, a divergent slit of 0.2 mm was applied. Mineralogical phases were in both cases identified by using the routine DIFFRAC.SUITE EVA software package (Bruker AXS GmbH, Germany), combined with the specific powder diffraction file (PDF2) database (International Centre for Diffraction Data—ICDD, Pennsylvania, USA).

### 2.6. Statistical analysis

Prior to statistical analysis, raw concentration data was subjected to two mathematical treatments: replacement of values below the detection limit [106] and centred log ratio transformation–clr- [107]. All data analyses were performed with the *R* software suite and the *ade4* package [108]. We performed principal component analysis (PCA) of EDXRF concentrations for the twelve major, minor, and trace elements most frequently detected in the black lumps (Si, K, Ca, Ti, Mn, Fe, Ni, Zn, As, Sr, Pb, and Ba). We also used ternary plots combining elements identified by the PCA as the more discriminant to explore correlations between variables and patterning in types of Mn-rich compounds. The archaeological lumps were analysed as groups taking into account their stratigraphic origin, and by outcrop for the geological samples.

## 3. Results

### 3.1. Archaeological samples

#### 3.1.1 Collection description

Table D in S1 Table summarizes dimensional, morphological and textural information as well as the occurrence of anthropogenic modifications on the archaeological Mn-rich lumps. The fifty-four manganese pieces from Le Moustier comprise fragments of crusts and nodules with lengths ranging from 6.8 to 58 mm, and weights ranging from 0.06 and 110 g. They feature different densities: half (n = 28; 51.8%) present a low density, followed by a smaller proportion displaying either a very low (n = 6; 11.1%), or a medium (n = 12; 22.2%), and a high density (n = 6; 11.1%). Only two pieces (3.7%) are very dense. The majority of fragments (n = 47; 87%) are relatively hard, two pieces (3.7%) have an intermediate hardness, and four pieces (7.2%) are soft. Most are broken and the small proportion of either unbroken pieces or for which the original morphology, either natural or modified, could be established (n = 12; 22.2%) show, in decreasing order, are pyramidal, prismatic, cubic or ellipsoidal in shape. The pieces show a variety of hues (black, brownish black, very dark grey, dark grey, anthracite), with very dark grey as prevailing colour (n = 39; 72%). Most of them (n = 41; 76%) display an irregular dull surface, some show a metallic sheen, bluish reflections or a glossy appearance in certain places. A small proportion (n = 9; 16.6%) bears botryoidal or ribbed surfaces. Most have a massive structure (n = 51; 94.4%), and three specimens granular, columnar or laminated structure. Visible pores are evident on twenty-five specimens (46.3%). Half of the fragments (n = 28; 51.8%) show clear traces of modification.

**Fig 2:**
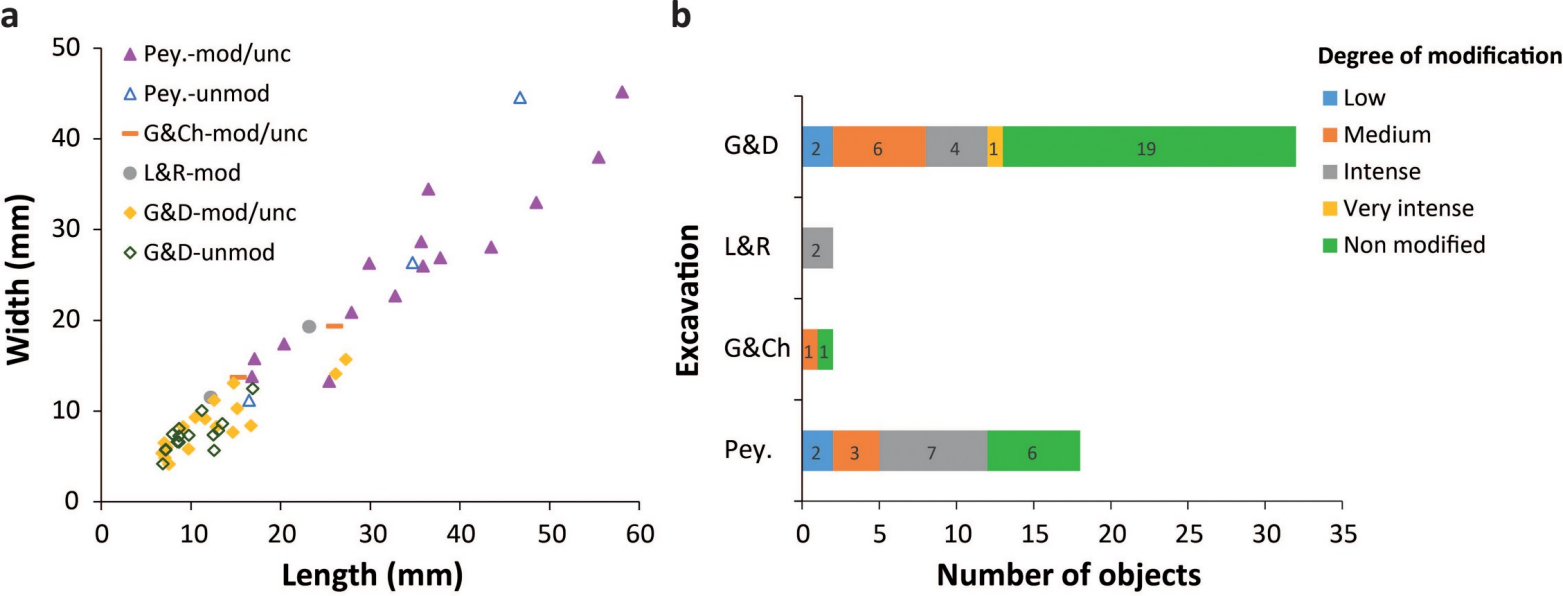
Taphonomic analysis of the manganese-rich rocks. Difference in size (a) and degree of modification (b) of manganese lumps from Le Moustier excavations.

A clear difference in size and occurrence of anthropogenic modifications is evident amongst black manganese lumps recovered during the four Le Moustier excavations. Peyrony’s sample is almost exclusively composed of large pieces, ranging from 20 to 60 mm, most of which bear traces of modifications. In contrast, the G&D sample (on-going excavations) is almost exclusively composed of pieces smaller than 20 mm in length, with only half bearing traces of modification. The few pieces from L&R and G&Ch’s excavations fall in between the size ranges of the first two collections and are all modified (Fig 2A). Comparison of the degree of modification between collections reveals that intensively modified objects are overrepresented in the Peyrony’s sample compared to the G&D (Fig 2B).

#### 3.1.2 Raw material characterisation

The elemental composition of the black manganese lumps is presented in Table E in S1 Table. Apart from a few pieces, the lumps present more than 30% of Mn. Only two lumps, both with a higher content of Si, feature a proportion of Mn of around 20%. Si, K, Ca, Ti, and Fe are present as major or minor components in all analysed samples. Several pieces have substantial concentrations of Ca, probably due to the secondary deposition or precipitation of calcium-rich compounds. Significant amounts of Si point to Si-based rocks or the occasional presence of quartz inclusions. All lumps present Ni, Zn, As, Sr, Ba and, to a lesser degree, Pb as trace elements. Varying amounts of Ba indicate that the pieces contain different proportions of complex Mn oxi-hydroxides. Coefficients of variation (CV) for Mn content (typically <10%) indicate that this element is homogenously distributed within the material. CVs for the other major and minor elements tend to be higher than for Mn, probably due to the presence of inclusions (i.e. quartz grains, anatase crystals, goethite nodules) or secondary surface enrichments (Table F in S1 Table). CVs for trace elements are highly variable, indicating different degrees of homogeneity in their distribution.

SEM-EDS were performed on three pieces that reflected the textural and compositional variation of the assemblage: MOU-MNP-04, a heavy, intensively modified nodule fragment, anthracite-grey in colour, composed of small crystals conferring the lump a characteristic metallic sheen; MOU-MNP-06, a light, unmodified brownish black powdery fragment with a dull appearance; and MOU-G&D-6857, a highly modified slightly porous dark grey fragment (Table G in S1 Table and Figures B, D and N in S2 Fig). MOU-MNP-04 shows a homogeneous microcrystalline texture and is composed of intertwined Mn crystals. Traces of Ba, Zn, and As were also detected in this specimen. MOU-MNP-06 is composed of a less homogenous material consisting of submicrometric acicular Mn/Ba-rich crystals surrounding isolated crystals of silicates and aluminosilicates. MOU-G&D-6857 features a heterogeneous texture and is composed of micrometric polygonal Mn-rich crystals surrounding larger irregular silicates crystals and aluminosilicate platelets. Acicular Mn/Ba-rich crystals are also present.

For conservation reasons, XRD analyses were conducted on flat surfaces of nine specimens. All the samples contain quartz and, with the exception of MOU-G&D-H7 and MOU-G&D-H8, calcite. Iron oxi-hydroxides, feldspars and phosphates were also occasionally identified (Table 3 and S2 Fig). The analysed pieces are composed of either a single Mn mineral (pyrolusite) or a mixture of simple (pyrolusite and manganite) and complex (birnessite, hollandite, romanèchite) Mn oxi-hydroxides. Mn-rich phosphates were also identified.

**Table 3:** Results of XRD analyses of Mn-rich lumps from Le Moustier.

Piece	Mineral phases containing Mn							Other mineral phases				
	Oxi-hydroxides		Complex oxi-hydroxides			Phosphates		Carb	Oxy-hydroxyd.	Phosph.	Silicates	
	Pyr	Man	Bir	Hol	Rom	Kas	Sid	Cal	Goe	Ber	Qz	Fsp
MOU-004												
MOU-012												
MOU-019												
MOU-020												
MOU-H7												
MOU-H8						* *						
MOU-6857												
MOU-9796												
MOU-11571												

Ber: berlinite; Bir: birnessite; Cal: calcite; Fsp: feldspar; Goe: goethite; Hol: hollandite; Kas: kastningite; Man: manganite; Pyr: pyrolusite; Rom: romanechite; Sid: sidorenkite; Qz: quartz.

Micro-RS, performed on seventeen specimens (Table 4 and S2 Fig) identified crystals of different colour (grey, dark grey, and black), sizes (sub-micrometric, micrometric) and morphology (amorphous, botryoidal, regularly and irregularly faceted) highlighting the complex nature of these materials. Molecular analysis confirmed the presence of simple (pyrochroite, pyrolusite, ramsdellite) and complex (hollandite, romanèchite, todorokite) Mn oxi-hydroxides. It also confirms that some samples (MOU-B-MNP-04, MOU-G3-MNP-19, MOU-G&D-5983) only contain a single Mn mineral phase (pyrolusite) while others several. We also detected carbon, hematite, muscovite, quartz and undetermined clay minerals.

**Table 4:** Results of μ-RS analyses of black lumps from Le Moustier.

Piece	N° of measur.	Morphology	Grain color	Identified compounds
MOU-MNP-03	6	Agglom.	Black	hol, pyr, rom
MOU-MNP-04	6	Reg. fac. cryst	Black	pyr
MOU-MNP-05	5	Agglom.	Black	hol, pyr, prc, qz
MOU-MNP-06	6	Agglom.	Dark grey	hol, tod
MOU-MNP-09	6	Tabular	Black	hol, pyr, C
MOU-MNP-11	6	Agglom.	Dark grey	hol, pyr
MOU-MNP-12	6	Agglom. of reg. fac. cryst.	Black	hol, pyr, tod (+ clay min.)
MOU-MNP-19	7	Agglom.; irreg. fac. cryst.	Dark grey; iridiscent	pyr; mus
MOU-MNP-20	6	Agglom.	Black	hol
MOU-MNP-L&R-H7	5	Irreg. fac. cryst.	Black	hol, pyr, prc, ram
MOU-MNP-L&R-H8	7	Agglom.	Black	pyr, hol?
MOU-G&D-5983	5	Agglom.	Dark grey	pyr
MOU-G&D-6857	11	Botryoid.; reg. fac. cryst.; agglom.; irreg. fac. cryst.	Dark grey	hol, pyr, prc; qz
MOU-G&D-7527	5	Agglom.	Dark grey	hol, pyr, pcr
MOU-G&D-9796	7	Botryoid.	Black	hol, prc
MOU-G&D-11571	9	Agglom.	Dark grey	hol, pyr
MOU-G&D-12622	10	Amorph.	Dark red; black; white	hol, prc, tod, pyr; hem; qz

Agglom.: agglomerate; amoprh.: amorphous; botryoid.: botryoidal; irreg. fac. cryst.: irregular facettes crystal; reg. fac. cryst.: regular facetted crystal; min.: minerals; undet.: undetermined. Key mineral phases—C: carbon; hem: hematite; hol: hollandite; mus: muscovite; prc: pyrochroite; pyr: pyrolusite; qz: quartz; ram: ramsdellite; tod: todorokite

#### 3.1.3 Technological analysis

**Fig 3:**
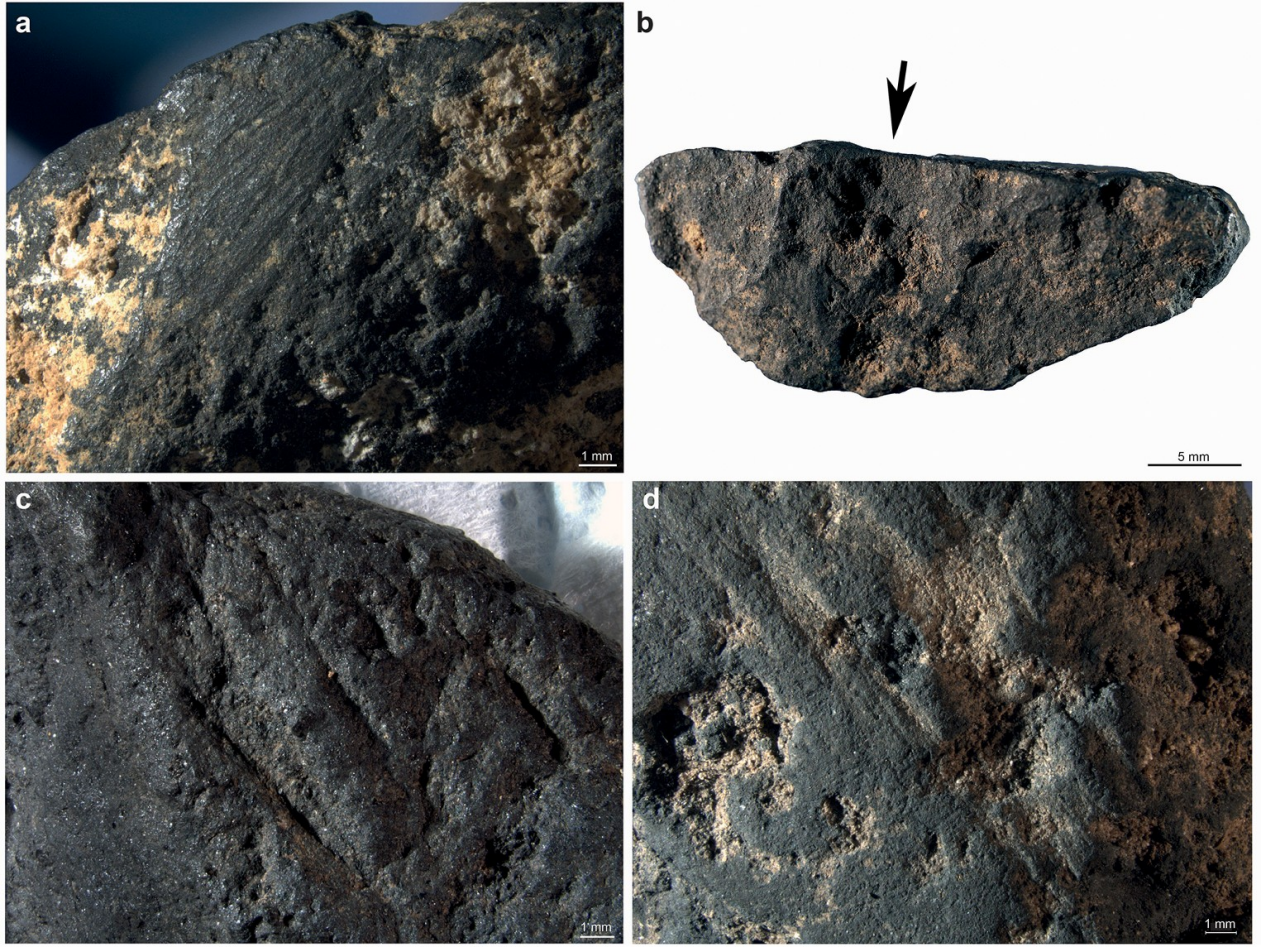
Traces of modifications identified on manganese-rich rocks. Modification types identified on manganese-rich rocks from Le Moustier. (a) Abrasion, (b) percussion, (c) notching, (d) scraping.

**Fig 4:**
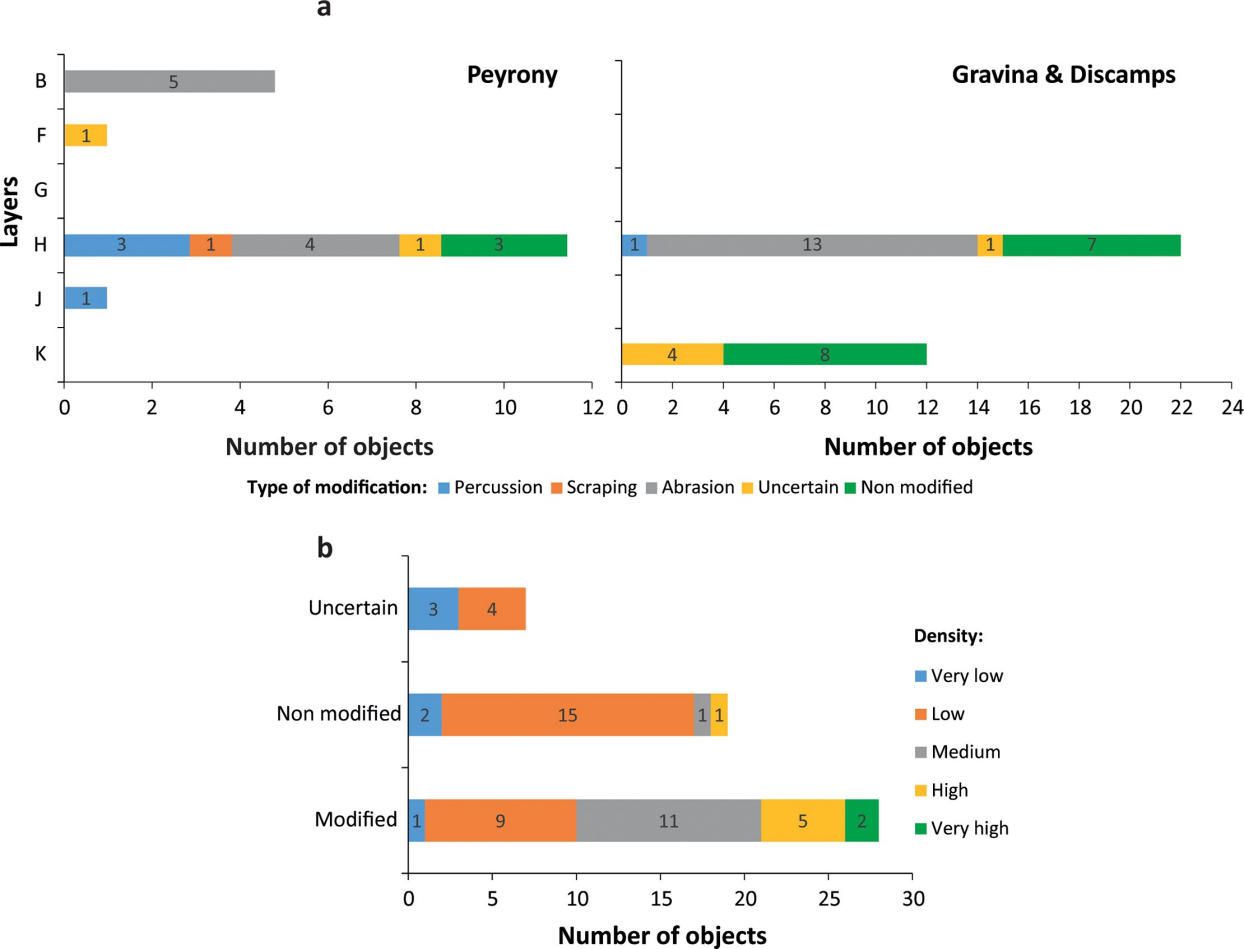
Modification types. Types of modification identified on manganese-rich lumps from Le Moustier by layer (a) and density (b). The arrow indicates the point of impact.

Four types of modifications, present on half of the pieces (n = 35; 64.8%), are evident on the lumps (Fig 3; Table H in S1 Table): facets covered by striations due to grinding (n = 20; 37%), facets bearing no visible striations (n = 5; 9.25%), flake scars produced by percussion (n = 5; 9.25%), striations and incisions made by a lithic point or a cutting-edge (n = 2; 3.7%). Highly polished areas are visible at high magnification on several facets with no striations. Pieces with facets generally bear between one and three facets (n = 21; 38.8%), few show up to six facets (n = 6, 11.11%). Most striated facets are slightly convex (n = 29), few are concave (n = 16) or flat (n = 12). The striations, indicating the direction of the grinding motion, are systematically oblique or parallel to the facet maximum length. On a limited number of facets, the striations (n = 5) feature two main preferential orientations indicate successive grinding motions/episodes. The absence of striations on some facets may be due to wear produced by use on a smooth surface after their production by grinding or obliteration due to taphonomic processes. The latter hypothesis is supported by the absence of pieces bearing facets with and without striations. The morphology and delineation of flake scars suggest they result from direct percussion, probably with a hard hammer. Some small pieces bear fractures produced by crushing larger fragments on an anvil. Of the two pieces showing incisions, one displays two converging notches produced by the back-and-forth movement of a cutting edge, the other few striations were made by a lithic point on a facet. The majority of the pieces only bear one type of modification (n = 27; 77%), and only a few associate two types: grinding and scraping, and grinding and percussion. The number of pieces available from each layer is too small to assess significant changes in processing techniques over time. While all of the recorded techniques coexist in layer H/H* Fig 4A, this “layer” reflects several distinct phases of occupation by potentially different Neanderthal groups [91]. We also observe that Neanderthals preferentially modified denser fragments Fig 4B. This may indicate that softer fragments were pounded rather that modified with techniques such as scraping or grinding, which leave detectable traces on the objects. Alternatively, denser pieces may have survived better after been modified by grinding, scraping or notching. With the exception of one highly-dense specimen, all other specimens bearing or possibly bearing flake scars have a rather low density, ranging from medium (n = 2) to low (n = 6) or very low (n = 2). This supports the idea that softer pieces were preferentially more often pounded.

Three pieces merit more detailed descriptions. The first (MOU-MNP-L&R-H7) consists of a tetrahedral fragment bearing two adjacent elongated faces overlain by multiple deep subparallel incisions made by a lithic point, and oriented along the main axis of the facets (Figure C in S1 Fig). The ridge between these two surfaces is cut perpendicularly by a tiny notch created by the edge of a stone tool. Sub-parallel incisions made by lithic points obliquely cross a third face of the object. The second (MOU-MNP-L&R-08) has a pyramidal shape (Figure D in S1 Fig), with the three finely ground surfaces creating linear ridges. A large portion of the base, which was originally also ground, has been removed by a fracture. The third object (MOU-G&D-6857, Figure E in S1 Fig) is pyramidal with a rectangular base. All faces and the base appear to have been shaped by grinding but bear no visible striations. At higher magnification, prominent areas show a metallic sheen.

#### 3.1.4 Morphometric analysis

**Fig 5:**
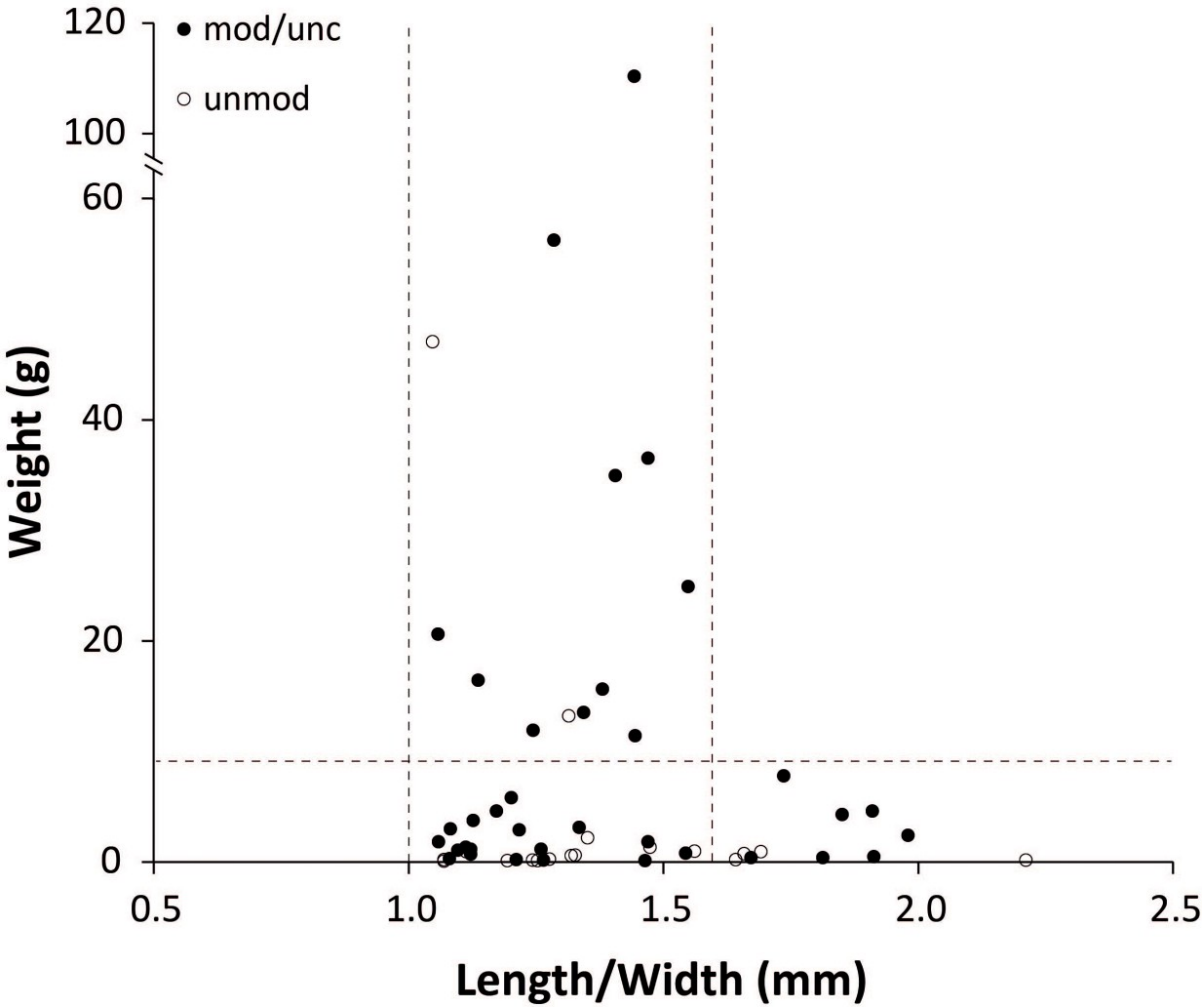
Weight vs. length/width ratio for manganese-rich rocks. Comparison between lump weight and length/width ratio. Dotted lines identify groups discussed in the text; mod/unc—modified/possibly modified; unmod—unmodified.

An interesting pattern emerges when the weight of the fragments is compared with their length/width ratio (Fig 5). All heavy fragments have a length/width ratio ranging between 1.1 and 1.6 while a large proportion of the light fragments are elongated (length/width ratio of between 1.6 and 2). Among the latter, and with the exception of a single outlier, all pieces with a length to width ratio of close to 2 are modified. A Mann-Whitney test confirms that the length/width ratio is significantly different (p = 0.0001) between modified and unmodified pieces. Two reasons may explain this pattern. The elongated and modified light fragments may either represent by-products of the processing of larger fragments or they already had an elongated shape when collected at the outcrops.

**Fig 6:**
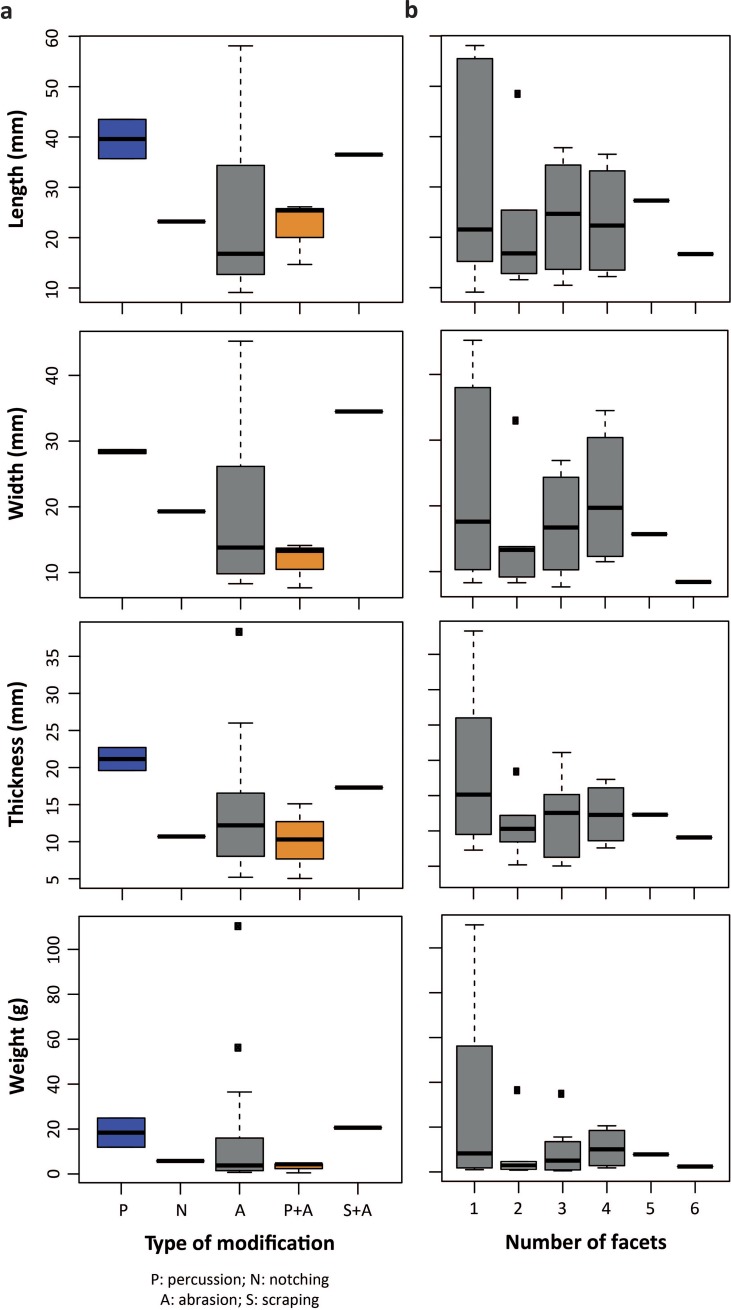
Attributes of the manganese-rich rocks. Size and weight of modified manganese-rich rocks from Le Moustier by type of modification (a) and number of facets (b). P—Percussion; N—notching; A—abrasion; S—scraping.

Differences in size and weight are evident between pieces bearing different types of modification (Fig 6A). Those with percussion marks are the largest, followed by pieces associating scraping and abrasion marks, and those with evidence for abrasion and percussion, and notching. This pattern may indicate that fragments were first pounded, then abraded, and in some cases pounded again. The piece bearing traces of scraping is an exception. Its larger size may be due to the fact that this technique can only be applied to relatively large pieces. Among the pieces bearing facets, those with just one facet are the largest and heaviest, followed by examples with three or four facets (Fig 6B). The comparatively small size of the pieces with two facets is difficult to explain since one would expect a gradual decrease in size and weight of pieces reduced by repeated grinding. When facetted objects are considered by layer, the larger pieces recovered by Peyrony in layers “B” and, less clearly, in layer “H” follow the expected trend, i.e. a decrease in size as a function of the number of facets, while this does not apply to specimens found during more recent excavations (Figure F in S1 Fig). This implies that Neanderthals imported both large and small pieces to the site. The former were gradually reduced by increasing the number of ground facets; the latter either were submitted to the same process or were only modified by grinding one or two facets.

#### 3.1.5 Relationship between size, modification, Mn content and stratigraphic provenance

**Fig 7:**
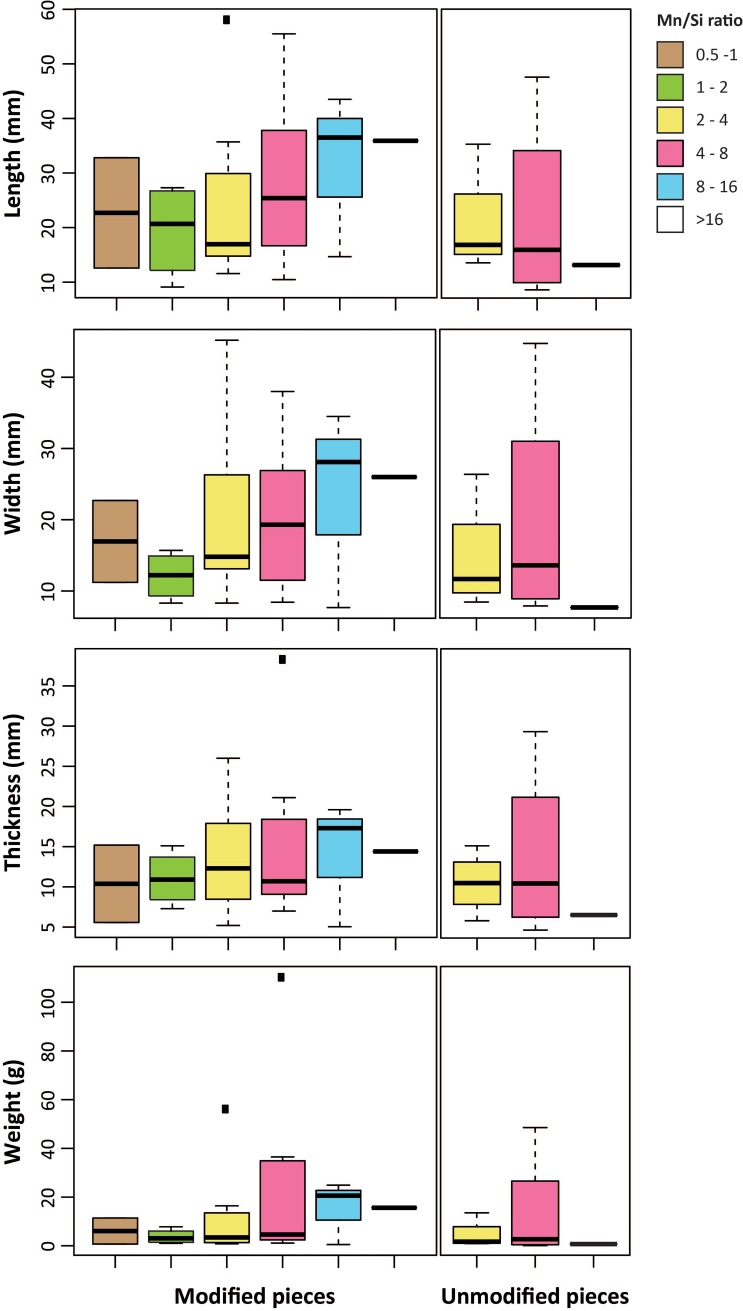
Mn/Si ratio of the manganese-rich rocks. Mn/Si ratio of modified and unmodified manganese-rich rocks from Le Moustier by size and weight.

Contrasting dimensional, technological and compositional data reveal interesting trends (Fig 7). In modified fragments, Mn content increases with size while no such trend is observed among unmodified fragments. Only modified pieces depict a low Mn content (Mn/Si ratio between 0.5 and 2). On the other hand, large lumps with high Mn content (Mn/Si ratio between 8 and 16) are overrepresented among the modified pieces. Unmodified pieces with a medium Mn content (Mn/Si ratio between 2 and 8) are substantially smaller than modified examples with a similar Mn content. Analysis of Mn content and object size per layer (Figure G in S1 Fig) shows that in layer H, large pieces, only found by Peyrony, are characterized by a medium to high Mn content. Small pieces, only recovered by G&D, predominantly feature both a low and medium Mn content. Although sample size is too small to reliably establish whether the other layers, for which we currently only have material from Peyrony’s excavations (B, F, G, J), follow the trend identified for layer H, it is worth noting that, with a single exception, the recovered pieces never show a low Mn content and most of the large examples have a medium to high Mn content. This reveals no substantial differences between layers in terms of this relationship between size and Mn content, a trend which is common to both modified and unmodified pieces. Among modified pieces, those poor in Mn or with a medium Mn content are preferentially modified by abrasion (Figure H-a in S1 Fig). Percussion is not applied to pieces with a very low and very high Mn content. Scraping and notching are only recorded on fragments with a high and very high Mn content respectively. Apart from a few exceptions, the number of facets increases in tandem with Mn content, suggesting that pieces richer in this element were more intensively scraped (Figure H-b in S1 Fig).

### 3.2. Geological samples

#### 3.2.1 Raw material appearance and composition

The geological survey identified nine types of Mn-rich deposits: 1) limestone, 2) low crystalline crusts covering the walls of karstic systems, 3) isolated lenses of sediment within karstic infill, 4) compact nodules and 5) clay deposits in caves, 6) clayey sand deposits filling dissolution features in limestone formations, 7) friable nodules in alluvial deposits, 8) patinas on cobbles and boulders from fluvial deposits, 9) compact nodules in open-air clay deposits.

Microscopic, elemental and mineralogical analyses show a high degree of variability in morphology, composition and mineral associations between and, to some degree, within these categories (Table J in S1 Table, Table 5). A description of the geological samples and relevant analyses are provided in S1 Text and S3 Fig.

**Table 5:** Results of XRD analyses of geological samples.

Region	Name of the outcrope	Type of Mn deposit	Mineral phases containing Mn										Other mineral phases							
			Oxides	Composed oxi-hydroxides					Phos.	Silicates			*Carb*.	Clays	Fe-rich hydr	Micas	Phosphates		Silicates	
			Pyr	Bir	Hol	Rom	Ran	Tod	Ear	Bem	Fer	Mgp	*Cal*	Kao	Goe	Mus	Ber	Hyd	Qz	Fsp
"Le Nontronais"	Tranche-couyère	Nodule	** **			** **	** **		** **	** **	** **	** **	* *	** **		** **	** **	** **		** **
	Teyjat	Patina	** **		** **	** **	** **		** **	** **	** **	** **	*** ***	** **	** **	** **	** **	** **		** **
Vézère—Dordogne valleys	Cave	Crust											* *							
	Loup												* *							
	Beyssac	Lens											* *							
	Sarlat	Clayey sandstone											* *							
	Theil	Limestone											* *							
	Verdier	Nodule											* *							
Dordogne—Lot valleys	Albas	Limestone											* *							
	Causse												* *							
	Sals												* *							
	Bouzic	Nodule											* *							

Mineral phases: Bem: bementite; Ber: bernalite; Bir: birnessite; Cal: calcite; Ear: earlshannonite; Fsp: felsdpar; Goe: goethite; Hol: hollandite; Hyd: hydroxyapatite; Kao: kaolinite; Mgp: manganopyrosmalite; Mus: muscovite; Pse: pseudobrokite; Pyr: pyrolusite; Ran: rancieite; Rom: romanechite; San: sanidine; Qua: quartz; Tod: todorokite.

SEM analyses and images in back-scattered electron mode of representative areas highlight clear differences between samples (Table J in S1 Table, and Figures A in S3 Fig). Specimens from *Mine d’Albas*, *Mine de Causse du Cluzel*, and *Mine de Sals* (type 1; Figures B to F in S3 Fig) share a large grain size range and are composed of both large blocky crystals (Ca-rich carbonates) and smaller lath- and needle-like crystals (Mn/Ba-rich compounds). Inter- and intra-granular regions of large crystals are filled with small-elongated crystals, generally organized in tree-like or radiating patterns, and less frequently as stacked platelets. Samples from *Mine de Le Theil* display a larger textural and compositional variability (type 1; Figures G to I in S3 Fig). Like the previously described outcrops, they share a broad grain size range including large crystals with clean edges (Ca-rich carbonate) and small Mn/Ba-rich platelets. Despite their geographic proximity and similar geological setting, the *Grotte-Cave* and *Grotte du Loup* samples (type 2) are different in terms of texture and composition. The sample from *Grotte-Cave* (CAV-01, Figure J in S3 Fig) is characterized by the presence of poorly crystallised Mn-rich mineral phases with three different morphologies: a gel-like mineralization (Mn, Si, Al, Ca, Fe, K) interpreted as a manganese silicate, spherical agglomerates of sub-micrometric irregular crystals (Fe, Mn, Si) probably consisting of a ferroan-manganese silicate, and amorphous masses (Mn, Ca, P), possibly a manganese phosphate. The sample from *Grotte du Loup* (LOU-01, Figure K in S3 Fig) is composed of two forms of poorly crystallized Mn-rich mineralisations: botryoidal agglomerates and amorphous masses with cracks. Contrary to the previous sample, its composition is rather homogeneous (Mn, Ca, Al, Si). Like the other samples from cave contexts, the lens of loose material sampled at *Grotte de Beyssac* (BEY-01, type 3; Figure L in S3 Fig) is characterised by a heterogeneous granulometry. However, it strongly differs in terms of grain size range, texture and composition. Mainly composed of carbonates and silicates, the grains from this sample are interspersed with irregular, needle- and lath-like crystals (Mn, Ba) and some form of aluminosilicates and phosphates (Si, Al, Na, Mg, and P). Samples collected at *Grotte du Trou du Vent* (BOU-03, 04, 06; types 4, 5 and 3 respectively; Figures M to O in S3 Fig) have variable textures and compositions. SEM-EDS shows that the BOU-03 nodule has a compact, Mn-rich external layer and a core composed of Ca-rich crystals embedded in a Fe/Si/Al-rich matrix. BOU-04, on the other hand, is a clayey sample containing an admixture of aluminosilicates (Fe, Mn, Si, Al), Ti-rich mineral phases, carbonates (Ca, Mg), rare earth minerals (Ce, La, Th, Nd), and carbon particles (C). BOU-06 is a crust with a botryoidal texture composed of an admixture of complex Mn/Ba-rich compounds, various Fe-rich aluminosilicates, and phosphates. Sample SAR-01, from *Sarlat* (type 6; Figure P in S3 Fig) clearly differs from the other samples from the Dordogne region: it is a black clayey sandstone composed of coarse rounded silicate grains (Si) coated by Mn compounds (Mn, Si, Ba, Ca) with different morphologies (platy or needle-like). The friable nodules from *Carrière Le Verdier* also show no textural or compositional similarities with samples from other outcrops (VER-01; type 7; Figure Q in S3 Fig). Instead, they consist of agglomerates of tiny spongy spheres composed of a Ca/Ba/K-rich manganese compound.

**Fig 8:**
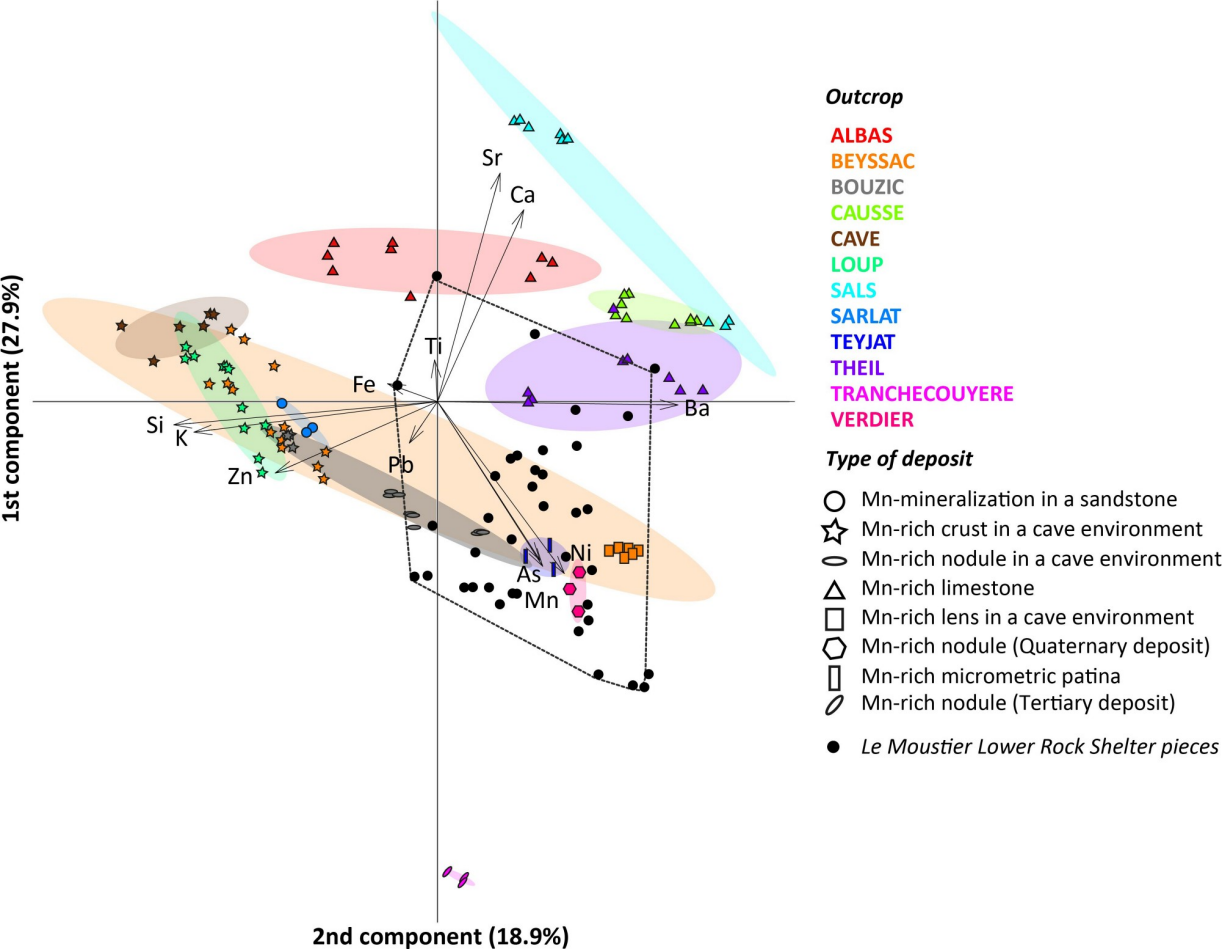
Principal component analysis of the manganese-rich geological samples. PCA using a centred logarithm ratio (clr) transformation of the twelve most frequently detected major, minor and trace elements by EDXRF in the geological samples. Colours and symbols indicate the outcrop origin and type of deposit, respectively. The archaeological samples (black dots) play no role in the PCA.

Elemental analyses by EDXRF (Table J in S1 Table) show that samples from *Carrière Le Verdier*, *Teyjat* (type 8; Figure R in S3 Fig), and *Grotte de Beyssac* have higher Mn concentrations of, respectively, 51%, 37% and 36%, compared to other geological sources, which show Mn concentrations ranging between 32% and 4%. A principal component analysis (Fig 8) reveals that 1) a number of outcrops differ significantly in their composition; 2) the variability observed within a single outcrop (*Grotte de Beyssac*) encompasses that observed in five other outcrops (*Grotte-Cave*, *Grotte du Loup*, *Grotte du Trou du Vent*, *Sarlat*, *Teyjat*, *Carrière Le Verdier*); 3) in five cases (*Mine d’Albas*, *Mine de Sals*, *Mine de Le Theil*, *Grotte de Beyssac*, *Grotte du Trou du Vent*) samples from the same outcrop cluster separately. We also observe that samples from similar geological formations share similar elemental compositions regardless their geographic origin. Mn mineralizations present in limestones (*Mines d’Albas*, *Causse du Cluzel*, *Sals*, and *Le Theil*) differ from those collected in other formations given their comparatively high content of Ca and Sr, and low content of Fe, K, and Si. Mn-rich crusts (*Grotte de Beyssac*, *Grotte-Cave*, *Grotte du Loup and Grotte du Trou du Vent*) are higher in K and Si. Mineralizations in sandstones (*Sarlat*) associate elements frequent in crusts with a higher Si content. The sample from an alluvial deposit (*Carrière Le Verdier*) is primarily composed of Mn with a low content of Ca, Fe, K and the absence of Si. Patinas on cobbles and boulders (*Teyjat*) associate a high Mn content with the greatest proportion of As. Nodules from Tertiary clay deposits (*Tranchecouyère*, type 9, Figure S in S3 Fig) are characterised by a unique composition, including Pb as diagnostic trace element. Ternary plots (Figure I in S1 Fig) clearly differentiate the *Grotte de Beyssac*, *Grotte-Cave*, and *Grotte du Loup* crusts, rich in Zn and Fe and poor in Ni, from all the other outcrops, which often cluster separately due to their variable Sr and Ba content.

X-ray diffraction shows no definite trends (Table 5 and S2 Fig). All samples contain quartz and, with the exceptions of *Teyjat* (TEY-01) and *Sarlat* (SAR-01), calcite. Kaolinite, goethite, muscovite, K-rich feldspars and, to a lesser extent, phosphates, such as berlinite and hydroxyapatite, are the other mineral phases identified. As far as Mn mineral phases are concerned, crusts from *Grotte-Cave* (CAV-01) and *Grotte du Loup* (LOU-01, -03, -4) contain a complex oxi-hydroxide (birnessite) and a silicate (bementite). The *Sarlat* sample primarily contains a Mn-rich simple oxide (pyrolusite). Limestones from the Vézère (*Mine de Le Theil*, THE-02, -03, -06) and Lot Valleys (*Mine d’Albas–*ALB-01, *Mine de Causse du Cluzel*–CAU-01, -02, -03, and *Mine de Sals–*SAL-01, -02, -03) are composed of several Mn mineral phases: simple (pyrolusite) and complex oxi-hydroxides (hollandite and romanèchite), a phosphate (earlshannonite) and a silicate (ferrobustamite). The lenses from *Grotte de Beyssac* (BEY-01, -03) are characterised by the simultaneous presence of simple (pyrolusite) and complex oxi-hydroxides (romanechite). The nodule from *Carrière Le Verdier* (VER-01) features a unique mineral association comprising complex oxi-hydroxides (rancieite and todorokite). The compact nodule from *Grotte du Trou du Vent* (BOU-06) contains simple (pyrolusite) and complex oxi-hydroxides (birnessite), while the *Tranchecouyère* nodule (TRA-01) incorporates three complex oxi-hydroxides (birnessite, hollandite, todorokite). The *Teyjat* patina is characterized by two complex oxi-hydroxides (birnessite, todorokite).

3.3. Sourcing the archaeological samples.

Plotting the Le Moustier pieces on the PCA describing the compositional variation of geological samples (Fig 8) reveals the archaeological pieces to be incompatible with six of the surveyed outcrops (*Grotte-Cave*, *Grotte du Loup*, *Sarlat*, *Mine de Sals*, *Mine de Causse du Cluzel*, and *Tranchecouyère*). Two other outcrops (*Mines d’Albas* and *Le Theil*) could also be excluded, as their similarity to a handful of archaeological outliers is likely due to the post-depositional incorporation of Ca into the latter. Samples from four outcrops (*Grotte de Beyssac*, *Carrière Le Verdier*, *Grotte du Trou du Vent*, and *Teyjat*) are compatible with the Le Moustier material in terms of composition. However, they differ in their morphology, texture, and mineralogical content. Compositionally compatible samples from *Grotte de Beyssac* consist of lenses of powdery sediment and those from *Teyjat* of micrometric coatings on cobbles and boulders. The friable nodules from *Carrière Le Verdier* are similar to several archaeological pieces in their overall morphology. Their microscopic texture and mineralogy, in particular the presence of rancieite and todorokite, rule out this outcrop being the source of the Le Moustier pieces. Samples from *Grotte du Trou du Vent* consist of friable cobbles, crusts and coatings on pebbles which are morphologically and texturally different from the archaeological pieces.

### 3.4. Compositional variability of Mn-rich rocks from Le Moustier

**Fig 9:**
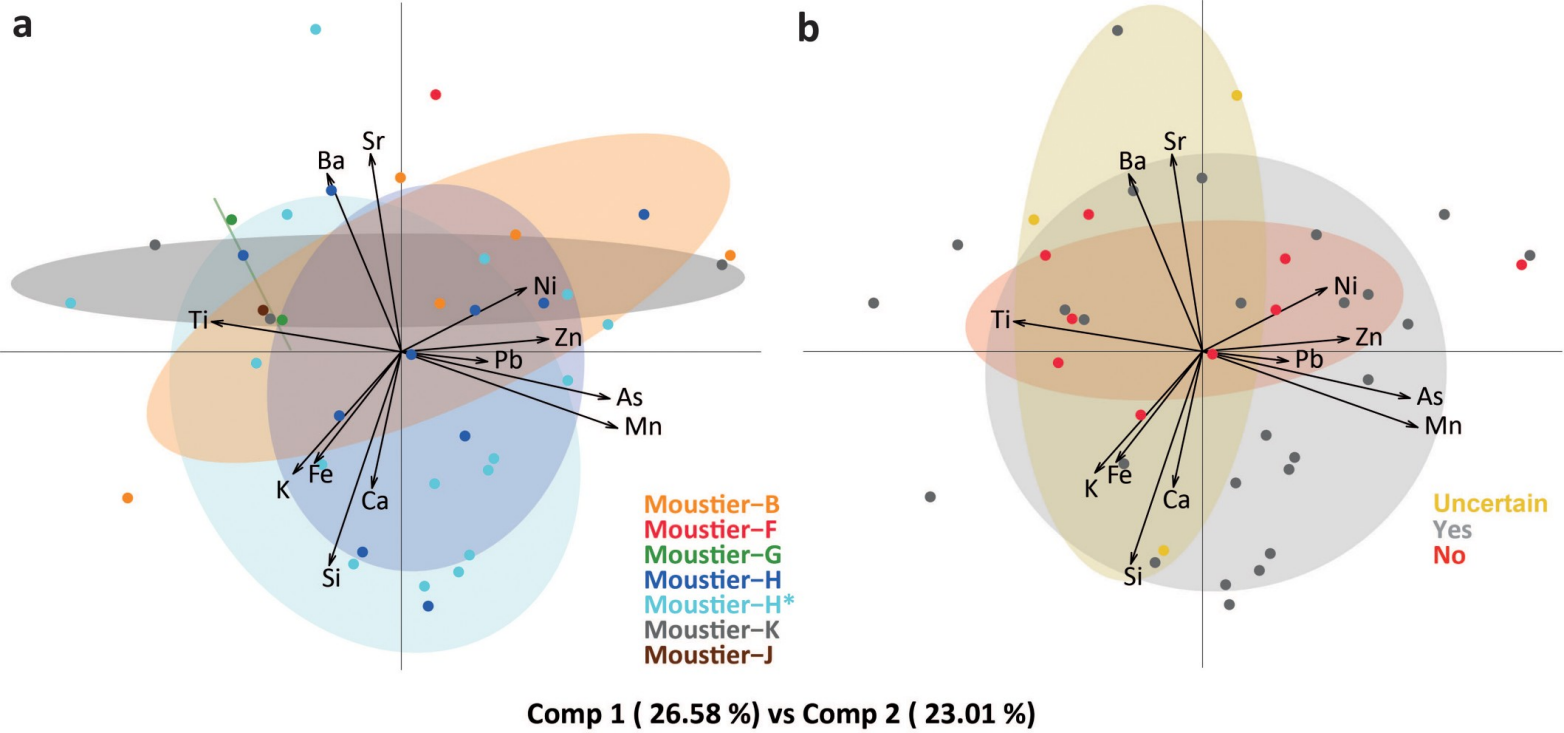
Principal component analysis of the archaeological manganese-rich rocks. PCA using a centred logarithm ratio (clr) transformation of the twelve most frequently detected major, minor and trace elements by EDXRF in the archaeological manganese-rich rocks from Le Moustier by layer (a) and modification occurrence (b).

No clear trends appear in the compositional variability of the archaeological pieces by layer (Fig 9A). The layer with the greatest sample size (layer H and H*) also features the highest variability, encompassing most of that observed in other layers, yet such a pattern could be expected considering the thickness of this “layer” and the possibility that it reflects multiple occupations by different Neanderthal groups. The pieces recovered during Peyrony’s excavations (layer H) fall within the variability of those from recent excavations (layer H*). It is, however, worth noting that pieces from layers B, F, G, K, and J are those with the lowest Mn, As, Pb, Si, and Ca content. Pieces bearing traces of modification show a higher degree of compositional variability, which include that recorded on unmodified pieces featuring the lowest Mn and As content (Fig 9B).

## 4. Discussion and conclusions

The integrated analysis of the Mn-rich lumps from Le Moustier provides clues for reconstructing the chain of choices and actions underlying the selection, transport, processing and use of these materials by Neanderthals. It is difficult to establish exactly how many Mn-rich lumps Neanderthals collected and used at Le Moustier. Our study demonstrates, not surprisingly, that pigment collections from old excavations give a highly biased picture of the importance of these materials for Mousterian groups. While Peyrony almost exclusively recovered large modified pieces, the exhaustive recovery protocol of G&D shows Mn-rich lumps from all the site’s archaeological layers to consist predominantly of small pieces and that only half of them bear traces of modification. In addition, comparison of the degree of modification between collections from successive excavations reveals that intensively modified objects are overrepresented in the Peyrony sample. Considering that a third of the fragments recovered by Peyrony comes from the same layers excavated by G&D, as well as substantial differences between the two excavations in terms of the excavated volume of sediments, a clear recovery biases most likely explains the striking difference in fragment size and degree of modification between the two collections. The virtual absence of large pieces recovered by G&D suggests instead that Mn-rich assemblages at Le Moustier are mostly composed of small pieces. The quantity of small fragments overlooked by Peyrony can be approximated if we assume comparable occurrences of Mn-rich lumps per layer and sediment volume. This assumption is supported by the fact that G&D recovered Mn-rich lumps in all layers so far excavated, including layer K, from which Peyrony recovered no lumps. The number of pieces overlooked by Peyrony should be equal to or higher than the number of pieces found during G&D’s excavation (n = 32) and less than that recovered by Peyrony (n = 14) multiplied by the number of pieces from the Peyrony’s excavation larger than those in the G&D sample (n = 12). The resulting figure of 168 almost certainly underestimates the real number Mn-rich lumps overlooked by Peyrony when the bias in favour of highly modified pieces that characterises the Peyrony sample is taken into account. This being the case, the number of pieces that Peyrony failed to collect is likely the double of our calculation. In addition, our study only concerns material from the 2015 and 2016 excavation seasons that concerned the summit of layer H. Hence, any changes in frequency of Mn-rich lumps across layer H could potentially alter this figure, which therefore remains a gross estimate based on currently available data.

Traces of modifications on the Mn-rich rocks reveal Neanderthals to have developed a dedicated technology to process this particular material, which involved a variety of tools (grindstones, hammers, anvils, pointed and sharp stone tools) and associated processing motions. These processing techniques appear adapted to the size and density of the raw material, with several techniques applied successively or in alternation on the same piece. The relationship between the presence of modifications and raw material density is consistent with the hypothesis that softer fragments were pounded rather than modified by, for example, scraping or grinding, which leave detectable traces. Interestingly, this trend departs from that documented for Châtelperronian pigments [75], where percussion seemed to have been preferentially applied to harder raw materials.

Morphological, textural and chemical differences between geological and archaeological samples suggest that Neanderthals did not collect Mn-rich lumps at the outcrops sampled in this study. Sources exploited by Mousterian groups may since have been eroded, buried or destroyed by, for example, intense nineteenth-century mining activities in the Dordogne. The alternative hypothesis is that the sources of the Le Moustier pigments are located outside the surveyed region. In sum, while the exact provenance of the Mn-rich rocks exploited by Neanderthal groups at Le Moustier remains to be identified, the compositional variability of the fragments from the sampled outcrops provides a means to infer the potential criteria informing the choice of Mn-rich lumps by Neanderthal groups. On the one hand, our results confirm a clear preference for lumps with high Mn content, uncommon in the sampled outcrops [78, 86]. The Mn content/size relationship is consistent with the hypothesis that material with a low Mn content was close to the site and, when collected, was immediately used while those with a higher Mn content come from more distant sources and were imported to the site either already modified or unmodified and, if not modified on the site, were lost or discarded without being used. On the other hand, Mn is systematically associated at Le Moustier with Ni, As and, at to a lesser extent, Ba. Calcium cannot be considered a reliable indication of provenience since its variable proportion almost certainly results from post-depositional enrichment in this element. The fact that Ca was not detected by SEM-EDS analyses of post-depositional fractures but often identified on pristine surfaces analysed by XRD supports this conclusion. The association and variability in Mn, Ni, As, Ba content at Le Moustier, compared to that observed at the sampled outcrops, suggests that either the Le Moustier lumps come from a unique source with a broad variation in composition, associating Mn, Ni, As, Ba, or that they were collected at different sources, some of which characterized by Mn-Ni-As, and others by Mn-Ba. A larger archaeological sample and data from more outcrops would be necessary to firmly tease apart these two hypotheses. However, diachronic changes in raw material selection suggest the latter to be more likely. Although sample size prevents us from drawing solid conclusions, most pieces from layer H/H* are small, modified, and feature the lowest Ba content. This pattern, which cannot be attributed to recovery bias since most of these pieces come from recent excavations, is consistent with the hypothesis that Neanderthals groups responsible for the formation of layer H/H* had access to lumps poor in Ba and processed them with techniques leaving detectable traces of modification. Moreover, it is likely that these pieces were collected at a source different from that exploited during the accumulation of the other layers (B, F, G, K, J), where Neanderthals collected lumps from sources with a higher Ba content and slightly poorer in Mn. The presence in layer H/H* of a limited number of pieces with a higher Ba content is compatible with the idea that the potentially different Neanderthal groups associated with layers H/H* also exploited this source, although at a lesser extent. This may indicate that Neanderthal groups responsible for the accumulation of layer H/H* exploited a larger territory or traded higher quality Mn material with other groups. It is equally worth noting that the observed difference in composition between these layers corresponds to differences in lithic technology. The recent reassessment of the lithic assemblages from Le Moustier attributed layer H to the Discoid lithic techno-complex and divided layer G into an upper bifacial (G3-G4) and lower Levallois (G1-G2) occupations [90, 92]. In addition, we observe that most of the pieces from layer B, attributed by Peyrony to a “Typical Mousterian”, feature a higher content of Ni and Sr, which may indicate they come from another source. If these differences were confirmed by future analyses of a larger sample, Le Moustier would represent the first Neanderthal site in which changes in colouring material composition correspond to changes in lithic technology. Concomitant changes in different aspects of Neanderthal behaviour would reinforce the idea that Mousterian stone tool technologies reflect both cultural traditions and elements of site function, two aspects of Neanderthals behaviour that still remain difficult to completely dissociate. Additionally and/or alternatively, changes in mineral pigment provisioning could be connected to environmental changes reducing accessibility to some outcrops while giving access to others. However, the reanalysis of the faunal assemblages from the top of layer G (G3-G4) and the lower part of layer H does not support the idea that the documented shift in lithic technology corresponds to a substantial environmental change, as both assemblages are dominated by species most often associated with temperate and closed biotopes [90, 93, 97]. The interpretation of changes in Mn fragments acquisition strategies potentially reflecting cultural changes would be reinforced by associated variations in processing techniques. However, our results remain, in this respect, ambiguous, and no clear differences in the use of these techniques could be observed between layers, which would suggest a degree of continuity in the way Mn-rich rocks are processed. Considering the small sample size and biases connected to differing recovery protocols, it would be premature to conclude that no such changes occurred at Le Moustier. It has been recently shown that gradual technological changes in the processing of colouring material can only be firmly identified at sites that have yielded large assemblages [109].

What purpose or purposes did these lumps serve? Body and skin decoration, camouflage, preservation of perishable materials, use in a “Female Cosmetic Coalition” (FCC) and fire ignition are the main explanations for the presence of Mn-rich black lumps at Mousterian sites (see [32, 78] for a synthesis). Body and skin decoration [40, 85] has been suggested based on experiments showing that Mn-rich lumps can be used to draw lines on soft materials, such as skin or hides, and that facets and use-wear similar to those produced experimentally for this purpose are found on archaeological black lumps. Camouflage [83–84] and the preservation of perishable materials [83] rely on broad comparisons with practices observed ethnographically. The proponents of the FCC model consider the use of black pigments by Neanderthals to reflect a climatic adaptation [110]: female African modern humans would have used red pigments during the Middle Stone Age as a strategy of cosmeticization of menstrual signals. Neanderthal females would have followed the same strategy only during interglacials, favoring pair bonds and suppressing cosmetic signaling during more severe episodes of glacial cycles. This hypothesis would be supported by a degree of correlation between black/red pigment ratio recorded at Mousterian sites and climate change. The recent discovery, however, of a large assemblage of modified red and yellow ochre at Les Bossats [72, 77, 80], a Middle Palaeolithic site in the north of France dated to MIS 3, is inconsistent with the FCC hypothesis. The ignition accelerant hypothesis [32, 86] is based on combustion experiments demonstrating pyrolusite (MnO_2_) rich lumps reduce the auto-ignition temperature of wood. The predominant presence of this mineral in 23 of the 24 lumps analyzed out of a collection of around 450 pieces recovered in the late Mousterian layers of Pech-de-l’Azé I (Dordogne, France), and the presence in this same region of three outcrops at which Mn-rich lumps contain small amounts of pyrolusite [99], would reinforce this interpretation, as it would, according to the authors of this study, suggest that Neanderthals preferentially collected pyrolusite-rich fragments. The problem with this interpretation is that of the three outcrops poor in pyrolusite cited by these authors and studied in [99], one (*La Pagésie*) is relatively far from Pech-de-l’Azé I (c. 30 km), which opens the possibility that distance rather than composition was the reason that this source was not exploited. At another outcrop (*Le Theil*), located close to the site and sampled by us, the hardness and texture of Mn-rich lumps make them unsuitable for other uses such as body or skin decoration and camouflage. Therefore, it is possible that black lumps were not collected from this outcrop due to their physical properties rather than their composition. Although we did not have access to lumps from the third outcrop (*Pech de Bord*), chances are high, due to its proximity and similar geological setting to *Le Theil*, that Mn lumps available at this outcrop are comparable to those from *Le Theil*, i.e. that they may have not been chosen by Neanderthal for reasons other than the absence of pyrolusite. Although the above does not absolutely rule out the possibility that Mn-rich lumps were used for fire ignition, it shows that this hypothesis is, at present, only based on the observation that pyrolusite-rich lumps are suitable for this function and not on geological data, i.e. the availability at close outcrops of lumps texturally similar to those found at archaeological sites and poor in pyrolusite that were not used by Neanderthals. Finally, while true that pyrolusite-rich minerals can serve as an accelerant for fire-starting, this does not imply that they were sought uniquely for this function.

Our analysis documented different processing techniques (abrasion, percussion, notching, scraping) at Le Moustier. Experimental reproduction of these techniques shows that each produces a powder of different coarseness and, in the case of abrasion, of different shades and compositions [56, 109, 111] due to the inclusion of variable proportions of particles derived from the grindstones into the resulting powder. This appears more consistent with multiple rather than a single function. Furthermore, three Mn-rich lumps from Le Moustier display modifications that are inconsistent with the fire ignition hypothesis. The convergent notches on MOU-MNP-L&R-H7 produced only a small quantity of powder, indicating these features were potentially designed to leave visible marks on the object’s surface of. As a consequence, they may represent abstract marking to which some sort of meaning was attached, such as ownership, rather than having a utilitarian purpose. A facet on MOU-G&D-6857 bears a sheen that may result from rubbing the piece against a soft material. MOU-MNP-L&R-H8 was shaped by grinding to create a tiny pyramid. The tip bears a polish indicating that the object may have been used as a crayon, which could support a symbolic use.

In sum, the proponents of the fire ignition hypothesis admit that it does not rule out concomitant functional and symbolic uses. In addition, our results show the fire-starting hypothesis does not to fully account for the currently available evidence. This is further supported by the fact that Neanderthals also used red and yellow iron-oxide rich rocks containing no pyrolusite and, at some sites, such as Scladina [73], used black siliceous graphitic siltstones, in which pyrolusite is also absent.

Future studies should focus on whether particular raw materials, processing techniques and functions are preferentially associated with distinct Mousterian flake production systems or lithic techno-complexes. Available evidence suggests that there is no strict correspondence between pigment colours, types, and lithic assemblages. Mn-rich lumps are, for example, associated with a discoidal technology at Le Moustier, with Quina, Levallois and Discoid technologies at Combe Grenal [8, 74], with Bordes’ Mousterian of Acheulean Tradition at Pech-de-l’Azé I and IV (see [8, 40] although see [90, 92] for questions concerning the validity of this “facies”), and Levallois technology at Pech-de-l’Azé IV and Caminade-Est [8, 112]. However, drawing definitive conclusions on this issue would require additional sites from southwestern France to benefit from the same type of approach presented here. Such an effort is unfortunately confounded by the fact that the stratigraphy of many key sites and technological attribution of numerous lithic assemblages currently require reassessment and that, as demonstrated by our analysis of the Le Moustier pigments, only new excavations allow the integrity of museum collections to be fully appreciated.

## Supporting information

S1 Table Supplementary data on the EDXRF calibration, the archaeological materials and the geological samples. (PDF)

S1 Fig Additional information on the EDXRF calibration, the archaeological Mn-rich lumps from Le Moustier and the Mn-rich geological samples collected at Dordogne and Lot regions of France. (PDF)

S2 Fig Results of analyses conducted on Mn-rich archaeological pieces from Le Moustier. (PDF)

S3 Fig Results of analyses conducted on Mn-rich geological samples from Dordogne and Lot regions of France. (PDF)

S1 Text Data on Mn-rich samples from geological sources including information on provenance, geological context, textural, elemental, and mineralogical composition. (PDF)

## References

[1] Bordes F. Essai de classification des industries « moustériennes ». Bull Soc Préhist France 1953; 50 (7/8): 457–466.

[2] Delagnes A, Jacques J, Meignen L. Les technocomplexes du Paléolithique moyen en Europe occidentale dans leur cadre diachronique et géographique In: Vandermeersch B, Maureille B, editors. Les Néandertaliens. Biologie et cultures. Editions du CTHS; 2007 pp. 213–229.

[3] Freeman LG. The nature of Mousterian facies in Cantabrian Spain. Am. Anthropol. 1966; 68 (2): 230–237.

[4] Mellars PA. The Neanderthal Legacy: An Archaeological Perspective from Western Europe. Princeton University Press; 1995.

[5] Monnier GF, Missal K. Another Mousterian Debate? Bordian facies, chaîne opératoire technocomplexes, and patterns of lithic variability in the western European Middle and Upper Pleistocene. Quat Int. 2014; 350: 59–83.

[6] Oakley K, Andrews P, Keeley L, Clark J. 1977 A reappraisal of the Clacton spearpoint. Proc Prehist Soc. 43:13–30

[7] Bordes F. Sur l'usage probable de la peinture corporelle dans certaines tribus moustériennes. Bull Soc Préhist France. 1952; 49 (3–4): 169–171.

[8] Demars PY. Les colorants dans le Moustérien du Périgord. L'apport des fouilles de F. Bordes. Préhistoire Ariègeoise 1992; 47: 185–194.

[9] Marshack A. Some implications of the Paleolithic symbolic evidence for the origin of language. Curr Anthropol. 1976; 17 (2): 274–282.

[10] Delporte H. Le Grand Abri de ta Ferrassie: Fouilles 1968–1973. Etudes Quaternaires 1981; 7.

[11] Peyrony D. Les moustériens, inhumaient-ils leurs morts? Bull Soc Hist Archéol Périgord 1921; 48: 132–139.

[12] Peyrony D. La Ferrassie. Préhistoire 1934; 3: 1–92.

[13] d’Errico F, Villa P. Holes and grooves: the contribution of microscopy and taphonomy to the problem of art origins. J Hum Evol. 1997; 33: 1–31. 10.1006/jhev.1997.0141 9236076

[14] Colagè I, d'Errico F. Culture: The Driving Force of Human Cognition. Top Cogn Sci. 2018; 1–19. 10.1111/tops.12372 30033618

[15] Majkić A, Evans S, Stepanchuk V, Tsvelykh A, d’Errico F. A decorated raven bone from the Zaskalnaya VI (Kolosovskaya) Neanderthal site, Crimea. PloS ONE 2017; 12 (3): e0173435 10.1371/journal.pone.0173435 28355292 PMC5371307

[16] Majkić A, d’Errico F, Milošević S, Mihailović D, Dimitrijević V. Sequential incisions on a cave bear bone from the Middle Paleolithic of Pešturina Cave, Serbia. J Archaeol Meth Th 2018; 25 (1): 69–116.

[17] Villa P, Roebroeks W. Neandertal demise: an archaeological analysis of the modern human superiority complex. PLoS ONE 2014; 9 (4): e96424 10.1371/journal.pone.0096424 24789039 PMC4005592

[18] Zilhão J. Chapter 4—Personal Ornaments and Symbolism Among the Neanderthals, in: Elias S. (Ed.) Origins of Human Innovation and Creativity. Developments in Quaternary Sciences 2012; 16: 35–49.

[19] Hardy BL, Moncel M-H. Neanderthal Use of Fish, Mammals, Birds, Starchy Plants and Wood 125–250,000 Years Ago. PLoS ONE 2011; 6: e23768 10.1371/journal.pone.0023768 21887315 PMC3161061

[20] Hardy K, Buckley S, Collins MJ, Estalrrich A, Brothwell D, Copeland L. et al Neanderthal medics? Evidence for food, cooking, and medicinal plants entrapped in dental calculus. Naturwissenschaften 2012; 99: 617–626. 10.1007/s00114-012-0942-0 22806252

[21] Henry AG, Brooks AS, Piperno DR. Microfossils in calculus demonstrate consumption of plants and cooked foods in Neanderthal diets (Shanidar III, Iraq; Spy I and II, Belgium). Proc Natl Acad Sci USA 2011; 108: 486–491. 10.1073/pnas.1016868108 21187393 PMC3021051

[22] Weyrich LS et al Neanderthal behaviour, diet, and disease inferred from ancient DNA in dental calculus. Nature 2017; 544: 357–361. 10.1038/nature21674 28273061

[23] Aranguren B, Revedin A, Amico N, Cavulli F, Giachi G, Grimaldi S, Macchioni N, Santaniello F. Wooden tools and fire technology in the early Neanderthal site of Poggetti Vecchi (Italy). Proc Natl Acad Sci USA 2018; 115 (9): 2054–2059. 10.1073/pnas.1716068115 29432163 PMC5834685

[24] Thieme H. Lower Palaeolithic hunting spears from Germany. Nature 1997; 385: 807–810. 10.1038/385807a0 9039910

[25] Schoch WH, Bigga G, Böhner U, Richter P, Terberger T. New insights on the wooden weapons from the Paleolithic site of Schöningen. J Hum Evol. 2015; 89: 214–225. 10.1016/j.jhevol.2015.08.004 26442632

[26] Soressi M, McPherron SP, Lenoir M, Dogandzic T, Goldberg P, Jacobs Z, Maigrot Y, Martisius NL, Miller CE, Rendu W, Richards M, Skinner MM, Steele TE, Talamo S, Texier J-P. Leather process Neandertals made the first specialized bone tools in Europe, Proc Natl Acad Sci USA 2013; 110 (35): 14186–14190. 10.1073/pnas.1302730110 23940333 PMC3761603

[27] Daujeard C, Valensi P, Fiore I, Moigne A-M, Tagliacozzo A, Moncel M-H et al A reappraisal of Lower to Middle Palaeolithic bone retouchers from Southeastern France (MIS 11 to 3). The Origins of Bone Tool Technologies. Mainz: RGZM; 2017 pp. 1–40.

[28] Mallye J-B, Thiébaut C, Mourre V, Costamagno S, Claud É, Weisbecker P. The Mousterian bone retouchers of Noisetier Cave: experimentation and identification of marks. J Archaeol Sci. 2012; 39: 1131–1142.

[29] Patou-Mathis M, Schwab C. Fiche générale In: Patou-Mathis M, editor. Retouchoirs, Compresseurs, Percuteurs Os à impressions et éraillures Fiches de la Commission de Nomenclature sur l’Industrie de l’Os Préhistorique Cahier X. Paris: Éditions de la Société Préhistorique Française; 2002 pp. 11–19.

[30] Romandini M, Cristiani E, Peresani M. 2014 A retouched bone shaft from the late Mousterian at Fumane cave (Italy). Technological, experimental and micro-wear analysis. Comptes Rendus Palevol. 14: 63–72.

[31] Roebroeks W, Villa P. On the earliest evidence for habitual use of fire in Europe. Proc Natl Acad Sci USA 2011; 108 (13): 5209–5214. 10.1073/pnas.1018116108 21402905 PMC3069174

[32] Sorensen AC, Claud E, Soressi M. Neandertal fire-making technology inferred from microwear analysis. Scientific reports 2018; 8 (1): 10065 10.1038/s41598-018-28342-9 30026576 PMC6053370

[33] Kozowyk PRB, Soressi M, Pomstra D, Langejans GHJ. Experimental methods for the Palaeolithic dry distillation of birch bark: implications for the origin and development of Neandertal adhesive technology. Sci Rep. 2017; 7 (1): 8033 10.1038/s41598-017-08106-7 28860591 PMC5579016

[34] Mazza PPA, Martini F, Sala B, Magi M, Colombini MP, Giachi G. et al A new Palaeolithic discovery: tar-hafted stone tools in a European Mid-Pleistocene bone-bearing bed. J Archaeol Sci. 2006; 33 (9): 1310–1318.

[35] d’Errico F, Doyon L, Zhang S, Baumann M, Lázničková-Galetová M, Gao X, Chen F, Zhang Y. The origin and evolution of sewing technologies in Eurasia and North America. J Hum Evol. 2018; 125: 71–86. 10.1016/j.jhevol.2018.10.004 30502899

[36] Hoffmann DL, Angelucci DE, Villaverde V, Zapata J, Zilhão J. (a). Symbolic use of marine shells and mineral pigments by Iberian Neandertals 115,000 years ago. Sci Adv. 2018; 4 (2): eaar5255 10.1126/sciadv.aar5255 29507889 PMC5833998

[37] Lhomme V, Freneix S. Un coquillage de bivalve du maastrichtien-paléocène Glyptoactis (Baluchicardia) sp. dans la couche inférieure du gisement moustérien de «chez-Pourré-chez-Comte» (Corrèze). Bull Soc Préhist Franç. 1993; 90: 303–306.

[38] Moncel MH, Chiotti L, Gaillard C, Onoratini G, Pleurdeau D. Non-utilitarian lithic objects from the European Paleolithic. Archaeol Ethnol Anthropol Eurasia 2012; 40: 24–40.

[39] Peresani M, Dallatorre S, Astuti P, Dal Colle M, Ziggiotti S, Peretto C. 2014 Juggling the interpretation of Neanderthal behaviour from symbolic to utilitarian. New inferences from the study of engraved stone surfaces. J Anthrop Sci. 92: 233–255. 10.4436/JASS.92007 25020018

[40] Soressi M, d’Errico F. Pigments, gravures, parures: les comportements symboliques controversés des Néandertaliens In: Vandermeersch B, Maureille B, editors. Les Néandertaliens. Biologie et Cultures. Paris: Éditions du CTHS. Documents Préhistoriques 23; 2007 pp. 297–309.

[41] Zilhão J. The emergence of ornaments and art: an archaeological perspective on the origins of “Behavioral Modernity”. J Archaeol Res 2007; 15: 1–54.

[42] Carciumaru M, Niţu E-C, Cîrstina O. A geode painted with red ochre by the Neanderthal man. Comptes Rendus Palevol. 2015; 14 (1): 31–41.

[43] Peresani M, Vanhaeren M, Quaggiotto E, Queffelec A, d’Errico F. An ochered fossil marine shell from the Mousterian of Fumane Cave, Italy. PLoS One 2013; 8 (7): e68572 10.1371/journal.pone.0068572 23874677 PMC3707824

[44] Zilhão J, Angelucci DE, Badal-García E, d’Errico F, Daniel F. et al. Symbolic use of marine shells and mineral pigments by Iberian Neanderthals. Proc Natl Acad Sci USA 2010; 107 (3): 1023–1028. 10.1073/pnas.0914088107 20080653 PMC2824307

[45] Pettitt P. The Palaeolithic Origins of Human Burial. New York: Routledge 2011.

[46] Rendu W, Beauval C, Crevecoeur I, Bayle P, Balzeau A, Bismuth T, Bourguignon L, Delfour G, Faivre J-Ph, Lacrampe-Cuyaubère F, Tavormina C, Todisco D, Turq A, Maureille B. Evidence supporting an intentional Neandertal burial at La Chapelle-aux-Saints. Proc Natl Acad Sci USA 2014; 111 (1): 81–86. 10.1073/pnas.1316780110 24344286 PMC3890882

[47] Smirnov Y. Intentional human burial: Middle Paleolithic (last glaciation) beginnings. J World Prehist. 1989; 3: 199–233.

[48] Smirnov YA. Mousterian burials in Eurasia. Moscow: Nauka 1991. (In Russian).

[49] Zilhão J. Lower and Middle Palaeolithic Mortuary Behaviours and the Origins of Ritual Burial In: Renfrew C, Boyd MJ, Morley I, editors. Death Rituals, Social Order and the Archaeology of Immortality in the Ancient World. Cambridge: Cambridge University Press; 2016 pp. 27–44.

[50] Rodríguez-Vidal J, d’Errico F, Pacheco FG, Blasco R, Rosell J, Jennings RP, et al A rock engraving made by Neanderthals in Gibraltar. Proc Natl Acad Sci USA 2014; 111: 13301–13306. 10.1073/pnas.1411529111 25197076 PMC4169962

[51] d'Errico F, Doyon L, Colagé I, Queffelec A, Le Vraux E, Giacobini G, Vandermeersch B, Maureille B. From number sense to number symbols. An archaeological perspective. Phil Trans R Soc B Biol Sci. 2018; 373 (1740): 20160518. 10.1098/rstb.2016.0518 PMC5784044 29292345

[52] Finlayson C, Brown K, Blasco R, Rosell J, Negro JJ, Bortolotti GR. et al. Birds of a Feather: Neanderthal Exploitation of Raptors and Corvids. PLoS ONE 2012; 7 (9): e45927 10.1371/journal.pone.0045927 23029321 PMC3444460

[53] Morin E, Laroulandie V. Presumed Symbolic Use of Diurnal Raptors by Neanderthals. PLoS ONE 2012; 7: e32856 10.1371/journal.pone.0032856 22403717 PMC3293908

[54] Peresani M, Fiore I, Gala M, Romandini M, Tagliacozzo A. Late Neandertals and the intentional removal of feathers as evidenced from bird bone taphonomy at Fumane Cave 44 ky B.P., Italy. Proc Natl Acad Sci USA 2011; 108: 3888–3893. 10.1073/pnas.1016212108 21368129 PMC3054018

[55] Radovčić D, Sršen AO, Radovčić J, Frayer DW. Evidence for Neandertal Jewelry: Modified White-Tailed Eagle Claws at Krapina. PLoS ONE 2015; 10: e0119802 10.1371/journal.pone.0119802 25760648 PMC4356571

[56] Rifkin RF. Processing ochre in the Middle Stone Age: Testing the inference of prehistoric behaviours from actualistically derived experimental data. J Anthrop Archaeol. 2012; 31: 174–195.

[57] Romandini M, Fiore I, Gala M, Cestari M, Guida G, Tagliacozzo A, Peresani M. Neanderthal scraping and manual handling of raptors wing bones: Evidence from Fumane Cave. Experimental activities and comparison. Quat Int. 2016; 421: 154–172.

[58] Romandini M, Peresani M, Laroulandie V, Metz L, Pastoors A, Vaquero M, et al Convergent Evidence of Eagle Talons Used by Late Neanderthals in Europe: A Further Assessment on Symbolism. PLoS ONE 2014; 9 (7): e101278 10.1371/journal.pone.0101278 25010346 PMC4092065

[59] Jaubert J, Verheyden S, Genty D, Soulier M, Cheng H, Blamart D, Burlet C, Camus H, Delaby S, Deldicque D, Edwards RL, Ferrier C, Lacrampe-Cuyaubère F, Lévêque F, Maksud F, Mora P, Muth X, Régnier É, Rouzaud JN, Santos F. Early Neanderthal constructions deep in Bruniquel Cave in southwestern France. Nature 2016; 534 (7605): 111–114. 10.1038/nature18291 27251286

[60] Roebroeks W., Sier M. J., Nielsen T. K., De Loecker D., Parés J. M., Arps C. E., Mücher H. J. Use of red ochre by early Neandertals. Proc Natl Acad Sci USA 2012; 109 (6): 1889–1894. 10.1073/pnas.1112261109 22308348 PMC3277516

[61] Hoffmann DL, Standish CD, García-Diez M, Pettitt PB, Milton JA, Zilhão J, Alcolea-González JJ, Cantalejo-Duarte P, Collado H, de Balbín R, Lorblanchet M, Ramos-Muñoz J, Weniger G-Ch, Pike AWG. (b). U-Th dating of carbonate crusts reveals Neandertal origin of Iberian cave art. Science 2018; 359 (6378): 912–915. 10.1126/science.aap7778 29472483

[62] Hoffmann DL, Standish CD, Pike AW, García-Diez M, Pettitt PB, Angelucci DE, Villaverde V, Zapata J, Milton JA, Alcolea-González J, Cantalejo-Duarte P, Collado H, de Balbín R, Lorblanchet M, Ramos-Muñoz J, Weniger G-Ch, Zilhão J. (c) Dates for Neanderthal art and symbolic behaviour are reliable. Nat Ecol Evol. 2018; 2 (7): 1044–1045. 10.1038/s41559-018-0598-z 29942018

[63] Hoffmann DL, Standish CD, García-Diez M, Pettitt PB, Milton JA, Zilhão J et al (d). Response to Comment on “U-Th dating of carbonate crusts reveals Neandertal origin of Iberian cave art”. Science 2018; 362 (6411): eaau1736 10.1126/science.aau1736 30309914

[64] Aubert M, Brumm A, Huntley J. Early dates for ‘Neanderthal cave art’ may be wrong. J Hum Evol. 2018; 125: 215–217. 10.1016/j.jhevol.2018.08.004 30173883

[65] Pearce DG, Bonneau A. Trouble on the dating scene. Nat Ecol Evol. 2018; 2 (6): 925 10.1038/s41559-018-0540-4 29632350

[66] Slimak L, Fietzke J, Geneste JM, Ontañón R. Comment on “U-Th dating of carbonate crusts reveals Neandertal origin of Iberian cave art”. Science 2018; 361 (6408): eaau1371 10.1126/science.aau1371 30237321

[67] Sankararaman S, Mallick S, Dannemann M, Prüfer K, Kelso J, Pääbo S, Patterson N, Reich D. The genomic landscape of Neanderthal ancestry in present-day human. Nature 2014; 507: 354–357. 10.1038/nature12961 24476815 PMC4072735

[68] Sankararaman S, Mallick S, Patterson N, Reich D, 2016 The combined landscape of Denisovan and Neanderthal ancestry in presentday humans. Curr Biol. 26: 1241–1247. 10.1016/j.cub.2016.03.037 27032491 PMC4864120

[69] Vernot B, Tucci S, Kelso J, Schraiber JG, Wolf AB, Gittelman RM, Dannemann M, Grote S, McCoy RC, Norton H. et al Excavating Neandertal and Denisovan DNA from the genomes of Melanesian individuals. Science 2016; 352 (6282): 235–239. 10.1126/science.aad9416 26989198 PMC6743480

[70] Gunz P, Tilot AK, Wittfeld K, Teumer A, Shapland CY, Van Erp TG. et al. Neandertal Introgression Sheds Light on Modern Human Endocranial Globularity. Curr Biol. 2019; 29 (1): 120–127. 10.1016/j.cub.2018.10.065 30554901 PMC6380688

[71] Quach H, Rotival M, Pothlichet J, Loh Y-HE, Dannemann M, Zidane N, Laval G, Patin E, Harmant C, Lopez M, et al Genetic adaptation and Neandertal admixture shaped the immune system of human populations. Cell 2016; 167: 643–656. 10.1016/j.cell.2016.09.024 27768888 PMC5075285

[72] Bodu P, Salomon H, Lacarrière J, Baillet M, Ballinger M, Naton H-G, Thery-Parisot I. Un gisement châtelperronien de plein air dans le Bassin parisien: les Bossats à Ormesson (Seine-et-Marne). Gallia Préhistoire 2014; 57 Available from: https://journals.openedition.org/galliap/478.

[73] Bonjean D, Vanbrabant Y, Abrams G, Pirson S, Burlet C, Di Modica K, Otte M, Auwera JV, Golitko M, McMillan R, Goemaere E. A new Cambrian black pigment used during the late Middle Palaeolithic discovered at Scladina Cave (Andenne, Belgium). J Archaeol Sci. 2015; 55: 253–265.

[74] Dayet L, Faivre J-Ph, Le Bourdonnec F-X, Discamps E, Royer A, Claud É, Lahaye C, Cantin N, Tartar E, Queffelec A, Gravina B, Turq A, d’Errico F. Manganese and iron oxide use at Combe-Grenal (Dordogne, France): A proxy for cultural change in Neanderthal communities. J Archaeol Sci Rep. 2019; 25: 239–256.

[75] Dayet L, d’Errico F, Garcia-Moreno R. Searching for consistencies in Châtelperronian pigment use. J Archaeol Sci. 2014; 44: 180–193.

[76] De Lumley H, Audubert F, Khatib S, Perrenoud C, Roussel B, Saos T, Szelewa A. Chapitre 44: Les crayons d’ocre du site aucheléen de Terra Amata In: De Lumley H, Terra Amata. Nice, Alpes-Maritimes, France. Tome V. Comportement et mode de vie des chasseurs acheuléens de Terra Amata. France: CNRS Editions; 2016 pp. 233–276.

[77] Mathis F, Bodu P, Dubreuil O, Salomon H. PIXE identification of the provenance of ferruginous rocks used by Neanderthals. Nucl Instrum Meth Phys Res B. 2014; 331: 275–279.

[78] Pitarch Martí A, d’Errico F. Seeking black. Geochemical characterization bu PIXE of Palaeolithic manganese-rich lumps and their potential sources. J Anthr Archaeol. 2018; 50: 54–68.

[79] Salomon H, Vignaud C, Coquinot Y, Beck L, Stringer C, Strivay D, d'Errico F. Selection and heating of colouring materials in the mousterian level of Es-Skhul (c. 100,000 years BP, Mount Carmel, Israel). Archaeometry 2012; 54 (4), 698–722.

[80] Bodu P, Salomon H, Leroyer M, Naton H-G, Lacarrière J, Dessoles M. An open-air site from the recent middle Palaeolithic in the Paris Basin (France): Les Bossats at Ormesson (Seine-et Marne). Quat Int. 2017; 331: 39–59.

[81] Stepanchuk VN. The Lower and Middle Paleolithic of Ukraine (Nizhnii i srednii paleolit Ukrainy). Chernovtsy: Zelena Bukovina; 2006. (In Russian).

[82] Stepanchuk VN, Vasilyev SV, Khaldeeva NI, Kharlamova NV, Borutskaya SB. The last Neanderthals of Eastern Europe: Micoquian layers IIIa and III of the site of Zaskalnaya VI (Kolosovskaya), anthropological records and context. Quat Int. 2015; 428: 132–150.

[83] Kuhn SL. Signaling theory and technologies of communication in the Paleolithic. Biol Theory 2014; 9 (1): 42–50.

[84] Mithen S. The Singing Neanderthals: the Origins of Music, Language, Mind and Body. London: Weidenfeld & Nicholson 2005.

[85] Soressi M, Rendu W, Texier J-P, Claud E, Daulny L, d'Errico F, Laroulandie V, Maureille B, Niclot M, Schwortz S, Tillier A-M. Pech-de-l'Azé I (Dordogne, France): nouveau regard sur un gisement moustérien de tradition acheuléenne connu depuis le 19ème siècle. Mém Soc Préhistorique Franç. 2008; 47: 95–132.

[86] Heyes PJ, Anastasakis K, de Jong W, van Hoesel A, Roebroeks W, Soressi M. Selection and Use of Manganese Dioxide by Neanderthals. Sci Rep. 2016; 6: 22159 10.1038/srep22159 26922901 PMC4770591

[87] Peyrony D. Le Moustier. Ses gisements, ses industries, ses couches géologiques. Revue Anthropologique 1930. Tome XL: 48–76 and 155–176.

[88] Laville H, Rigaud J-P. L’abri inférieur du Moustier (Dordogne). Précisions stratigraphiques et chronologiques. Compte Rendu de l’Académie des Sciences de Paris 1973; 276: 3097–3100.

[89] Valladas H, Geneste JM, Joron JL, Chadelle JP. Thermoluminescence dating of Le Moustier (Dordogne, France). Nature 1986; 322: 452–454.

[90] Gravina B, Discamps E. MTA-B or not to be? Recycled bifaces and shifting hunting strategies at Le Moustier and their implications for the late Middle Palaeolithic in southwestern France. J Hum Evol. 2015; 84: 83–98. 10.1016/j.jhevol.2015.04.005 25976251

[91] Gravina B. La fin du Paléolithique moyen en Poitou-Charentes et Périgord: considérations à partir de l'étude taphonomique et technoéconomique des sites du Moustier (niveaux G à K) et La Roche-à- Pierrot, Saint Césaire (niveau EJOP supérieur). Thesis, Université de Bordeaux, France. 2016.

[92] Gravina B. Intra-level technological change and its implications for Mousterian assemblage variability. The example of Le Moustier, layer G. Quat Int. 2017; 433: 132–139.

[93] Lemeur C. Variabilité des stratégies de subsistance au Moustérien final: l’exemple du Moustier (Dordogne). Unpublished M. Sc. Thesis, Université Toulouse Jean Jaurès. 2017.

[94] Thomas M. Taphonomie et techno-économie de palimpsestes d’occupation: l’exemple de la couche H du Moustier (Saint-Léon su Vézère, Dordogne). Unpublished M. Sc. Thesis, Université Toulouse Jean Jaurès, France. 2017.

[95] Faivre JP, Discamps E, Gravina B, Turq A, Bourguignon L. Cleaning up a Messy Mousterian: How to describe and interpret Late Middle Palaeolithic chrono-cultural variability in Atlantic Europe. Quat Int. 2017; 433: 1–3.

[96] San Juan C. Les matières colorantes dans les collections du Musée National de Préhistoire des Eyzies. Paléo 1990; 2: 229–242.

[97] Discamps E, Royer A. Reconstructing palaeoenvironmental conditions faced by Mousterian hunters during MIS 5 to 3 in southwestern France: a multi−scale approach using data from large and small mammal communities. Quat Int. 2017; 433: 64−87.

[98] Aujoulat N. Lascaux. Le rôle du déterminisme naturel: des modalités d’élection du site aux protocoles de conservation des édifices graphiques pariétaux. Thesis, Université de Bordeaux I, Bordeaux. 2002.

[99] Chalmin E. Caractérisation des oxydes de manganèse et usage des pigments noirs au Paléolithique Supérieur. Thesis, Université Marne-La-Vallée, Paris. 2003.

[100] Vandiver P. Palaeolithic Pigments and Processing. MSc dissertation. Massachusetts Institute of Technology, Massachusetts (U.S). 1983.

[101] Lucas-Tooth J, Price BJ. A Mathematical Method for the Investigation of Interelement Effects in X-Ray Fluorescence Analysis. Mettalurgia 1961; 64: 149–152.

[102] Castro K, Pérez-Alonso M, Rodríguez-Laso MD, Fernández LA, Madariaga JM. 2005 On-line FT-Raman and dispersive Raman spectra database of artists' materials (e-VISART database). Anal Bioanal Chem. 382: 248–258. 10.1007/s00216-005-3072-0 15729545

[103] Downs RT. The RRUFF Project: an integrated study of the chemistry, crystallography, Raman and infrared spectroscopy of minerals. Program and Abstracts of the 19th General Meeting of the International Mineralogical Association in Kobe, Japan. 2006. pp. 3–13.

[104] Julien CM, Massot M, Poinsignon C. 2004 Lattice vibrations of manganese oxides: Part I. Periodic structures. Spectrochim Acta A 60 (3): 689–700. 10.1016/s1386-1425(03)00279-8 14747095

[105] Sepúlveda M, Gutiérrez S, Vallette MC, Standen VG, Arriaza BT, Cárcamo-Vega JJ. 2015 Micro-Raman spectral identification of manganese oxides black pigments in an archaeological context in Northern Chile. Herit Sci. 3(1): 32

[106] Martin-Fernandez JA, Barcelo-Vidal C, Pawlowsky- Glahn V. Dealing with Zeros and Missing Values in Compositional Data Sets Using Nonparametric Imputation. Math Geol. 2003; 35: 253–278.

[107] Aitchison J. The Statistical Analysis of Compositional Data Monographs on Statistics and Applied Probability. London (UK): Chapman & Hall Ltd; 1986 p. 416.

[108] Dray S, Dufour AB. The ade4 package: implementing the duality diagram for ecologists. J Stat Softw. 2007; 22 (4): 1–20.

[109] Rosso DE, d’Errico F, Queffelec A. Patterns of change and continuity in ochre use during the late Middle Stone Age of the Horn of Africa: The Porc-Epic Cave record. PLoS ONE 2017; 12 (5): e0177298 10.1371/journal.pone.0177298 28542305 PMC5443497

[110] Power C, Sommer V, Watts I. The Seasonality Thermostat: Female Reproductive Synchrony and Male Behavior in Monkeys, Neanderthals, and Modern Humans. Paleo Anthr. 2013: 33–60.

[111] Rifkin RF, Dayet L, Queffelec A, Summers B, Lategan M, d’Errico F. Evaluating the Photoprotective Effects of Ochre on Human Skin by In Vivo SPF Assessment: Implications for Human Evolution, Adaptation and Dispersal. PLoS ONE 2015; 10 (9): e013609. 10.1371/journal.pone.0136090 PMC4564224 26353012

[112] Sonneville-Bordes D. Manganèse raclé dans le Moustérien type Ferrassie de Caminade Est (Dordogne). Quaternaria 1969; XI: 111–114.

