# Supplementary material for: Provenance, modification and use of manganese-rich rocks at Le Moustier (Dordogne, France)
Source: PLoS One. 2019 Jul 17;14(7):e0218568. doi: 10.1371/journal.pone.0218568 (PMC6636720; doi:10.1371/journal.pone.0218568)
Supplement: S1 Table — (PDF) [file pone.0218568.s001.pdf]

Provenance, modification and use of manganese-rich rocks at Le Moustier (Dordogne, France)

Africa Pitarch Martí*, Francesco d'Errico, Alain Turq, Eric Lebraud, Emmanuel Discamps, Brad Gravina

*Corresponding Author:

E-mail: africa.pitarch@gmail.com (APM)

S1 Tables

Supplementary data on the EDXRF calibration, the archaeological materials and the geological samples.

Table A. Corrections used for the EDXRF calibration.

Table B. Elemental composition of certified reference materials and self-produced standards employed to calibrate the EDXRF spectrometer.

Table C. Calibrated elemental composition of the analysed standards.

Table D. Descriptive data on modified and unmodified Mn-rich black lumps from Le Moustier.

Table E. Elemental composition of archaeological Mn-rich black lumps from Le Moustier obtained by EDXRF.

Table F. Coefficient of variation of elements composing the Mn-rich black lumps from Le Moustier.

Table G. Results of SEM-EDS analyses of Mn-rich lumps from Le Moustier.

Table H. Processing techniques, degree of modification, facet morphology, and striations main orientation of Mn-rich black lumps from Le Moustier.

Table I. Results of EDXRF analysis of geological samples.

Table J. Results of SEM-EDS analyses of the geological samples.

Table A. Corrections used for the EDXRF calibration

Calib. Element	Overlap. Elements Corr.	Matrix Elements Corr.	R^2	N. Stds used	Stds omitted from calib. (*)
Si	P, Sr	Si, Ca, Mn, Fe	1.00	12	-
K	Ca	Mn	1.00	11	STD-05
Ca	-	-	1.00	11	NCS DC 11020
Ti	Ba	Mn	0.98	7	NCS DC 28043; STD-01
Mn	Fe	Si, Ca, Mn, Fe	0.99	11	STD-03
Fe	Mn, Co	Si, Ca, Mn, Fe	1.00	11	NCS DC 11023
Ni	Co, Cu, W	Si, Ca, Mn, Fe	0.99	11	-
Zn	Cu	-	0.98	9	NCS DC 28043; NCS DC 11020
As	Pb	Si, Ca, Mn	1.00	9	STD-05
Sr	-	-	1.00	6	STD-07
Ba	-	Si, Ca, Mn, Fe	0.99	11	STD-06
Pb	As		1.00	9	STD-04

Calib.: calibration; Overlap.: ovelapping; Corr. correction; Stds: standards.

Table B. Elemental composition of certified reference materials and self-produced standards employed to calibrate the EDXRF spectrometer

Reference	Si		K		Ca		Ti		Mn		Fe		Ni		Zn		As		Sr		Pb		Ba	
	Conc.	Unc.	Conc.	Unc.	Conc.	Unc.	Conc.	Unc.	Conc.	Unc.	Conc.	Unc.	Conc.	Unc.	Conc.	Unc.	Conc.	Unc.	Conc.	Unc.	Conc.	Unc.	Conc.	Unc.
NCS DC 11023	6.1	0.02	0.33	0.00	1.67	0.01	0.086	0.001	35.54	0.1	10.3	0.1	0.023	0.002	0.066	0.003	0.112	0.003	-	-	0.058	0.003	1.45	0.04
NCS DC 11020	13.1	0.05	0.92	0.01	1.69	0.03	0.162	0.002	22.31	0.1	9.7	0.0	0.089	0.003	0.170	0.01	0.034	0.001	-	-	0.120	0.003	0.48	0.01
NCS DC 28041	26.2	0.04	3.1	0.04	1.48	0.02	0.106	0.003	14.45	0.1	0.85	0.01	-	-	0.015	0.002	0.013	0.002	-	-	0.003	0.0004	0.06	0.003
NCS DC 28043	8.1	0.03	0.54	0.02	0.82	0.01	0.129	0.003	30.99	0.1	10.7	0.1	0.083	0.002	0.235	0.01	0.089	0.002	-	-	0.110	0.005	0.99	0.01
NCS DC 47004	7.6	0.04	0.83	0.02	0.76	0.02	0.038	0.002	45.39	0.1	1.22	0.02	0.019	0.001	0.027	0.002	-	-	-	-	-	-	0.61	0.04
STD-01	3.6	0.18	0.14	0.01	12.6	0.3	0.029	0.007	35.5	0.4	1.17	0.02	0.055	0.003	0.185	0.02	0.028	0.001	0.014	0.001	0.001	0.0001	2.89	0.1
STD-02	20.5	0.21	0.84	0.04	6.6	0.1	0.126	0.013	13.7	0.1	1.16	0.02	0.013	0.001	0.474	0.047	0.003	0.0003	0.008	0.0004	0.001	0.0001	0.06	0.0028
STD-03	2.99	0.15	0.20	0.02	5.9	0.1	0.016	0.004	47.6	0.5	0.14	0.01	0.014	0.001	<LOD	-	0.001	0.0001	0.004	0.0002	0.000	0.0000	0.92	0.05
STD-04	7.7	0.08	1.46	0.07	1.37	0.1	0.113	0.011	41.6	0.4	2.55	0.1	0.021	0.001	0.051	0.005	0.035	0.002	0.487	0.024	0.011	0.001	2.63	0.1
STD-05	0.47	0.02	0.06	0.01	0.39	0.02	<LOD	-	62.4	0.6	0.08	0.00	0.023	0.001	0.034	0.003	0.081	0.004	0.033	0.002	0.000	0.0000	0.27	0.01
STD-06	0.80	0.04	0.06	0.01	27.6	0.6	<LOD	-	14.7	0.1	0.27	0.01	0.016	0.001	0.044	0.004	0.004	0.0004	0.008	0.0004	0.001	0.0001	3.11	0.2
STD-07	2.57	0.13	0.09	0.01	31.2	0.6	<LOD	-	8.2	0.1	0.78	0.02	0.015	0.001	0.007	0.001	0.007	0.0003	0.017	0.001	0.001	0.0001	1.42	0.1

Values expressed in percentages. NCS DC: certified reference material; STD: self-produced standard. Conc.: concentration; Unc.: uncertainty.

Table C. Calibrated elemental composition of the analysed standards

Reference	Si		K		Ca		Ti		Mn		Fe		Ni		Zn		As		Sr		Pb		Ba	
	Conc.	Std	Conc.	Std	Conc.	Std	Conc.	Std	Conc.	Std	Conc.	Std	Conc.	Std	Conc.	Std	Conc.	Std	Conc.	Std	Conc.	Std	Conc.	Std
NCS DC 11023	6.5	0.4	0.37	0.01	1.97	0.005	0.091	0.005	34.4	0.7	9.7	0.1	0.021	0.001	0.058	0.001	0.112	0.003	0.099	0.001	0.044	0.003	1.44	0.02
NCS DC 11020	13.7	0.1	0.99	0.01	1.53	0.03	0.155	0.004	22.5	0.2	9.4	0.1	0.088	0.001	0.139	0.002	0.034	0.001	0.070	0.002	0.120	0.002	0.47	0.02
NCS DC 28041	25.8	0.4	2.94	0.04	1.23	0.05	0.111	0.003	14.7	0.1	0.9	0.01	<LOD	-	0.033	0.005	0.013	0.0004	0.686	0.003	0.003	0.001	0.062	0.013
NCS DC 28043	7.9	0.3	0.59	0.01	0.86	0.03	0.110	0.008	31.6	0.5	10.7	0.1	0.085	0.002	0.170	0.003	0.093	0.003	0.098	0.002	0.103	0.003	1.02	0.06
NCS DC 47004	7.8	0.6	0.84	0.01	0.85	0.01	0.024	0.005	45.4	1.3	1.13	0.02	0.019	0.001	0.039	0.001	0.010	0.001	0.156	0.002	0.004	0.001	0.65	0.03
STD-01	4.8	1.0	0.11	0.01	10.2	0.1	0.146	0.059	36.7	0.4	0.78	0.01	0.054	0.001	0.132	0.001	0.030	0.001	0.017	0.001	<LOD	-	2.61	0.35
STD-02	19.9	0.7	0.85	0.03	7.1	0.02	0.125	0.001	20.0	0.3	1.09	0.04	0.016	0.001	0.507	0.003	0.003	0.001	0.015	0.002	0.004	0.001	0.050	0.004
STD-03	3.3	0.7	0.17	0.01	6.7	0.03	0.021	0.001	50.3	0.9	0.22	0.01	0.013	0.001	0.022	0.001	0.001	0.0001	0.001	0.0005	0.003	0.001	1.07	0.11
STD-04	<LOD	-	0.06	0.01	0.51	0.01	0.050	0.004	61.5	0.5	0.08	0.02	0.026	0.002	0.042	0.001	0.081	0.001	0.028	0.002	0.015	0.002	0.32	0.043
STD-05	8.1	0.8	1.09	0.03	1.38	0.01	0.105	0.007	37.9	0.7	2.82	0.02	0.025	0.001	0.049	0.001	0.074	0.003	0.486	0.001	0.011	0.001	2.57	0.06
STD-06	2.84	0.24	0.02	0.004	27.3	0.1	0.140	0.009	13.7	0.1	0.24	0.04	0.016	0.001	0.033	0.003	0.002	0.0004	0.010	0.001	0.001	0.001	2.52	0.03
STD-07	3.5	0.8	0.15	0.02	31.8	0.1	0.053	0.008	7.6	0.1	0.86	0.01	0.017	0.000	0.020	0.001	0.006	0.0003	0.070	0.002	<LOD	-	1.41	0.03

Values expressed as percentage. Element contents were calculated as the average of five acquisitions. NCS DC: certified reference material; STD: self-produced standard.

Table D. Descriptive data on modified and unmodified Mn-rich black lumps from Le Moustier

Piece	Length (mm)	Width (mm)	Thickness (mm)	Weight (g)	Density	Hardness	Type of lump / fragment	Morphology	Hue	Appearance	Rock structure	Anthrop. Mod.
MOU-MNP-01	55.50	38.00	38.30	110.30	Very high	Hard	Nodule	Square base pseudo-pyramidal	Black	Irregular dull surface	Massive, porous	Yes
MOU-MNP-02	48.50	33.00	18.40	36.50	High	Hard	NA	Prismatic	Anthracite	Irregular dull surface	Massive	Yes
MOU-MNP-03	37.80	26.90	21.10	34.90	Very high	Hard	Nodule	Irregular	Anthracite	Irregular dull surface	Massive	Yes
MOU-MNP-04	35.90	26.00	14.40	15.60	Medium	Hard	Nodule	Irregular	Anthracite	Irregular surface with a metallic sheen	Massive	Yes
MOU-MNP-05	32.80	22.70	15.20	11.40	Medium	Hard	Crust	Irregular	Black	Irregular dull surface	Granular, porous	Yes
MOU-MNP-06	27.90	20.90	16.50	3.10	Very low	Soft	NA	Irregular	Brownish black	Irregular dull surface	Massive	Possible
MOU-MNP-07	35.70	28.70	22.70	11.90	Medium	Hard	Crust	Pseudo-pyramidal	Very dark grey	Irregular dull surface	Massive, porous	Yes
MOU-MNP-08	58.10	45.20	26.00	56.20	Medium	Hard	Crust	Irregular	Very dark grey	Botryoidal dull surface	Massive, porous	Yes
MOU-MNP-09	46.70	44.60	27.90	47.00	High	Hard	Crust	Irregular	Very dark grey	Botryoidal surface with a metallic sheen	Massive, porous	No
MOU-MNP-10	43.50	28.10	19.60	24.90	High	Hard	Crust	Irregular	Anthracite	Botryoidal dull surface	Massive	Yes
MOU-MNP-11	34.70	26.40	13.80	13.20	Medium	Hard	Crust ?	Irregular	Very dark grey	Ribbed dull surface	Columnar	No
MOU-MNP-12	36.50	34.50	17.30	20.60	High	Hard	Crust	Irregular	Anthracite	Irregular dull surface, occasionnally botryoidal	Massive, slightly porous	Yes
MOU-MNP-13	29.90	26.30	14.90	16.40	High	Hard	Crust	Irregular	Anthracite	Irregular dull surface	Massive, slightly porous	Yes
MOU-MNP-14	20.40	17.40	11.70	4.60	Low	Hard	Crust	Irregular	Anthracite	Irregular dull surface, with local metallic sheen	Massive, porous	No
MOU-MNP-15	25.40	13.30	10.30	4.60	Medium	Hard	NA	Pyramidal	Very dark grey	Irregular dull surface, with local metallic sheen	Massive	Yes
MOU-MNP-16	16.80	13.80	12.20	2.90	Low	Hard	NA	Irregular	Very dark grey	Irregular dull surface, occasionnally glossy	Massive, slightly porous	Yes
MOU-MNP-17	17.10	15.80	12.40	3.00	Very low	Hard	NA	Irregular	Very dark grey	Irregular dull surface, with local metallic sheen	Massive, slightly porous	Possible
MOU-MNP-18	16.50	11.20	8.60	1.30	Very low	Hard	NA	Irregular	Very dark grey	Irregular dull surface, with local metallic sheen	Massive, slightly porous	No
MOU-MNP-19	26.03	19.37	17.93	13.50	Medium	Hard	NA	Irregular	Very dark grey	Irregular glossy surface	Massive, porous	Yes
MOU-MNP-20	15.27	13.73	7.28	1.30	Very low	Hard	NA	Irregular	Very dark grey	Irregular glossy surface	Massive	Possible
MOU-MNP-L&R-H7	23.21	19.32	10.74	5.82	Medium	Hard	NA	Pyramidal	Very dark grey	Irregular dull surface	Massive	Yes
MOU-MNP-L&R-H8	12.20	11.52	7.61	1.81	Medium	Hard	NA	Pyramidal	Very dark grey	NA	Massive	Yes
MOU-G&D-5983	6.88	4.19	3.48	0.18	Low		NA	NA	Dark grey	Irregular dull surface	Massive	No
MOU-G&D-6846-a	12.81	8.30	5.20	0.77	Low	Soft	NA	Pyramidalal	Very dark grey	Irregular dull surface	Massive, slightly porous	Yes
MOU-G&D-6846-b	9.73	5.82	3.56	0.38	Low	Soft	NA	Irregular	Very dark grey	Irregular dull surface	Massive, slightly porous	Yes
MOU-G&D-6846-c	7.02	6.50	4.16	0.27	Low	Soft	NA	Irregular	Very dark grey	Irregular dull surface	Massive, slightly porous	Yes
MOU-G&D-6857	27.26	15.70	12.30	7.79	Medium	Medium	NA	Pyramidal	Very dark grey	NA	Massive, slightly porous	Yes
MOU-G&D-7527-a	15.15	10.31	7.29	1.83	Low	Hard	NA	Pyramidal	Very dark grey	Irregular dull surface	Massive, slightly porous	Yes
MOU-G&D-7527-b	9.11	8.31	9.51	1.04	Low	Hard	NA	Irregular	Very dark grey	Irregular dull surface	Massive, slightly porous	Yes
MOU-G&D-4584-a1	13.08	7.89	5.25	0.76	Low	Hard	NA	Irregular	Very dark grey	Irregular dull surface	Massive, slightly porous	No
MOU-G&D-4584-a2	7.97	7.45	4.64	0.06	Low	Hard	NA	Irregular	Very dark grey	Irregular dull surface	Massive, slightly porous	No
MOU-G&D-4584-a3	7.29	6.02	3.78	0.19	Low	Hard	NA	Irregular	Very dark grey	Irregular dull surface	Massive, slightly porous	Possible
MOU-G&D-4584-a4	7.54	4.16	3.18	0.36	Low	Hard	NA	Irregular	Very dark grey	Irregular dull surface	Massive, slightly porous	Possible
MOU-G&D-4584-a5	7.10	4.85	2.55	0.12	Low	Hard	NA	Irregular	Very dark grey	Irregular dull surface	Massive, slightly porous	Possible
MOU-G&D-4584-a6	6.79	5.37	4.49	0.15	Low	Hard	NA	Irregular	Very dark grey	Irregular dull surface	Massive, slightly porous	Possible
MOU-G&D-5255-a1	11.23	10.09	6.59	0.92	Low	Hard	NA	Irregular	Very dark grey	Irregular dull surface	Massive	No
MOU-G&D-5255-a2	7.20	5.79	3.03	0.17	Low	Hard	NA	Irregular	Very dark grey	Irregular dull surface	Massive	No
MOU-G&D-5902-a2	13.49	8.64	4.54	0.98	Low	Medium	NA	Irregular	Very dark grey	Irregular dull surface	Massive	No
MOU-G&D-5902-a3	12.50	7.39	5.61	0.92	Low	Hard	NA	Irregular	Very dark grey	Irregular dull surface, with local blueish reflexion	Massive	No
MOU-G&D-5902-a4	9.75	7.35	6.56	0.62	Low	Hard	NA	Irregular	Very dark grey	Irregular dull surface	Massive	No
MOU-G&D-5902-a5	8.67	7.26	3.23	0.11	Low	Hard	NA	Irregular	Very dark grey	Irregular dull surface	Massive	No
MOU-G&D-9796	26.13	14.12	15.11	4.28	Low	Hard	NA	Irregular	Very dark grey	Irregular dull surface	Massive	Yes
MOU-G&D-11571	14.77	13.11	9.64	3.76	Medium	Hard	NA	Irregular	Very dark grey	Irregular dull surface	Massive	Yes
MOU-G&D-8975	12.58	11.21	5.57	0.71	Low	Hard	NA	Irregular	Very dark grey	Irregular dull surface, with local metallic sheen	Slightly laminated; with some crystals	Yes
MOU-G&D-9858-a	10.47	9.33	6.99	1.12	Low	Hard	NA	Irregular	Very dark grey	Irregular glossy surface	Massive	Yes
MOU-G&D-9858-b	16.88	12.49	9.79	2.17	Very low	Hard	NA	Irregular	Dark grey	Botryoidal dull surface	Massive	No
MOU-G&D-9858-c	12.58	5.69	2.98	0.17	Low	Hard	NA	Irregular	Dark grey	Botryoidal dull surface	Massive	No
MOU-G&D-9858-d	7.17	5.72	2.98	0.09	Low	Hard	NA	Irregular	Dark grey	Botryoidal dull surface	Massive	No
MOU-G&D-11869	16.67	8.42	9.08	2.38	High	Hard	NA	Pseudo-ellipsoidal	Very dark grey	Irregular dull surface	Massive	Yes

Piece	Length (mm)	Width (mm)	Thickness (mm)	Weight (g)	Density	Hardness	Type of lump / fragment	Morphology	Hue	Appearance	Rock structure	Anthrop. Mod.
MOU-G&D-11236-a	8.68	6.58	6.36	0.58	Low	Hard	NA	Irregular	Very dark grey	Irregular dull surface	Massive, slightly porous	No
MOU-G&D-11236-b	14.67	7.67	5.04	0.45	Very low	Hard	NA	Irregular	Very dark grey	Irregular dull surface	Massive	Yes
MOU-G&D-12575-a	11.57	9.18	8.46	1.16	Medium	Hard	NA	Irregular	Very dark grey	Irregular dull surface	Massive	Yes
MOU-G&D-12575-b	8.46	6.63	4.15	0.24	Low	Hard	NA	Irregular	Very dark grey	Irregular dull surface	Massive	No
MOU-G&D-13440	8.66	8.10	3.39	0.20	Low	Hard	Crust	Cubic	Very dark grey	Irregular dull surface	Massive	No

Anthrop.Mod.: Anthropogenic modifications

Table E. Elemental composition of archaeological Mn-rich black lumps from Le Moustier obtained by EDXRF

Piece	Si	K	Ca	Ti	Mn	Fe	Ni	Zn	As	Sr	Ba	Pb
MOU-MNP-01_01	6.1	0.25	1.2	0.912	36.4	*0.004*	*5*	341	385	1169	57440	*10*
MOU-MNP-01_02	5.4	0.25	2.2	0.927	33.4	*0.004*	6	322	352	1128	55340	*9*
MOU-MNP-01_03	3.0	0.22	1.6	1.049	33.5	*0.004*	*4*	294	272	717	52040	*9*
MOU-MNP-01_04	4.7	0.19	1.3	0.973	35.7	*0.004*	*4*	292	309	974	55990	*9*
MOU-MNP-02_01	*2.4*	0.19	2.5	0.076	50.0	*0.006*	265	865	149	284	35420	85
MOU-MNP-02_02	8.7	0.29	6.1	0.220	36.3	0.04	207	1938	101	353	30720	50
MOU-MNP-02_03	8.1	0.34	7.3	0.223	35.1	*0.005*	204	547	143	340	28030	*11*
MOU-MNP-02_04	5.6	0.25	2.5	0.318	41.0	0.18	261	3165	106	260	28840	27
MOU-MNP-03_01	8.4	0.14	2.2	0.090	43.4	0.82	94	484	197	326	26780	*12*
MOU-MNP-03_02	9.8	0.16	2.9	0.134	40.3	0.80	100	468	170	354	23940	18
MOU-MNP-04_01	2.8	0.10	1.5	0.069	56.4	0.21	1564	501	468	247	2217	51
MOU-MNP-04_02	3.8	0.10	1.2	0.072	55.2	0.17	1610	519	467	262	1843	48
MOU-MNP-05_01	24.5	0.92	2.2	0.345	21.3	0.98	9	319	62	206	19030	31
MOU-MNP-05_02	25.1	0.91	2.1	0.316	21.2	0.95	14	303	65	211	20080	20
MOU-MNP-06_01	6.0	0.89	0.5	0.294	42.1	0.44	1207	1817	109	2346	25160	*11*
MOU-MNP-06_02	9.6	0.97	0.6	0.297	40.6	0.48	1311	1892	111	2196	22020	*11*
MOU-MNP-06_03	*1.9*	0.64	0.4	0.283	47.5	0.17	1480	2019	119	1877	19650	*11*
MOU-MNP-06_04	6.4	0.73	0.4	0.410	35.6	0.42	1024	1612	98	1424	11350	*8*
MOU-MNP-07_01	11.9	0.47	2.3	0.414	32.4	0.54	238	313	43	218	33900	24
MOU-MNP-07_02	11.3	0.41	1.6	0.387	36.3	0.46	294	325	51	214	36050	19
MOU-MNP-07_03	9.1	0.37	1.6	0.388	36.6	0.46	289	313	59	190	36000	*9*
MOU-MNP-07_04	13.4	0.52	1.8	0.359	35.4	0.64	300	335	56	262	37050	26
MOU-MNP-07_05	12.8	0.52	1.8	0.367	35.4	0.67	301	354	46	260	36200	34
MOU-MNP-08_01	15.9	0.44	1.5	0.085	35.5	0.49	86	344	145	121	766	*11*
MOU-MNP-08_02	16.7	0.51	1.8	0.084	33.5	0.56	88	350	133	104	1052	*11*
MOU-MNP-09_01	8.4	0.35	1.2	0.016	46.0	0.51	158	415	261	76	9762	114
MOU-MNP-09_02	5.5	0.28	1.7	0.019	50.4	0.45	208	448	296	85	9483	73
MOU-MNP-09_03	8.3	0.42	1.6	0.115	44.1	0.37	182	375	123	716	13950	*11*
MOU-MNP-09_04	6.8	0.38	1.2	0.139	45.4	0.24	164	361	126	761	13730	*11*
MOU-MNP-10_01	2.9	0.32	0.4	*0.0037*	55.4	0.71	71	305	473	431	49780	350
MOU-MNP-10_02	4.6	0.27	0.4	*0.0038*	54.1	0.64	65	316	496	434	50770	362
MOU-MNP-11_01	15.7	0.60	1.1	0.465	31.2	1.21	141	292	74	426	53700	834
MOU-MNP-11_02	13.2	0.51	1.0	0.218	34.8	1.00	170	275	91	289	49470	586
MOU-MNP-12_01	4.3	0.21	0.6	0.031	54.3	0.53	170	279	190	376	6786	*15*
MOU-MNP-12_02	5.7	0.25	0.6	0.027	52.1	0.58	174	275	148	397	7250	35
MOU-MNP-13_01	12.7	0.40	1.2	0.064	39.7	0.66	84	357	309	188	6570	21
MOU-MNP-13_02	11.9	0.36	1.1	0.051	38.9	0.84	103	363	352	208	6750	34
MOU-MNP-14_01	11.1	0.42	0.5	0.075	42.9	0.49	91	242	103	567	5557	*12*
MOU-MNP-14_02	8.9	0.42	0.5	0.064	46.4	0.48	113	273	111	578	5877	*13*
MOU-MNP-15_01	9.4	0.61	1.8	0.199	39.0	0.87	519	522	169	2140	24170	*11*
MOU-MNP-15_02	8.9	0.59	1.6	0.222	40.1	0.83	515	535	162	2438	25880	*11*
MOU-MNP-16_01	11.6	0.39	1.6	0.066	40.7	0.50	132	336	259	148	3565	49
MOU-MNP-16_02	11.8	0.32	1.3	0.067	40.9	0.41	123	333	275	154	3098	39
MOU-MNP-17_01	13.7	0.41	1.8	0.082	36.3	0.51	63	290	139	83	5355	*1*
MOU-MNP-17_02	16.2	0.45	2.0	0.020	33.8	0.55	77	280	149	92	15580	*11*
MOU-MNP-17_03	16.0	0.42	1.6	0.057	33.6	0.59	60	337	145	97	9727	*10*
MOU-MNP-17_04	17.0	0.58	3.6	0.089	29.9	0.75	76	318	147	99	3716	*10*
MOU-MNP-17_05	15.2	0.62	3.1	0.080	33.2	0.76	82	306	179	108	4058	*12*
MOU-MNP-17_06	16.6	0.59	3.9	0.088	29.6	0.78	63	325	146	105	3348	*10*
MOU-MNP-18_01	12.0	0.57	2.3	0.088	38.9	0.81	107	241	111	790	6391	*13*
MOU-MNP-18_02	10.6	0.54	2.1	0.091	41.1	0.77	121	254	103	772	6095	*12*
MOU-MNP-18_03	13.1	0.50	1.5	0.088	39.0	0.58	86	278	89	490	4203	17
MOU-MNP-18_04	13.2	0.49	1.4	0.090	38.8	0.56	81	278	94	469	4514	*11*
MOU-MNP-19_01	7.6	0.43	1.3	0.062	44.8	0.56	579	303	182	93	3048	33
MOU-MNP-19_02	15.4	0.75	1.9	0.155	34.5	0.82	402	320	95	512	25540	*11*
MOU-MNP-19_03	18.4	0.79	1.7	0.626	28.3	0.90	350	304	65	818	30280	*9*
MOU-MNP-20_01	17.3	1.18	1.1	0.375	31.6	0.87	714	423	173	953	34010	*10*
MOU-MNP-20_02	16.2	1.13	1.0	0.412	32.1	0.76	679	405	170	956	34640	*10*
MOU-MNP-20_03	11.3	1.11	1.1	0.768	33.8	*0.00*	497	367	149	2415	42380	*10*
MOU-G&D-9796_01	17.2	0.81	4.1	0.596	24.6	0.71	82	354	75	2811	49210	*10*
MOU-G&D-9796_02	15.9	0.71	3.0	0.484	30.5	0.59	177	375	111	2143	46680	*10*
MOU-G&D-9796_03	17.0	0.72	3.2	0.477	29.5	0.61	198	407	105	2340	46170	*10*
MOU-G&D-11571_01	12.2	0.41	1.5	0.081	40.6	0.59	104	336	127	104	1069	25

Piece	Si	K	Ca	Ti	Mn	Fe	Ni	Zn	As	Sr	Ba	Pb
MOU-G&D-11571_02	13.4	0.63	2.6	0.082	34.4	0.84	131	338	148	122	1559	27
MOU-G&D-11571_03	15.6	0.48	1.3	0.082	35.2	0.67	85	310	107	88	816	*12*
MOU-G&D-7527-a_01	19.2	0.37	1.9	0.142	27.1	0.50	28	301	162	150	14410	*11*
MOU-G&D-7527-a_02	16.2	0.54	1.1	0.156	35.7	0.43	42	302	188	163	17710	*11*
MOU-G&D-7527-b_01	15.4	0.29	1.1	0.065	34.5	0.44	85	302	189	135	4923	13
MOU-G&D-7527-b_02	17.8	0.25	0.9	0.050	33.5	0.44	57	282	159	134	6801	*11*
MOU-G&D-7527-b_03	17.2	0.28	0.9	0.048	32.1	0.45	56	277	180	110	6929	*1*
MOU-G&D-6857_01	17.3	0.53	2.5	0.092	28.4	0.67	56	330	123	153	3207	*12*
MOU-G&D-6857_02	16.6	0.59	2.0	0.086	30.1	0.65	73	344	140	145	2251	*12*
MOU-MNP-L&R-H7_01	6.1	0.18	0.4	0.043	51.2	0.13	123	326	798	196	9180	*13*
MOU-MNP-L&R-H7_02	10.5	0.19	0.6	*0.0036*	44.2	0.05	137	348	758	348	13400	25
MOU-MNP-L&R-H7_03	9.4	0.23	0.6	0.074	46.4	0.32	126	332	690	207	7922	*12*
MOU-MNP-L&R-H8_01	5.2	0.27	0.7	0.004	53.5	0.21	470	377	306	427	7279	37
MOU-MNP-L&R-H8_02	6.0	0.44	1.1	*0.0037*	50.9	0.28	468	373	245	854	11710	61
MOU-MNP-L&R-H8_03	9.0	0.47	1.1	0.034	46.1	0.49	354	348	225	604	8139	19
MOU-G&D-4584a1_01	4.7	0.34	1.1	0.033	51.5	0.45	512	417	178	723	17300	*13*
MOU-G&D-4584a1_02	6.0	0.30	1.1	0.133	48.6	0.39	438	384	157	637	15520	*12*
MOU-G&D-4584a1_03	7.5	0.43	1.3	0.049	48.4	0.54	740	461	133	182	4799	41
MOU-G&D-5255a1_01	9.3	0.45	1.0	*0.004*	45.5	*0.01*	268	390	481	984	18510	16
MOU-G&D-5255a1_02	6.6	0.40	1.0	*0.004*	49.7	*0.01*	277	406	492	967	18340	30
MOU-G&D-5255a1_03	8.5	0.38	1.0	*0.004*	47.0	*0.01*	271	408	493	871	17030	32
MOU-G&D-5902a2_01	13.7	0.83	1.6	0.246	34.5	1.72	257	459	230	966	18460	14
MOU-G&D-5902a2_02	15.4	0.83	1.6	0.250	31.9	1.69	207	432	199	865	16760	*11*
MOU-G&D-5902a2_03	15.9	0.82	1.5	0.308	31.2	1.66	193	433	202	938	17510	*11*
MOU-G&D-6846a_01	11.3	0.37	1.5	0.078	42.5	0.37	104	317	233	171	1369	*13*
MOU-G&D-6846a_02	14.6	0.34	1.9	0.085	36.5	0.61	67	319	186	133	1433	*11*
MOU-G&D-6846a_03	11.9	0.27	1.5	0.081	39.9	0.47	82	277	168	103	887	*11*
MOU-G&D-8975_01	19.2	0.68	2.4	0.754	22.1	0.96	27	254	55	1322	33320	232
MOU-G&D-8975_02	20.1	0.65	2.3	0.756	21.6	0.99	16	259	52	1291	31260	237
MOU-G&D-8975_03	24.8	0.74	1.3	0.667	18.6	0.98	5	265	42	1287	30480	227
MOU-G&D-9858a_01	6.7	0.27	1.6	0.015	48.5	0.39	198	300	213	180	7330	53
MOU-G&D-9858a_02	6.4	0.30	1.5	*0.0036*	50.2	0.39	198	294	205	217	10770	40
MOU-G&D-9858a_03	5.3	0.21	1.2	0.033	52.7	0.31	201	322	196	193	5067	47
MOU-G&D-9858a_04	6.6	0.27	1.5	0.015	48.9	0.36	217	318	200	193	7190	72
MOU-G&D-9858b_01	11.0	0.43	2.2	0.527	34.0	0.38	201	347	102	1383	26300	*9*
MOU-G&D-9858b_02	9.4	0.40	2.4	0.397	38.5	0.37	270	366	124	1495	27490	*10*
MOU-G&D-9858b_03	10.8	0.50	2.3	0.288	36.9	0.84	295	458	152	2189	30190	*11*
MOU-G&D-9858b_04	9.8	0.41	2.4	0.249	37.1	0.75	268	427	143	1974	27640	*10*
MOU-G&D-11869_01	8.7	0.39	2.5	0.078	44.4	0.55	164	357	191	137	1339	30
MOU-G&D-11869_02	8.6	0.37	2.3	0.080	45.3	0.49	141	342	180	128	1176	15
MOU-G&D-11869_03	7.4	0.25	1.3	0.037	48.1	0.79	149	336	207	153	7736	152
MOU-G&D-11236b_01	*2.1*	0.22	1.1	0.944	36.3	0.55	241	415	114	3334	39320	*9*
MOU-G&D-11236b_02	3.2	0.28	1.3	0.916	38.9	0.68	278	500	131	4018	41640	*10*
MOU-G&D-11236b_03	4.4	0.32	2.6	0.909	34.0	*0.00*	224	434	116	3304	36820	*9*
MOU-G&D-12575a_01	9.9	0.38	2.6	0.080	42.1	0.58	113	252	228	139	1137	*14*
MOU-G&D-12575a_02	10.4	0.25	1.3	0.080	43.5	0.40	92	266	166	108	1010	*12*
MOU-G&D-12575a_03	12.7	0.48	2.4	0.086	38.1	0.68	117	247	177	125	1056	*12*
MOU-G&D-13440_01	8.7	0.56	2.3	0.512	38.7	0.13	99	314	81	193	34990	*10*
MOU-G&D-13440_02	8.9	0.48	2.0	0.687	35.7	0.84	80	315	107	135	36020	*10*
MOU-G&D-13440_03	9.6	0.48	2.0	0.719	35.2	0.83	71	319	107	139	34110	*9*

Major and minor elements are given in mass percentages, trace elements in parts per million; figures in italics indicate concentrations below the limit of detection calculated multiplying the equipment detection limit by 0.6 [106].

Table F. Coefficient of variation of elements composing the Mn-rich black lumps from Le Moustier

Piece	Si	K	Ca	Ti	Mn	Fe	Ni	Zn	As	Sr	Ba	Pb
MOU-MNP-01	28	14	27	6	4	4	37	8	15	21	4	6
MOU-MNP-02	54	24	54	48	15	146	13	73	19	15	9	78
MOU-MNP-03	11	10	20	28	5	2	4	2	10	6	8	55
MOU-MNP-04	21	2	14	2	1	13	2	2	0	4	13	4
MOU-MNP-05	2	1	6	6	0	3	27	3	3	2	4	30
MOU-MNP-06	59	20	20	19	11	37	14	8	7	21	30	14
MOU-MNP-07	14	14	16	6	5	18	9	5	13	14	3	47
MOU-MNP-08	3	10	12	1	4	10	1	1	6	11	22	0
MOU-MNP-09	19	17	17	89	6	30	13	10	45	93	21	105
MOU-MNP-10	33	10	10	2	2	7	7	2	3	0	1	2
MOU-MNP-11	12	12	4	51	8	13	13	4	14	27	6	25
MOU-MNP-12	21	9	0	10	3	7	2	1	18	4	5	80
MOU-MNP-13	5	8	4	15	1	18	14	1	9	7	2	33
MOU-MNP-14	16	2	7	11	5	1	15	9	6	1	4	6
MOU-MNP-15	4	3	10	8	2	3	0	2	3	9	5	0
MOU-MNP-16	1	13	16	2	0	13	5	1	4	3	10	16
MOU-MNP-17	7	19	37	38	8	18	13	7	9	9	69	44
MOU-MNP-18	10	7	25	2	3	18	19	7	10	28	21	47
MOU-MNP-19	40	30	19	108	23	23	27	3	53	77	74	99
MOU-MNP-20	21	3	4	42	3	87	18	7	8	59	13	1
MOU-G&D-9796	4	7	17	13	11	10	40	7	20	14	3	74
MOU-G&D-11571	12	23	37	1	9	18	21	5	16	16	33	53
MOU-G&D-7527-a	12	27	37	7	19	11	29	0	10	6	15	0
MOU-G&D-7527-b	7	9	8	17	4	1	25	5	9	11	18	90
MOU-G&D-6857	3	8	16	5	4	2	19	3	9	4	25	0
MOU-MNP-L&R-H7	26	13	17	90	8	83	6	3	7	34	28	70
MOU-MNP-L&R-H8	29	28	21	132	7	45	15	4	16	34	26	54
MOU-G&D-4584a1	23	19	10	75	3	16	28	9	14	57	54	100
MOU-G&D-5255a1	17	9	2	0	4	1	2	3	1	7	5	33
MOU-G&D-5902a2	8	0	5	13	5	2	15	3	8	6	5	40
MOU-G&D-6846a	14	16	13	4	7	25	22	8	17	25	24	10
MOU-G&D-8975	14	7	29	7	9	1	67	2	14	1	5	2
MOU-G&D-9858a	11	15	11	77	4	11	5	4	4	8	31	26
MOU-G&D-9858b	8	10	3	34	5	42	16	13	17	22	6	9
MOU-G&D-11869	8	22	33	37	4	26	8	3	7	9	109	115
MOU-G&D-11236b	51	20	50	0	7	88	12	11	9	12	6	9
MOU-G&D-12575a	14	32	32	4	7	25	12	4	17	12	6	9
MOU-G&D-13440	6	9	9	17	5	68	17	1	16	21	3	4

Values expressed as percentage.

Table G. Results of SEM-EDS analyses of Mn-rich lumps from Le Moustier

Piece	Description					Semi-quantitative EDS analyses (**)				Feature interpretation	General interpretation
	N	Feature morphology	BSE * contrast	Grain length (µm)	Grain width (µm)	>10%	3-10%	3-1%	<1%		
MOU-MNP-04	6	Blocky / Foliat.	Light grey	26.43	14.29	**Mn**			Ca, Si, Al, Fe, Ba, Sr, Zn, P, K, As, Mg, Na, Cl	Mn compound - Ba, Zn, As traces	Mn compound (Ba, Zn, As traces)
MOU-MNP-06	4	Acicular	White	Submicr.	Submicr.	**Mn**	Ba		K, Ca, Fe, Al, Sr, Si, Zn, P, Mg, S, As, Na, Cl	Mn/Ba compound - Zn, As traces	Mn/Ba compound (Zn, As traces with silicates and aluminosilicates)
	1	Irreg. tablet	Dark grey	22.48	17.06	Si, Fe	Al, Mn, Mg, K	Ba	Ti, Ca, Zn	Mg/K-rich aluminosilicate	
	1	Irreg. fac. cryst.	Dark grey	10.54	10.71	Si, Al	Mn	Ca, Fe, Ba	Mg, K, Zn, Ti	Na-rich silicate (feldspar)	
	1	Irreg. fac. cryst.	Dark grey	15.01	9.36	**Si**	Mn		Al, Ba, Fe, K, Zn, Mg	Silicate (quartz)	
MOU-G&D-6857	1	Irreg. tablet	Dark grey	92.86	57.87	Si, Al, Mn	K		Fe, Ba, Na, Mg, Ti, Ca, As	K-rich aluminosilicate (mica) - Ti, As traces	Mn + Mn/Ba compound (As traces) + aluminosilicates (Ti traces)
	1	Acic.	White	3	0.1	Mn, Si, Al	Ba, K	Fe	Mg, Na, Ti, Ca, P, S	Mn/Ba compound - Ti traces	
	2	Blocky	White	1.5	1.5	**Mn**		Si, Al, Fe	Ba, K, Ca, P, Mg, Ti, Na, S	Mn compound - Ba, Ti traces	

N: Number of analysed features; BSE: backscattered electrons; Irreg. fac. cryst: irregular facetted crystals; (*): White, grey and dark grey refer to the contrast observed on BSE images. (**) Weight percentatges including O and normalised to 100%. Elements in bold are present in a proportion equal or higher than 40 %.

Table H. Processing techniques, degree of modification, facet morphology, and striations main orientation of Mn-rich black lumps from Le Moustier

Piece	Percussion	Notching or scraping	Grinding	Smoothing	Degree of modification	Number of facets	Striations on facets	Facet morphology				Striation orientation				
								Flat	Convexe	Concave	Undet	Parallel	Oblique	Perpendicular	Absent	2 directions
MOU-MNP-01	No	No	Yes	No	Intense	1	Yes	1				1				
MOU-MNP-02	No	No	Yes	No	Intense	2	Yes		1		1	2				
MOU-MNP-03	No	No	Yes	No	Intense	3	Yes	1	1	1		1	1		1	
MOU-MNP-04	No	No	Yes	Yes	Intense	3	Yes	2	1				3			
MOU-MNP-05	No	No	Yes	No	Medium	3	Yes	2	1						2	1
MOU-MNP-06	Possible	No	No	Yes	NA	NA	No									
MOU-MNP-07	Yes	No	No	No	Low	NA	No									
MOU-MNP-08	No	No	Yes	No	Medium	1	Yes		1			1				
MOU-MNP-10	Yes	No	No	No	Low	NA	No									
MOU-MNP-12	No	Yes	Yes	Possible	Intense	4	Yes	2	1		1	1				3
MOU-MNP-13	No	No	Yes	No	Intense	4	Yes	1	2		1	3			1	
MOU-MNP-15	Yes	No	Yes	No	Medium	2?	Yes	1			1		1		1	
MOU-MNP-16	Yes	No	Yes	No	Intense	2?	Yes	1			1		1		1	
MOU-MNP-17	No	No	Possible	No	NA	1	No				1					
MOU-MNP-19	No	No	Yes	No	Medium	1	Yes	1					1			
MOU-MNP-20	No	No	Possible	Yes	NA	NA	Not visibles				1					
MOU-MNP-L&R-H7	No	Yes	No	No	Intense	3	No		1	2						
MOU-MNP-L&R-H8	No	No	Yes	No	Intense	4	Yes		3	1					4	
MOU-G&D-6846-a	No	No	Yes	No	Intense	2	Yes		2				1		1	
MOU-G&D-6846-b	No	No	Yes	Yes	Intense	2	Not visibles		2						2	
MOU-G&D-6846-c	No	No	Yes	Yes	Medium	1	Not visibles		1						1	
MOU-G&D-6857	No	No	Yes	Yes	Very intense	5	No		1	4			1		4	
MOU-G&D-7527-a	No	No	Yes	No	Low	1	Yes		1			1				
MOU-G&D-7527-b	No	No	Yes	No	Medium	1	Yes		1							1
MOU-G&D-4584-a3	Possible	No	No	No	NA	NA	No									
MOU-G&D-4584-a4	Possible	No	No	No	NA	NA	No									
MOU-G&D-4584-a5	Possible	No	No	No	NA	NA	No									
MOU-G&D-4584-a6	Possible	No	No	No	NA	NA	No									
MOU-G&D-9796	Yes	No	Yes	Yes	Medium	3	Not visibles		2	1					3	
MOU-G&D-11571	No	No	Yes	Yes	Medium	4	Yes		2	2		2			2	
MOU-G&D-8975	No	No	Yes	Yes	Intense	3	Yes		2	1		1			2	
MOU-G&D-9858-a	No	No	Yes	Yes	Medium	3	Yes		1	2			1		2	
MOU-G&D-11869	No	No	Yes	No	Intense	6	Yes		6				1			
MOU-G&D-11236-b	Possible	No	Yes	Yes	Low	3	Yes		1	1	1		2		1	
MOU-G&D-12575-a	No	No	Yes	Yes	Medium	2	Not visibles		1	1						

NA: not applicable

Table I. Results of EDXRF analysis of geological samples

Code	Si	K	Ca	Ti	Mn	Fe	Ni	Zn	As	Sr	Ba	Pb
ALB-01	7.5	0.39	20.1	0.161	6.6	0.001	*0.00061*	0.0277	0.0008	0.0135	1.08	0.0708
ALB-01	8.7	0.39	20.1	0.163	6.6	0.001	*0.00064*	0.0227	0.0008	0.0112	1.10	0.0680
ALB-01	9.3	0.38	20.1	0.168	6.5	0.001	*0.00063*	0.0248	0.0016	0.0099	1.07	0.0648
ALB-02	10.1	0.51	22.2	0.140	4.4	0.37	*0.00062*	0.0234	0.0025	0.0054	0.4094	0.0572
ALB-02	9.8	0.49	22.1	0.140	4.4	0.31	*0.00064*	0.0198	0.0015	0.0070	0.4256	0.0573
ALB-02	11.7	0.47	21.8	0.138	4.4	0.32	*0.00063*	0.0196	0.0017	0.0059	0.4210	0.0562
ALB-03	*2.4*	*0.01*	35.6	0.079	3.8	0.16	0.0061	0.0183	0.0006	0.0123	0.0765	0.0012
ALB-03	*2.4*	*0.01*	35.8	0.077	3.8	0.16	0.0067	0.0182	0.0006	0.0123	0.0886	*0.0008*
ALB-03	*2.4*	0.01	37.1	0.080	3.8	0.17	0.0064	0.0176	0.0007	0.0092	0.0763	*0.0009*
BEY-01	5.9	0.10	10.1	0.112	36.3	0.77	0.0545	0.1320	0.0281	0.0182	2.83	*0.0013*
BEY-01	4.4	0.12	10.4	0.109	36.8	0.76	0.0556	0.1326	0.0305	0.0190	2.85	*0.0013*
BEY-01	5.7	0.11	10.1	0.208	36.3	0.78	0.0542	0.1343	0.0305	0.0152	2.25	*0.0013*
BEY-02	18.0	0.41	12.3	0.088	7.4	0.37	0.0105	0.1850	0.0030	0.0038	0.0703	*0.0009*
BEY-02	18.0	0.42	12.3	0.088	7.4	0.38	0.0112	0.1838	0.0027	0.0047	0.0527	*0.0001*
BEY-02	19.1	0.40	12.3	0.088	7.3	0.43	0.0112	0.1833	0.0024	0.0044	0.0581	*0.0009*
BEY-02	18.0	0.41	12.1	0.087	7.4	0.38	0.0113	0.1778	0.0028	0.0058	0.0668	*0.0009*
BEY-03	4.8	0.07	9.4	0.228	32.4	0.60	0.0384	0.1064	0.0292	0.0104	2.05	*0.0010*
BEY-03	6.4	0.10	9.5	0.220	31.6	0.61	0.0377	0.1062	0.0280	0.0101	2.12	*0.0010*
BEY-03	5.8	0.06	9.3	0.225	31.5	0.60	0.0389	0.1029	0.0270	0.0087	2.04	*0.0010*
BEY-04	16.0	0.20	16.3	0.086	4.3	0.28	0.0035	0.1441	0.0010	0.0074	0.0196	*0.0009*
BEY-04	13.8	0.17	16.4	0.085	4.4	0.28	0.0036	0.1431	0.0011	0.0066	0.0194	*0.0009*
BEY-04	11.9	0.18	16.3	0.085	4.4	0.30	0.0047	0.1423	0.0017	0.0055	0.0292	*0.0009*
BEY-05	22.8	0.38	11.2	0.089	5.9	0.36	0.0109	0.2049	0.0019	0.0109	0.0126	0.0012
BEY-05	25.4	0.41	11.2	0.086	5.7	0.41	0.0095	0.2053	0.0018	0.0084	0.0369	*0.0009*
BEY-05	23.9	0.43	11.3	0.087	5.8	0.42	0.0103	0.2063	0.0024	0.0101	0.0292	*0.0009*
BEY-06	9.9	0.39	15.9	0.086	8.6	0.39	0.0173	0.2552	0.0035	0.0028	0.0856	*0.0009*
BEY-06	9.8	0.41	15.9	0.089	8.6	0.38	0.0198	0.2502	0.0030	0.0053	0.0613	*0.0001*
BEY-06	11.3	0.40	15.8	0.084	8.4	0.38	0.0177	0.2506	0.0035	0.0037	0.0790	*0.0009*
BOU-01	22.6	0.67	1.1	0.252	18.3	4.2	0.0750	0.0447	0.0076	0.0120	0.8103	0.0080
BOU-01	23.0	0.65	1.0	0.253	18.0	4.0	0.0726	0.0421	0.0082	0.0099	0.7774	0.0052
BOU-01	22.6	0.62	1.0	0.258	18.2	4.0	0.0728	0.0434	0.0071	0.0120	0.7168	0.0081
BOU-04	26.4	1.29	1.1	0.367	8.4	4.8	0.0149	0.0295	0.0079	0.0103	0.1103	0.0020
BOU-04	25.6	1.32	1.1	0.370	8.6	4.9	0.0177	0.0362	0.0070	0.0136	0.1230	0.0060
BOU-04	24.5	1.24	1.1	0.349	8.5	4.5	0.0167	0.0346	0.0068	0.0140	0.0991	0.0054
BOU-06	8.3	0.31	0.5	0.163	27.6	11.8	0.0125	0.0251	0.0166	0.0217	0.3608	*0.0016*
BOU-06	8.4	0.33	0.5	0.163	27.5	11.8	0.0118	0.0231	0.0169	0.0206	0.3383	*0.0016*
BOU-06	8.4	0.36	0.6	0.156	27.8	12.5	0.0135	0.0251	0.0172	0.0217	0.4252	*0.0017*
BOU-07	22.1	0.44	0.5	0.378	15.1	5.70	0.0213	0.0233	0.0096	0.0152	0.3121	*0.0012*
BOU-07	23.0	0.45	0.5	0.420	14.4	5.76	0.0218	0.0221	0.0094	0.0150	0.2704	*0.0012*
BOU-07	23.7	0.44	0.5	0.427	14.1	5.75	0.0208	0.0241	0.0098	0.0136	0.3022	*0.0012*
CAU-01	4.7	0.01	26.2	0.164	8.3	0.18	0.0102	0.0208	0.0036	0.0256	1.15	*0.0008*
CAU-01	4.2	0.02	26.8	0.169	8.4	0.15	0.0107	0.0218	0.0035	0.0275	1.19	*0.00078*
CAU-01	3.1	*0.01*	26.1	0.162	8.4	0.14	0.0102	0.0176	0.0036	0.0252	1.14	*0.00081*
CAU-02	*2.6*	0.13	31.7	0.059	7.7	0.84	0.0163	0.0197	0.0063	0.0702	1.42	*0.0010*
CAU-02	*2.6*	0.16	31.9	0.059	7.6	0.86	0.0171	0.0193	0.0066	0.0703	1.36	*0.0010*
CAU-02	*2.6*	0.17	32.0	0.041	7.6	0.86	0.0174	0.0214	0.0065	0.0679	1.44	*0.0010*
CAU-03	3.0	*0.01*	27.9	0.138	7.3	0.48	0.0164	0.0243	0.0109	0.0567	0.9533	*0.0008*
CAU-03	3.2	*0.01*	27.9	0.142	7.2	0.53	0.0151	0.0195	0.0111	0.0537	0.9277	*0.0008*
CAU-03	2.9	*0.01*	27.5	0.149	7.4	0.51	0.0164	0.0205	0.0108	0.0556	0.9296	*0.0008*
CAV-01	31.0	0.91	8.3	0.115	6.1	1.13	0.0008	0.1009	0.0020	0.0103	*0.0120*	0.0036
CAV-01	30.9	0.96	8.3	0.118	6.2	1.20	0.0023	0.0999	0.0023	0.0083	0.0120	0.0021
CAV-01	31.0	0.98	8.2	0.113	6.2	1.18	0.0022	0.1018	0.0018	0.0121	0.0374	0.0045
CAV-02	32.7	0.70	5.9	0.099	7.2	1.00	*0.0005*	0.0571	0.0022	0.0106	0.1010	0.0021
CAV-02	33.4	0.68	5.7	0.101	7.0	0.98	*0.0005*	0.0570	0.0021	0.0078	0.0982	0.0025
CAV-02	33.1	0.65	5.7	0.097	7.1	0.98	*0.0005*	0.0566	0.0022	0.0106	0.1136	0.0021
LOU-01	20.1	0.83	7.1	0.126	20.1	1.13	0.0158	0.5062	0.0033	0.0119	0.0477	*0.0001*

Code	Si	K	Ca	Ti	Mn	Fe	Ni	Zn	As	Sr	Ba	Pb
LOU-01	19.3	0.89	7.1	0.124	20.2	1.06	0.0165	0.5132	0.0033	0.0160	0.0499	*0.0011*
LOU-01	19.0	0.86	7.1	0.125	20.3	1.07	0.0173	0.5056	0.0017	0.0179	0.0527	0.0037
LOU-02	35.8	0.70	4.0	0.131	6.7	0.75	0.0217	0.2993	0.0021	0.0332	0.0706	*0.0010*
LOU-02	35.5	0.73	4.0	0.128	6.9	0.73	0.0223	0.3017	0.0020	0.0305	0.0906	*0.0010*
LOU-02	35.7	0.76	4.0	0.131	6.8	0.71	0.0211	0.3048	0.0020	0.0311	0.0893	*0.0010*
LOU-03	18.6	0.72	5.3	0.099	16.6	0.63	0.0073	0.3646	0.0022	0.0200	0.0632	*0.0001*
LOU-03	23.8	0.76	5.2	0.095	14.9	0.61	0.0051	0.3569	0.0020	0.0217	0.0618	*0.0001*
LOU-04	30.1	0.62	6.8	0.109	7.4	0.62	0.0031	0.2622	0.0015	0.0122	0.0565	*0.0010*
LOU-04	31.1	0.68	7.0	0.107	7.2	0.64	0.0026	0.2722	0.0014	0.0099	0.0613	*0.0010*
LOU-04	30.7	0.61	6.5	0.105	7.2	0.61	0.0021	0.2552	0.0012	0.0107	0.0658	*0.0010*
SAL-01	*2.50*	0.01	30.8	0.146	11.1	0.06	0.0275	0.0302	0.0043	0.0763	1.21	*0.0010*
SAL-01	*2.50*	*0.01*	30.8	0.148	11.1	0.07	0.0260	0.0257	0.0045	0.0759	1.21	*0.0010*
SAL-01	*2.50*	*0.01*	30.7	0.149	11.2	0.05	0.0264	0.0319	0.0045	0.0784	1.21	*0.0010*
SAL-02	*2.3*	0.10	33.8	0.099	5.6	0.19	0.0048	0.0150	0.0008	0.2138	0.4577	*0.0008*
SAL-02	2.6	0.11	33.5	0.101	5.4	0.19	0.0027	0.0150	0.0008	0.2083	0.4231	*0.0001*
SAL-02	*2.3*	0.07	32.7	0.095	5.5	0.18	0.0040	0.0150	0.0009	0.2043	0.4464	*0.0008*
SAL-03	2.7	0.13	32.6	0.093	5.5	0.23	0.0027	0.0150	0.0007	0.2090	0.4451	*0.0008*
SAL-03	2.4	0.12	32.7	0.093	5.5	0.22	0.0029	0.0150	0.0007	0.2072	0.4427	*0.0008*
SAL-03	*2.3*	0.09	32.5	0.092	5.5	0.21	0.0042	0.0150	0.0009	0.2012	0.4613	*0.0008*
SAR-01	30.7	0.79	0.1	0.225	10.5	0.65	*0.0006*	0.0150	0.0031	0.0185	0.8630	*0.0001*
SAR-01	30.3	0.76	0.1	0.226	10.2	0.63	*0.0006*	0.0164	0.0037	0.0176	0.8793	*0.0012*
SAR-01	28.8	0.76	0.1	0.248	9.5	0.65	*0.0006*	0.0231	0.0024	0.0206	0.6916	*0.0012*
TEY-01	7.4	0.47	2.1	0.240	35.5	3.7	0.0309	0.0377	0.0100	0.0061	1.70	*0.0012*
TEY-01	4.6	0.45	2.1	0.241	37.1	3.6	0.0334	0.0369	0.0111	0.0055	1.70	*0.0012*
TEY-01	4.9	0.45	2.1	0.239	36.9	3.6	0.0343	0.0385	0.0090	0.0080	1.74	*0.0012*
THE-02	*2.6*	*0.01*	38.5	0.166	6.8	0.24	0.0091	0.0328	0.0012	0.0027	1.31	*0.0010*
THE-02	*2.6*	*0.01*	38.8	0.172	6.7	0.21	0.0104	0.0356	0.0006	0.0048	1.25	0.0010
THE-02	*2.6*	*0.01*	38.9	0.173	6.5	0.23	0.0074	0.0354	0.0011	0.0021	1.26	*0.0001*
THE-03	*2.6*	0.02	27.2	0.134	13.9	0.25	0.0171	0.0340	0.0020	0.0101	2.54	*0.0010*
THE-03	*2.6*	0.02	27.3	0.127	13.7	0.23	0.0169	0.0348	0.0027	0.0100	2.54	*0.0010*
THE-03	*2.6*	*0.01*	27.4	0.148	13.8	0.28	0.0157	0.0300	0.0026	0.0084	2.48	*0.0010*
THE-06	8.1	0.27	19.5	0.455	12.2	3.1	0.0204	0.0568	0.0152	0.0123	2.55	0.0023
THE-06	7.9	0.29	19.6	0.454	12.1	3.1	0.0223	0.0566	0.0154	0.0103	2.53	0.0015
THE-06	7.6	0.26	19.5	0.459	12.3	3.1	0.0213	0.0584	0.0153	0.0110	2.52	0.0027
TRA-01	12.4	0.16	0.3	0.246	24.6	*0.0036*	0.5741	0.3041	0.0146	*0.0006*	0.4858	0.1919
TRA-01	14.1	0.16	0.3	0.245	24.0	*0.0036*	0.5692	0.3058	0.0107	*0.0006*	0.4699	0.1986
TRA-01	11.6	0.16	0.2	0.238	24.5	*0.0035*	0.5740	0.2980	0.0119	*0.0006*	0.5181	0.1949
VER-01	*2.5*	0.16	6.7	0.004	51.6	0.21	0.0123	0.0220	0.0005	0.0022	1.15	0.0025
VER-01	*2.5*	0.18	6.6	0.004	50.9	0.24	0.0138	0.0223	0.0006	0.0012	1.12	*0.0015*
VER-01	*2.5*	0.18	6.7	0.020	50.2	0.21	0.0123	0.0222	0.0006	0.0010	0.9430	*0.0016*

Values expressed as percentage. Figures in italics indicate concentrations below the limit of detection calculated multiplying the equipment detection limit by 0.6 [106]. Key: ALB: Albas; BEY: Beyssac; BOU: Bouzic; CAU: Causse; CAV: Cave; LOU: Loup; SAL: Sals; SAR: Sarlat; TEY: Teyjat; THE: Theil; TRA: Tranchecouyère; VER: Verdier.

Table J. Results of SEM-EDS analyses of the geological samples

Piece	Analysed feature	N	Grain size (µm)	Semi-quantitative EDX analyses (*)			Interpretation
				>10%	1-10%	<1%	
ALB-02	White acicular crystals randomly distributed	3	Sub-microm.	Mn, Ba	(Fe, Ca, Si, Al)	(Mg, K)	Complex Ba-rich manganese compound + (calcium carbonate + alumino-silicates)
	White acicular crystals randomly distributed over stacked platelets	1	Sub-microm.	Mn, Ba	(Ca, Fe, Si, Al)	(K, Mg, Na)	Complex Ba-rich manganese compound + (calcium carbonate + alumino-silicates)
	Dark grey reg. fac. cryst.	2	12.7	Ca	(Mn, Si, Ba, Al)	(K, Mg)	Calcium carbonate + (complex Ba-rich manganese compound + alumino-silicates)
ALB-03	White agglomerate of stacked chips	3	Microm. to sub-microm.	Mn, (Ca)	Ba, (K, Al, Si)	(Fe), Cl, Na, (Mg)	Complex Ba-rich manganese compound + chlorides + (calcium carbonate + alumino-silicates)
	Dark grey blocky crystals	3	42.5	**Ca**	(Mn, Si, Al)	(K, Mg)	Calcium carbonate + (Mn-rich compound + alumino-silicates)
BEY-01	White agglomerates composed of irreg. fac. cryst.	3	-	**Mn**, Ba	(Ca)	(Si, Al, Na, S, Mg, K)	Complex Ba-rich manganese compound + (alumino-silicates + calcium carbonate + sulphates)
	Light grey lath-like crystals	3	12	**Mn**	Ba, (Ca)	(Si, Al, Na)	Complex Ba-rich manganese compound + (alumino-silicates + calcium carbonate)
	White agglom. composed of irreg. fac. cryst. and acicular cryst.	3	Submicr.	**Mn**	Ba, (Ca)	(Si, Al, Na, Mg, K, P)	Complex Ba-rich manganese compound + (alumino-silicates + calcium carbonate)
	Grey blocky crystals	3	≤50	**Ca**, (Mn)	(Ba)	(Si, Al, P)	Calcium carbonate + (complex Ba-rich manganese compound + alumino-silicates + phosphates)
BOU-03	Light grey agglomerate of subcircular irreg. fac. cryst.	3	2.6	Mn, Fe	(Si, Al, Ca, Mg)	(K)	Mn/Fe-rich compound + (alumino-silicates)
	Compact agglomerate of stacked platelets	1	4.5	**Fe**, Si	Al, Mn	K, Ca, Mg	Fe-rich alumino-silicate with traces of Mn
BOU-04	Stacked light grey platelets with tiny irreg. fac. white crystals	3	5.7	Si, Al	Fe, Mn, Ca, Mg	Ti	Fe/Mn-rich alumino-silicate + Ti-rich compound
	White irreg. fac. cryst.	1	5	Ce	Si, La, P, Th, Nd, Al, Fe, Ca	K, Mg	Ce/La/Th/Nd/Ca-rich silicate with phosphate + (alumino-silicates)
	Light grey irreg. fac. cryst. over stacked platelets	1	0.8	Fe, Si, Al	Ca, K, Mg	Mn, Ti	Ti-rich compound + Fe-rich alumino-silicate with traces of Mn
	Light grey irreg. fac. cryst. over stacked platelets	1	0.5	Si, Ti, Al	Fe, Ca, K, Mg	Mn	Ti-rich compound + Fe-rich alumino-silicate with traces of Mn
	Grey irreg. fac. cryst.	2	44	Ca, Mg	(Si, Al, Fe, Mn)	(K, Ti)	Ca/Mg-rich carbonate + (Fe/Mn-rich alumino-silicate + Mn/Ti-rich compound)
	Agglomerates of light grey poorly crystallized rounded particles	3	3.1	Si, Mn, Al	Fe, Ca, K, Mg	(Ti), Na	Mn/Fe-rich alumino-silicate + (Ti-rich compound)
BOU-06	Agglomerate of white acicular crystals	1	Sub-microm.	**Mn**	(Fe), Ba, (Si), Ca, (Al)	(Mg, K)	Complex Ca/Ba-rich manganese compound(s) + (Fe-rich alumino-silicate)
	Light grey gel-like mass	1	-	**Fe**	(Mn), Si, Al,	Mg, (P), (Ca), (Ba), K	Fe-rich alumino-silicate + (complex Ca/Ba-rich manganese compound + phosphates)
	Agglomerate of grey reg. fac. cryst.	1	1.5	Mn, (Fe)	(Si, Al)	Ba, Ca, (Mg, K, P)	Complex Ca/Ba-rich manganese compound(s) + (Fe-rich alumino-silicate + phosphates)

Piece	Analysed feature	N	Grain size (µm)	Semi-quantitative EDX analyses (*)			Interpretation
				>10%	1-10%	<1%	
	Stacked light grey chips	1	Microm.	**Mn**	(Fe, Si, Al)	Ca, (K), Ba, (Mg, P)	Complex Ca/Ba-rich manganese compound(s) + (Fe-rich alumino-silicate + phosphates)
	Stacked light grey platelets	1	2	Si, Al, Fe	(Mn), K, Mg	(Ca, Ba)	Fe-rich alumino-silicate + (complex Ca/Ba-rich manganese compound)
CAU-01	Grey blocky crystals	3	350	Ca (Mn)	(Ba)	(Si, Al, Fe, Mg, K, P, Na)	Calcium carbonate + (silicate/alumino-silicate + complex Ba-rich manganese compound + iron oxide + phosphates)
	White platelets and acicular crystals	3	5	Mn, (Ca)	Ba	(Si, Mg, Al, K, P, Na)	Complex Ba-rich manganese compound + (silicates/alumino-silicates + calcium carbonate + phosphates)
CAV-01	White amorphous mass	2	-	**Mn**, Ca	(Si), P, (Al)	(K, S, Na, Mg)	Complex manganese phosphate compound + (silicates/alumino-silicates + sulphates)
	White spherical agglomerate composed of irregular grains	1	-	**Fe**, Mn	Si, Ca, Al	P, K, Mg, Ti, Na, S	Complex ferroan-manganese compound + silicates/alumino-silicates, phosphates, sulphates
	Grey fibrous/laminated crystals	3	-	Si, K, Al	(Mn), Ca	Fe, (P), Na, Mg	K-rich silicate
	White gel-like mass at the surface / white spongy-like structure inside	3	-	Mn, Si	Al, Ca, Fe, K	Mg, Na, (P)	Complex manganese silicate compound
LOU-01	White botryoidal agglomerates	4	-	**Mn**	Ca, Al, Si, Zn	K, Na, P, Mg, S	Complex manganese compound
	White amorphous mass - cracked surface	3	-	Mn	Ca, Al, Si, Zn	K, Fe, P, Na, Mg	Complex manganese compound
SAL-01	Grey blocky crystals	4	350	**Ca**, (Mn)	(Ba)	(K, Mg, Al, Si, P, Na, S)	Calcium carbonate + (complex Ba/K-rich manganese compound + silicates/alumino-silicates + phosphates + sulphates)
	White acicular crystals displayed in a dendritic pattern	3	5	**Mn**	Ba, (Ca, Sr), K	(Al, Mg, Si)	Complex Ba/K-rich manganese compound + (silicates/alumino-silicates + calcium carbonate)
SAL-02	Grey blocky crystals	3	350	**Ca**	(Mn)	(Ba, K, Si)	Calcium carbonate + (complex Ba/K-rich manganese compound)
	White poorly crystallized crystals	3	-	**Mn**, (Ca)	Ba, K	(Al, Si, Mg)	Complex Ba/K-rich manganese compound + (alumino-silicates + calcium carbonate)
SAR-01	White acicular crystals	1	10	**Mn**	(Si), Ba	(Al, Ca, P)	Complex Ba-rich manganese compound + (silicates/alumino-silicates + phosphates)
	Grey subcirc. grains	1	≤1000	**Si**, (Mn)		(Al)	Silicate + (manganese compound)
	White agglom. composed of pseudo-regular grains	1	<2	**Mn**	(Si), Ba	(Ca, Al)	Ba-rich manganese compound + (silicates/alumino-silicates)
	White spongy structure	2	-	**Mn**	Ba, (Si)	(Al, Ca, K)	Ba-rich manganese compound + (silicates/alumino-silicates)
	White agglom. composed of poorly crystallized grains	1	-	**Mn**, Ba	(Si, Al)	(Ca, K)	Ba-rich manganese compound + (silicates/alumino-silicates)
	White agglom. composed of acicular crystals	1	7	**Mn**, Ba	(Si)	(Ca, Al, K)	Ba-rich manganese compound + (silicates/alumino-silicates)
THE-02	Dark grey crystal with a spongy structure	3	-	Mn, Ca	Ba, Si	Al, Fe, K, S, Mg, P	Complex Ba-rich manganese compound
	White agglomerates composed of platelets	3	10	Mn, Ca, Ba	Si	Al, K, Na, Mg, S, P, Cl	Complex Ba-rich manganese compound

Piece	Analysed feature	N	Grain size (µm)	Semi-quantitative EDX analyses (*)			Interpretation
				>10%	1-10%	<1%	
THE-03	Light grey amorphous crystals - porous surface	3	-	**Ca**, (Mn)	(Ba)	(Si, Al, P, Mg)	Calcium carbonate (complex Ba-rich manganese compound)
	Grey irreg. fac. cryst.	3	-	**Ca**, (Mn)	(Ba)	(Si, Al, Cl, Mg)	Calcium carbonate (complex Ba-rich manganese compound + chlorides)
	White agglomerates composed of platelets	3	10	**Mn**, Ba, (Ca)		(P, Si, Al)	Complex Ba-rich manganese compound
THE-06	White flower-like agglomerate composed of platelets	3	15	Ba, (Fe, Si)	(Al, Ca, Mn, S, K)	(Mg, Na)	Ba-rich compound
	Dark grey irreg. fac. cryst.	2	60	Ca, Fe	Mn, Si, Al, Ba	K, Mg, S	Undetermined
	Platelets randomly distributed	4	2	**Mn**, Ba	(Fe, Si, Al, Ca)	(S, K, Mg, Na)	Complex Ba-rich manganese compound
VER-01	White spheres with a spongy-like structure	3	1 - 60	**Mn**	Ca, Ba	K, (Si)	Complex Ca/Ba/K-rich manganese compounds + (silicates)

N: number of analysed features. (*) Weight percentatges including O, normalized to 100%. 'White' and 'Grey' refer to the contrast observed on backscattered electron (BSE) images. Elements in bold police refer to those which present a concentration over the 40%. Elements and compounds in braquets refer to those which do not account for the elemental/mineralogical composition of the analyzed item. Reg. or irreg. fac. cryst.: regular or irreular facetted crystal; Microm: micrometric.
