# Supplementary material for: Provenance, modification and use of manganese-rich rocks at Le Moustier (Dordogne, France)
Source: PLoS One. 2019 Jul 17;14(7):e0218568. doi: 10.1371/journal.pone.0218568 (PMC6636720; doi:10.1371/journal.pone.0218568)
Supplement: S1 Fig — (PDF) [file pone.0218568.s002.pdf]

Provenance, modification and use of manganese-rich rocks at Le Moustier (Dordogne, France)

Africa Pitarch Martí*, Francesco d'Errico, Alain Turq, Eric Lebraud, Emmanuel Discamps, Brad Gravina

*Corresponding Author:

E-mail: africa.pitarch@gmail.com (APM)

S1 Figures

Additional information on the EDXRF calibration, the archaeological Mn-rich lumps from Le Moustier and the Mn-rich geological samples collected at the Dordogne and Lot regions of France

Figure A. Plan and stratigraphy of Le Moustier…………………………………………………….2

Figure B. EDXRF calibration curves..………………………………………………………………...3

Figure C. Modifications on MOU-MNP-L&R-H7..…………………………………………….....4

Figure D. Modifications on MOU-MNP -L&R -08…………………………………………………..5

Figure E. Modifications on MOU-G&D-6857……………………………………………………….6

Figure F. Dimensions vs. number of facets………………………………………………………….7

Figure G. Mn/Si ration vs. length and weight…………………………………………………......8

Figure H. Mn/Si ration vs. type of modification and number of facets………………………...9

Figure I. Ternary plots of selected elements from the geological samples……………………....10

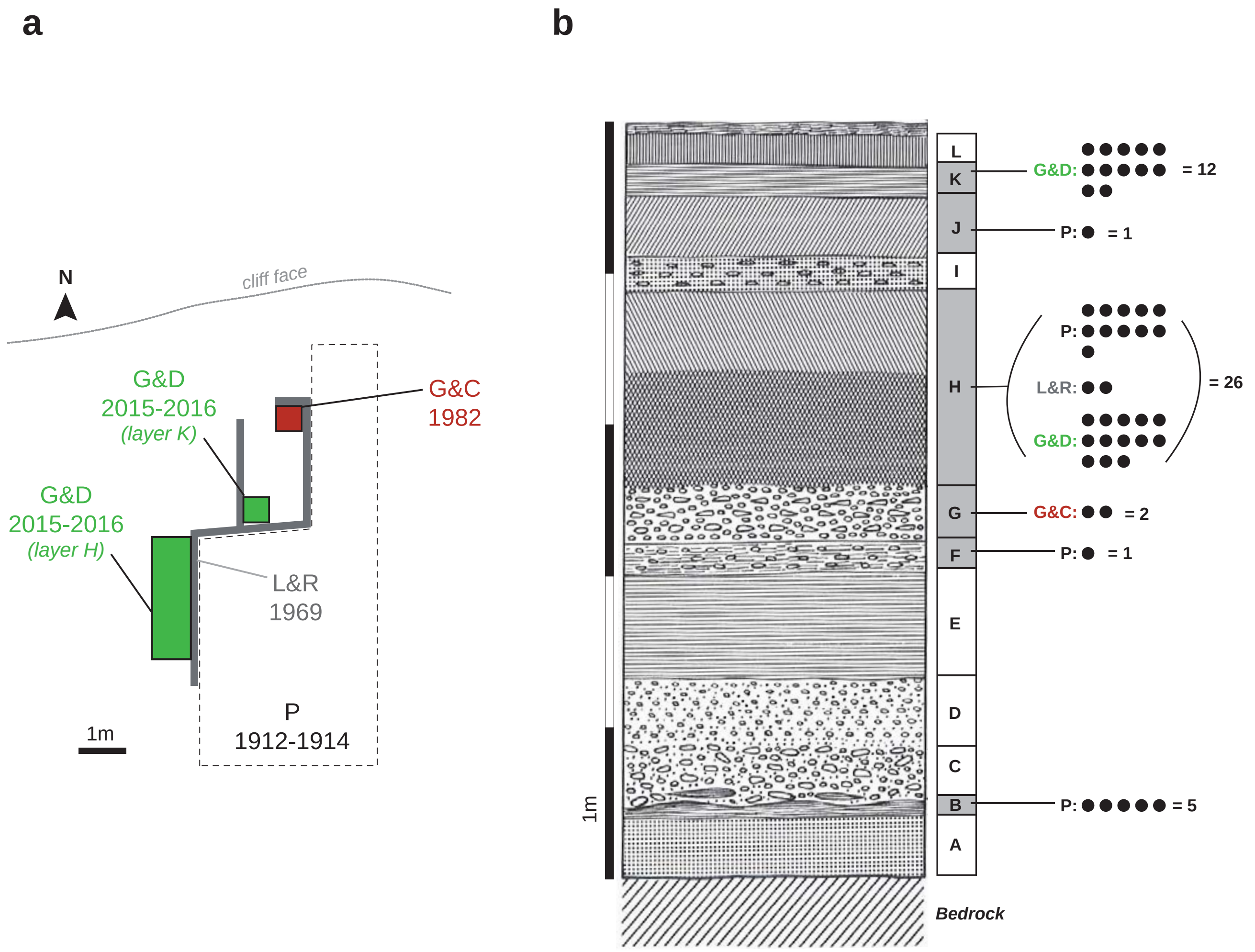

Figure A. Plan and stratigraphy of Le Moustier. a) Plan of the Le Moustier Lower Shelter indicating the area excavated by Peyrony (P, dashed lines), the section cleaned by Laville and Rigaud (L&R; light grey solid lines), the test-pit of Geneste and Chadelle (G&Ch, red square), and the recent excavations conducted by Gravina and Discamps (G&D; green squares); b) Peyrony's stratigraphy with the distribution of Mn-rich fragments (dots).

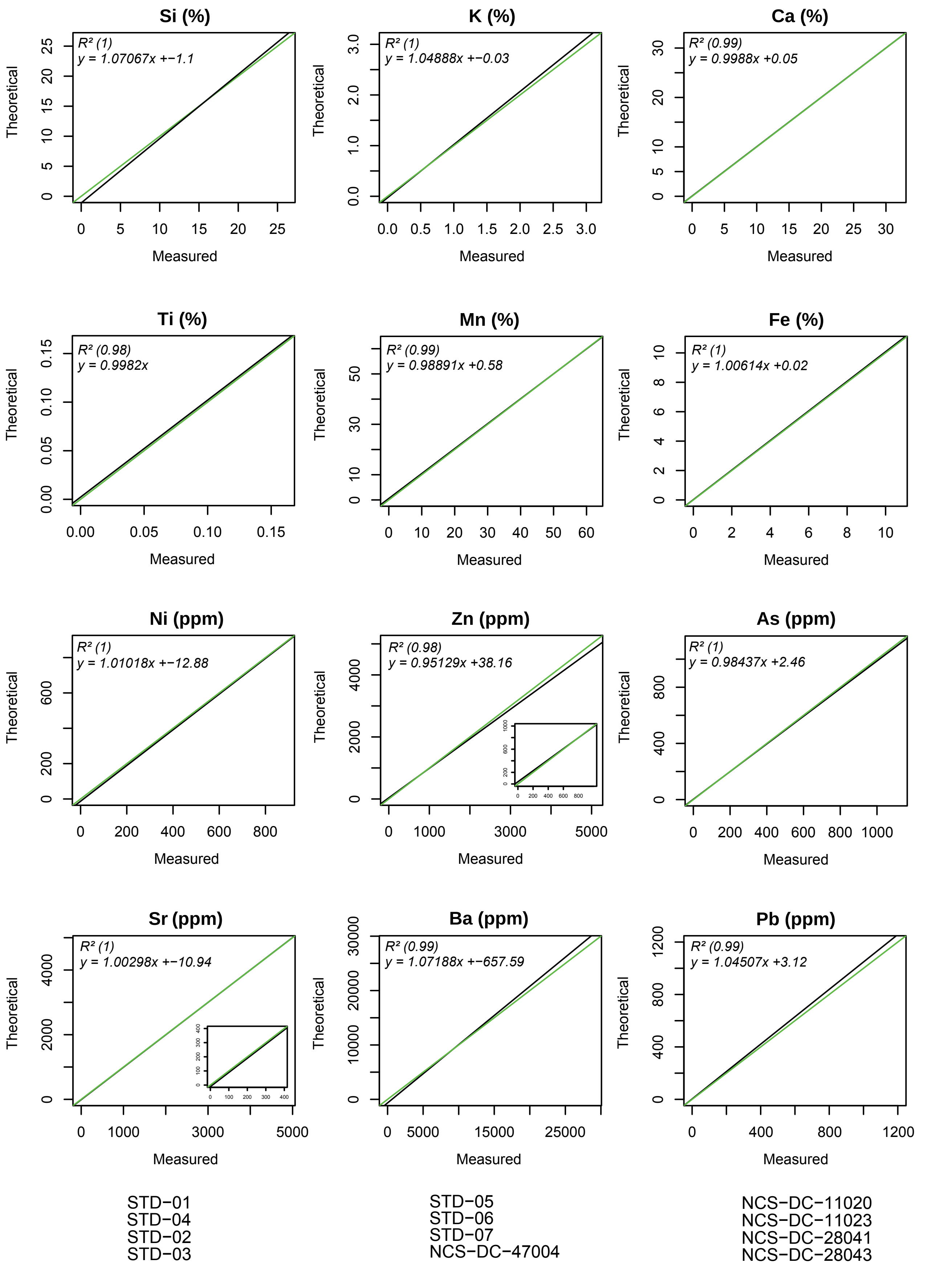

Figure B. EDXRF calibration curves. Calibration curves for the twelve elements analysed by EDXRF in this study.

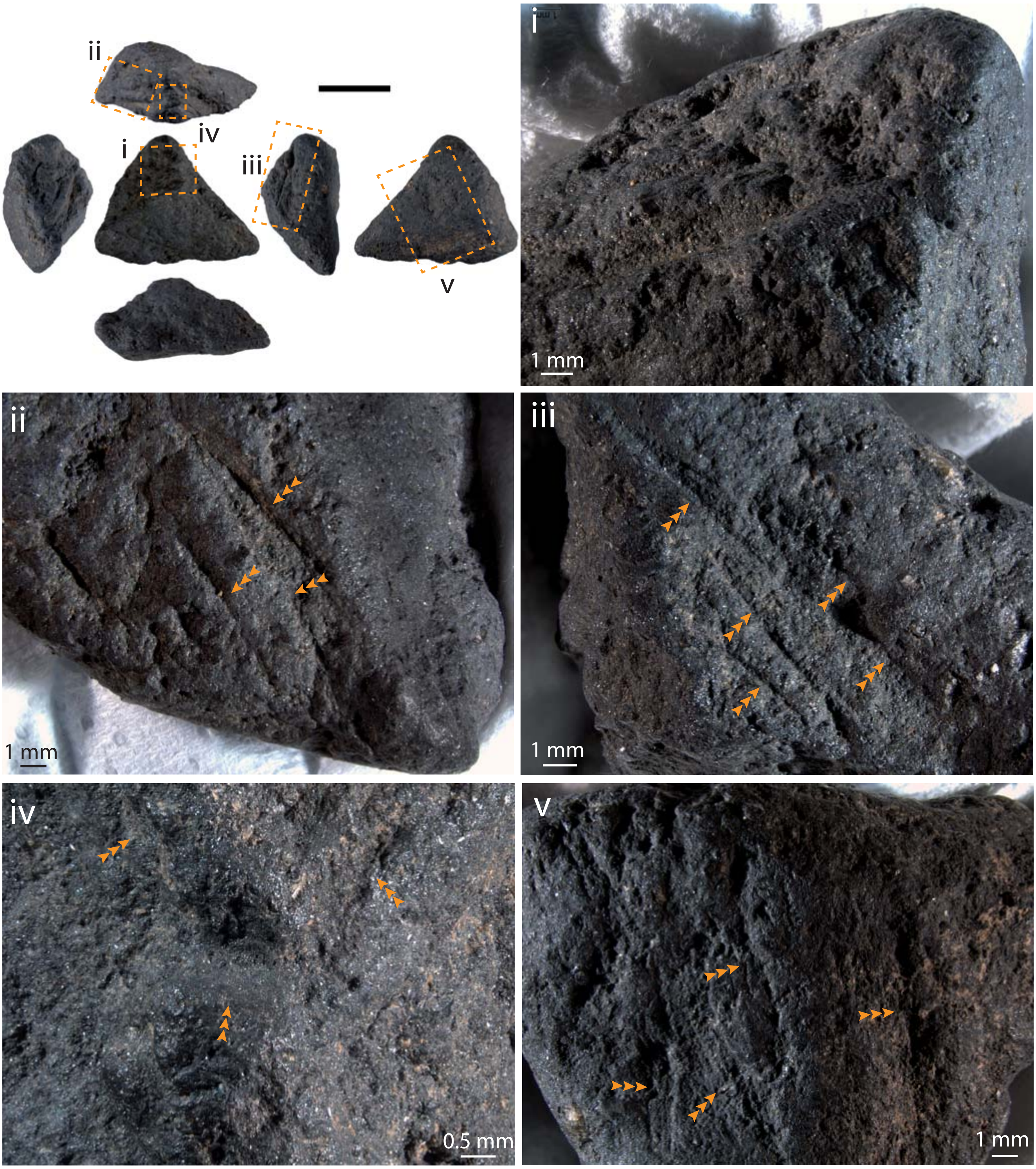

Figure C. Modifications on MOU-MNP-L&R-H7. Anthropogenic modifications recorded on the sample.

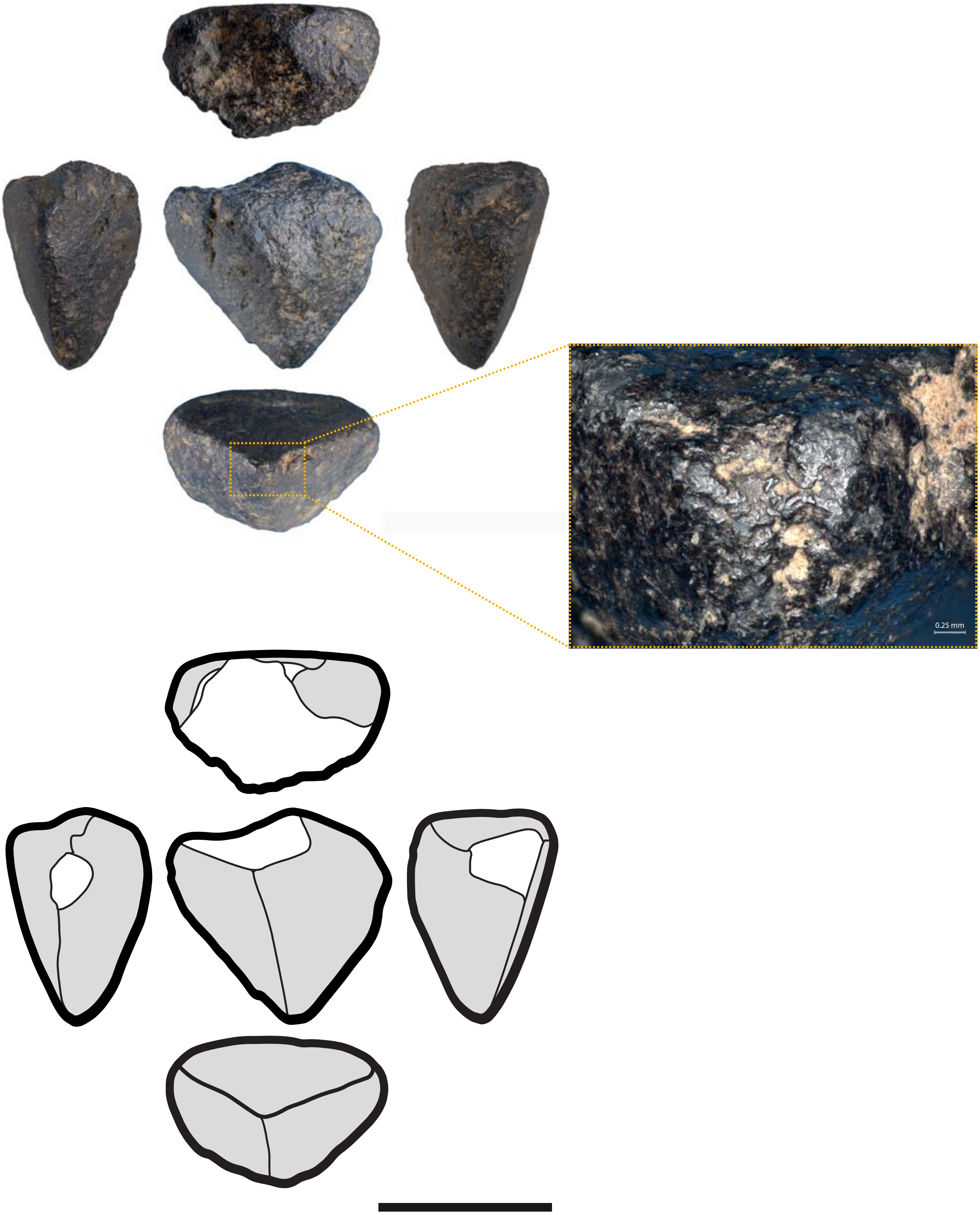

Figure D. Modifications on MOU-MNP -L&R -08. a) Photo of the object; b) tracing of the sample indicating ground facets (gray) and flake scars (white); c) photo of the tip bearing a polish.

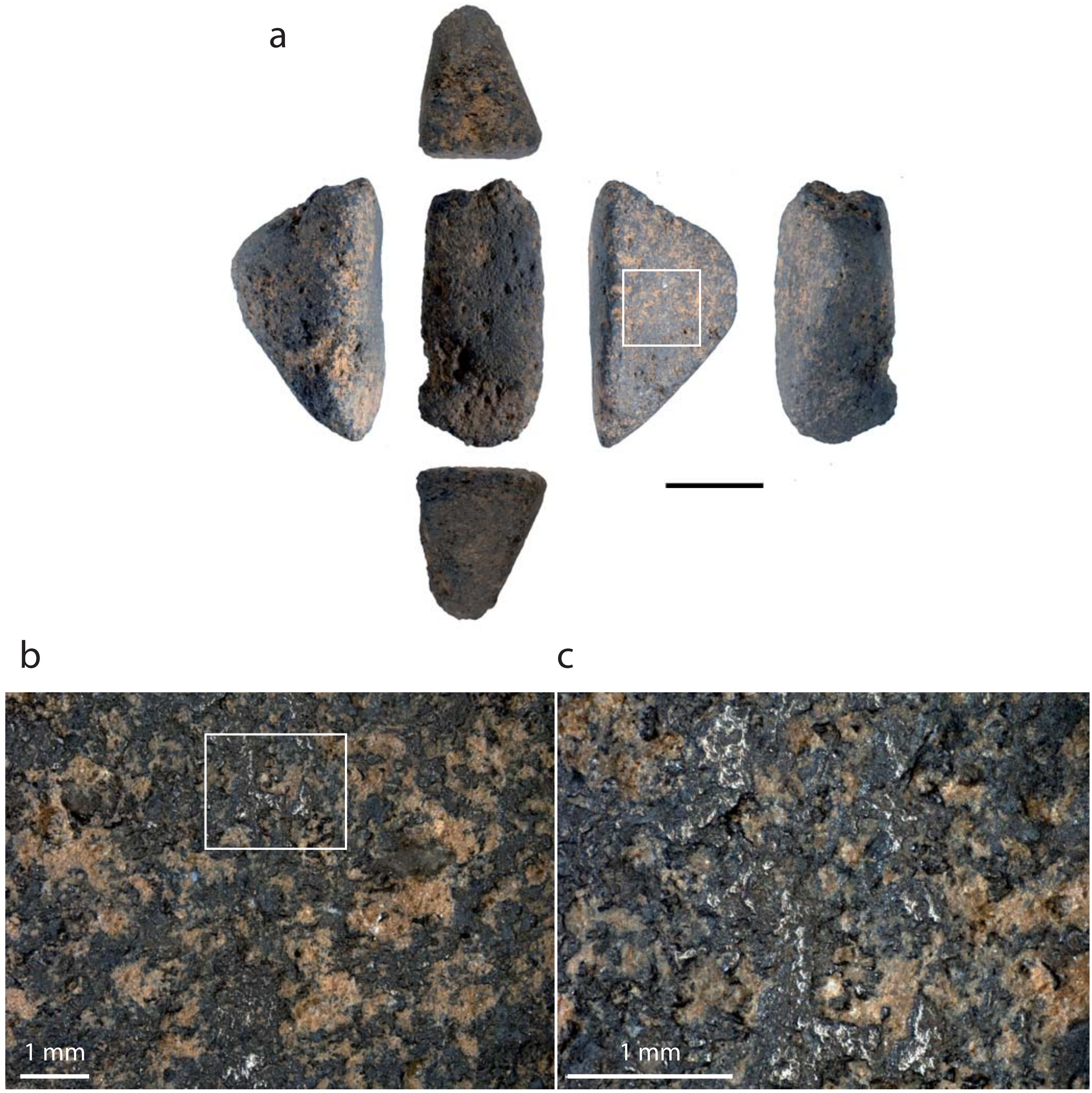

Figure E. Modifications on MOU-G&D-6857. Anthropogenic modifications recorded on the sample.

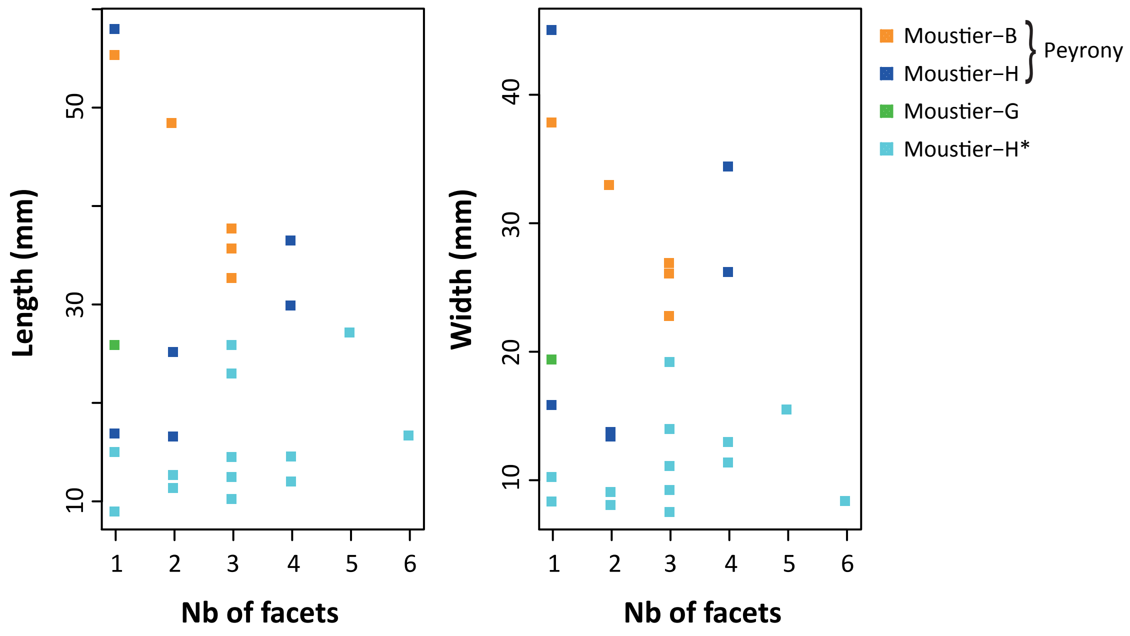

Figure F. Dimensions vs. number of facets. Size of modified manganese pieces by number of facets. Colours indicate the layer of provenance.

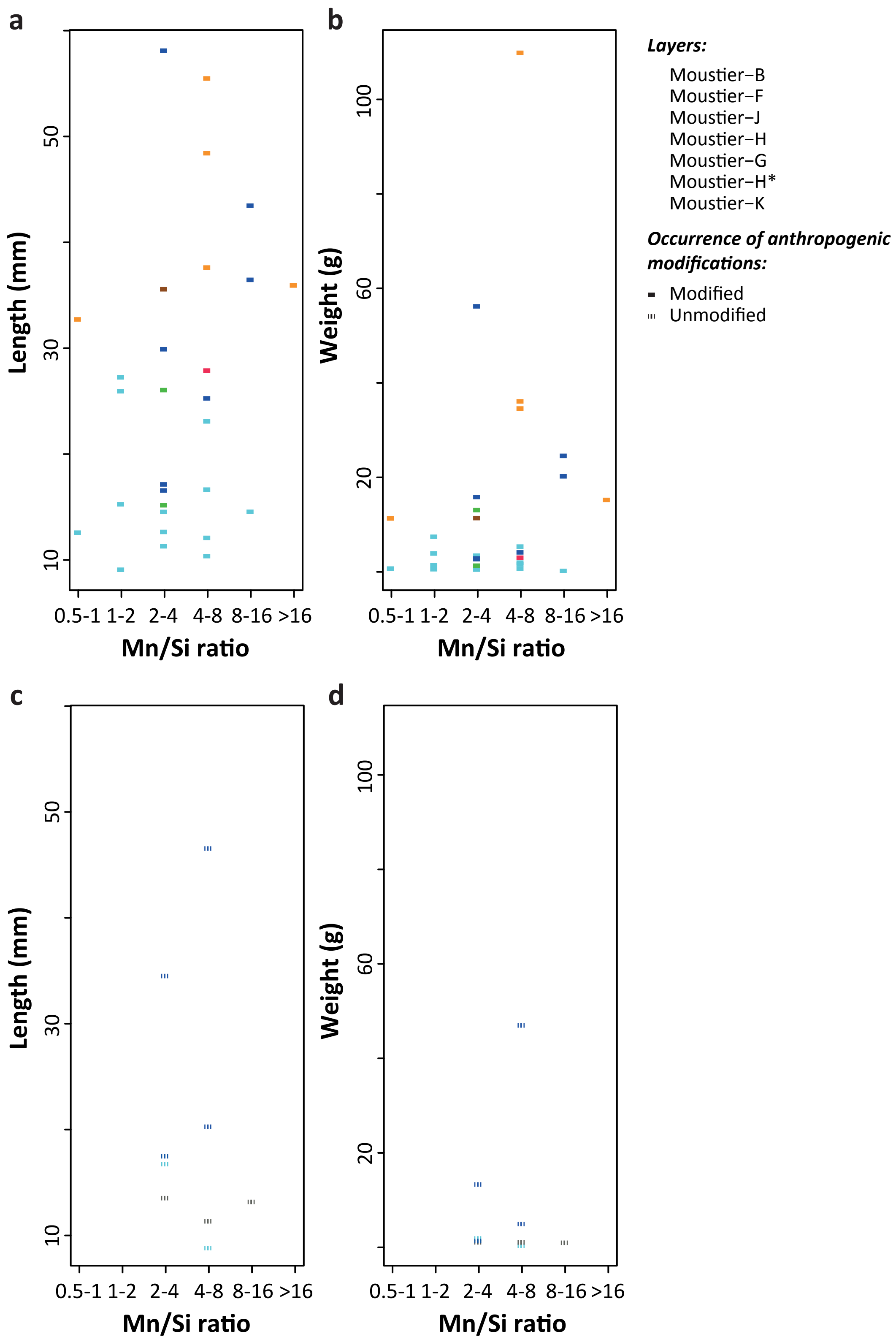

Figure G. Mn/Si ration vs. length and weight. Mn/Si ration of modified and unmodified manganese pieces from Le Moustier by length and weight. Colours indicate the layer of provenance.

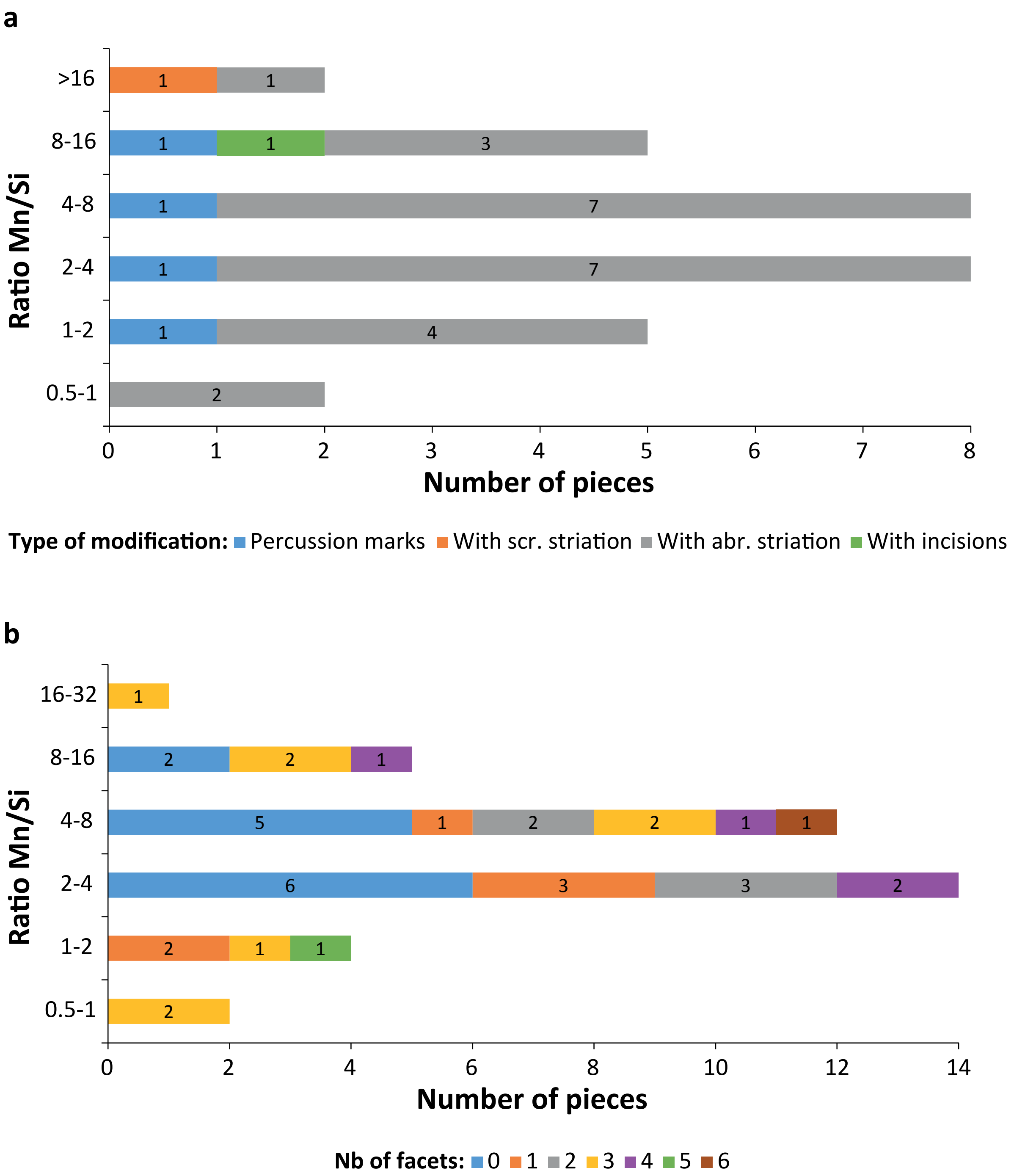

Figure H. Mn/Si ratio vs. type of modification and number of facets. Mn/Si ratio of modified archaeological lumps by modification type (a) and number of facets (b).

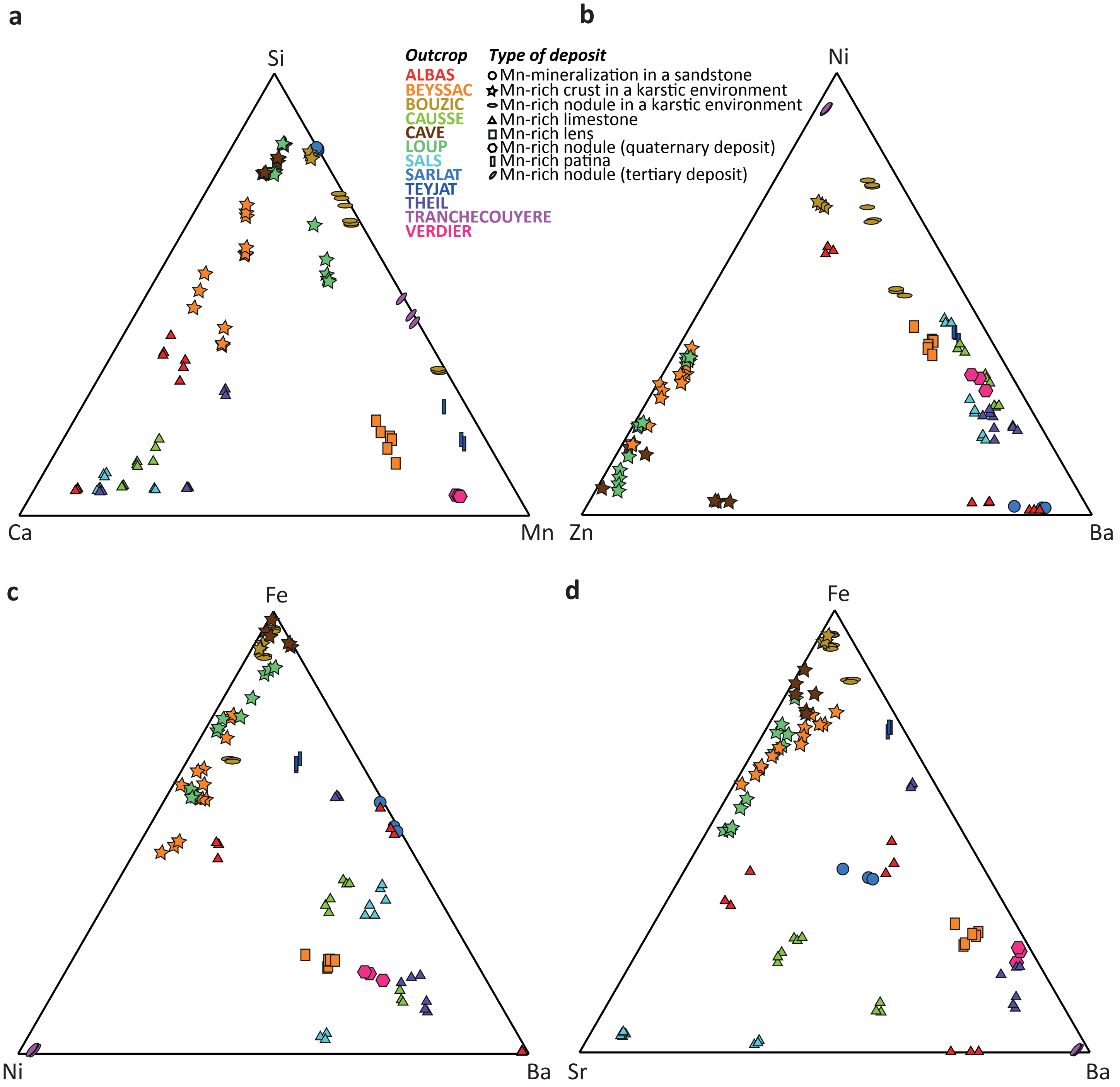

Figure I. Ternary plots of selected elements detected the geological samples. Ternary diagrams showing the composition of geological samples for (a) Ca-Si-Mn; (b) Zn, Ni, Ba; (c) Ni, Fe, Ba; (d) Sr, Fe, Ba. Data perturbation: (a) none, (b) 5-40-1, (c) 30-1-1, and 30-1-1 (d). Outcrops and type of deposits are indicated with different colours and symbols.
