# Supplementary material for: Provenance, modification and use of manganese-rich rocks at Le Moustier (Dordogne, France)
Source: PLoS One. 2019 Jul 17;14(7):e0218568. doi: 10.1371/journal.pone.0218568 (PMC6636720; doi:10.1371/journal.pone.0218568)
Supplement: S2 Fig — (PDF) [file pone.0218568.s003.pdf]

Provenance, modification and use of manganese-rich rocks at Le Moustier, Dordogne, France

Africa Pitarch Martí*, Francesco d'Errico, Alain Turq, Eric Lebraud, Emmanuel Discamps, Brad Gravina

*Corresponding Author

E-mail: africa.pitarch@gmail.com (APM)

S2 Figures

Results of analyses conducted on Mn-rich archaeological pieces from Le Moustier

Figure A. Results of analyses conducted on MOU-MNP-03....2
Figure B. Results of analyses conducted on MOU-MNP-04....3
Figure C. Results of analyses conducted on MOU-MNP-05....4
Figure D. Results of analyses conducted on MOU-MNP-06....5
Figure E. Results of analyses conducted on MOU-MNP-09....6
Figure F. Results of analyses conducted on MOU-MNP-11....7
Figure G. Results of analyses conducted on MOU-MNP-12....8
Figure H. Results of analyses conducted on MOU-MNP-19....9
Figure I. Results of analyses conducted on MOU-MNP-20....10
Figure J. Results of analyses conducted on MOU-MNP-L&R-H7....11
Figure K. Results of analyses conducted on MOU-MNP-L&R-H8....12
Figure L. Results of analyses conducted on MOU-G&D-5983....13
Figure M. Results of analyses conducted on MOU-G&D-6846....14
Figure N. Results of analyses conducted on MOU-G&D-6857....15
Figure O. Results of analyses conducted on MOU-G&D-7527....16
Figure P. Results of analyses conducted on MOU-G&D-9796....17
Figure Q. Results of analyses conducted on MOU-G&D-11571....18

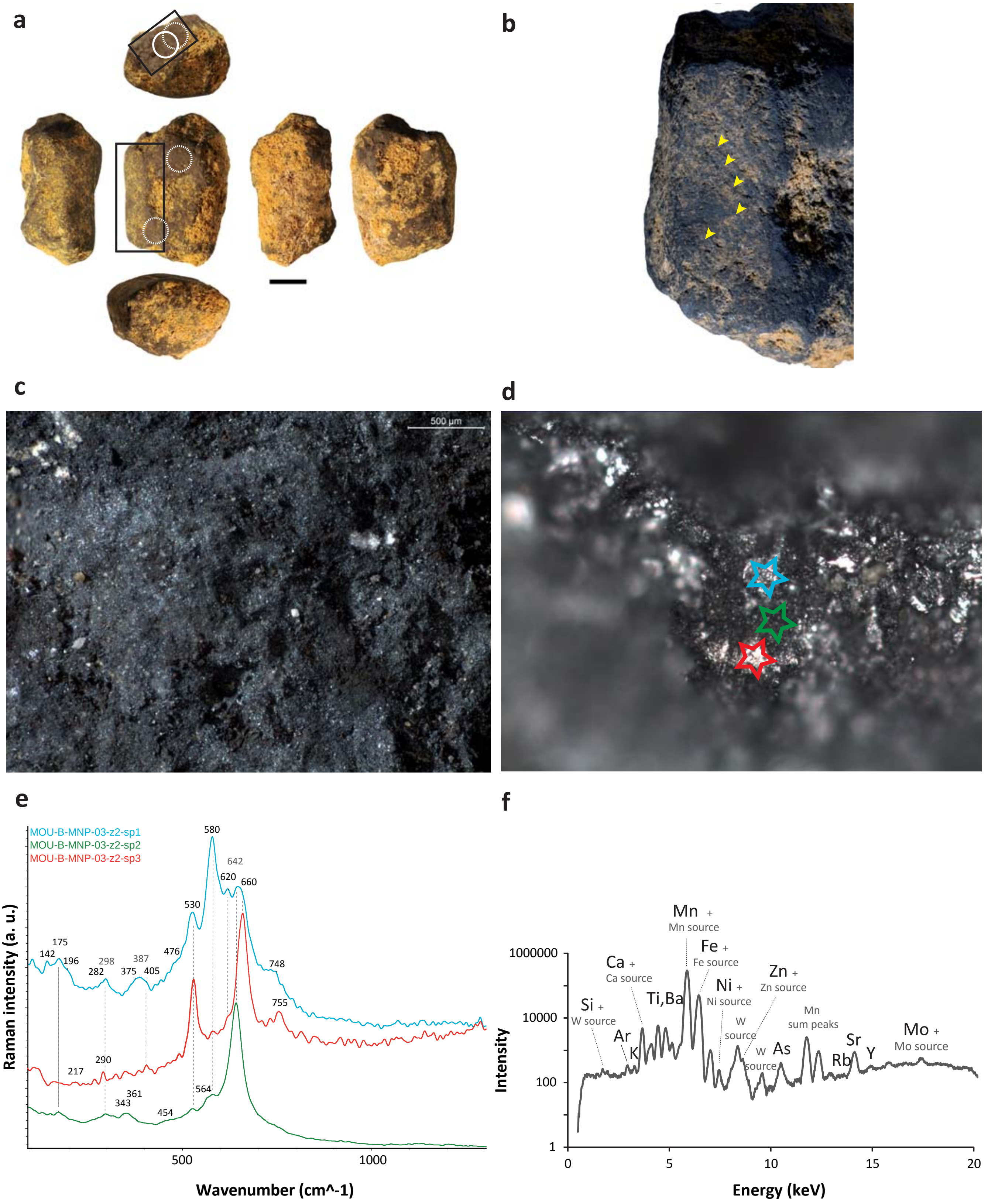

Figure A. Results of analyses conducted on MOU-MNP-03. a: photo of the lump. Squares indicate the position of macro photos (b, c). Cercles indicate the location of µ-RS (straight line) and EDXRF (dotted line) analyses; b: macro photo of one of the facets. Yellow arrows indicate traces of modification; c: macro photo of the raw surface of the sample; d: micro photo of the 3 analysed spots; e: Raman spectra of the 3 analysed spots; f: EDXRF spectrum obtained from sample.

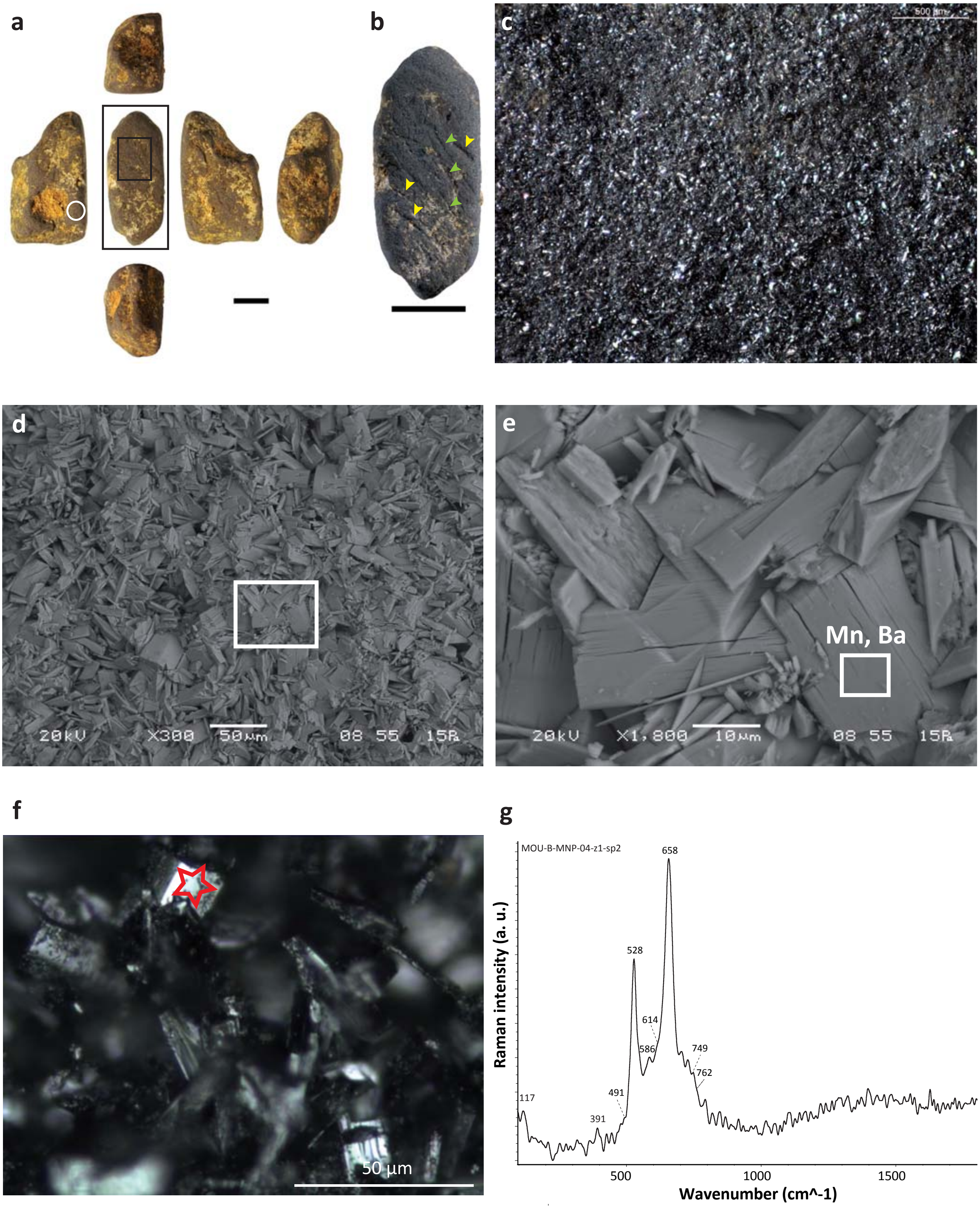

Figure B. Results of analyses conducted on MOU-MNP-04. a: photo of the lump. Squares indicate the position of macro photos (b, c). Cercle indicates the location of SEM-EDS and µ-RS analyses; b: macro photo of one of the facets displaying traces of modification; c: macro photo of the raw surface of the sample; d, e: SEM images in SE mode with results of EDS analyses; f: micro photo of the surface of the sample. Star indicates the analysed spot by µ-RS; g: Raman spectrum showing the main bands of pyrolusite.

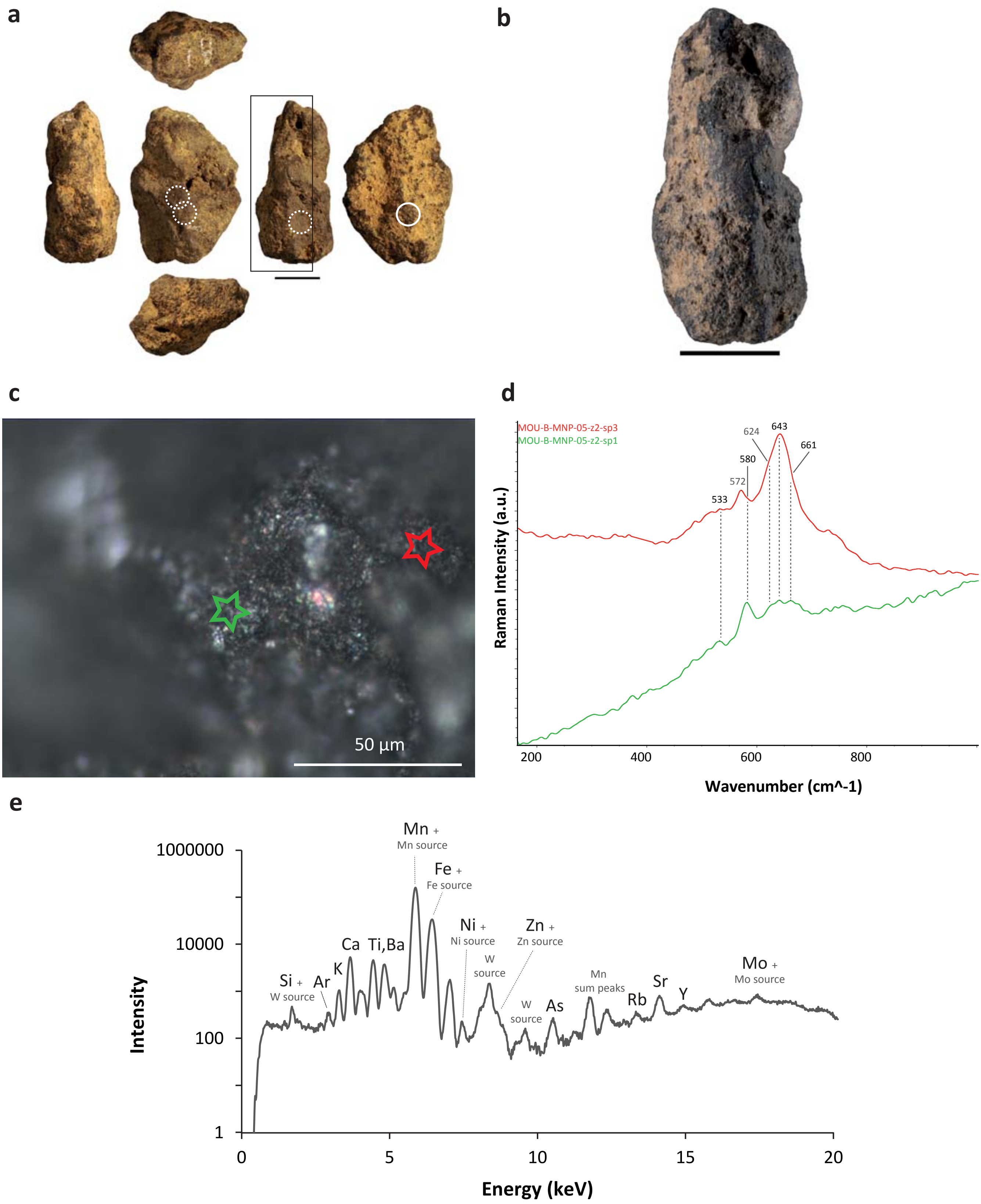

Figure C. Results of analyses conducted on MOU-MNP-05. a: photo of the lump. Square indicates the position of detailed photo of one of the facets (b). Cercles indicate the location of the µ-RS (straight line) and EDXRF (dotted line) analyses; b: macro photo of one of the facets of the sample; c: micro photo of the studied area. Stars indicate the analysed spots by µ-RS; d: Raman spectra of the 2 spots showing the main bands of pyrolusite, pyrochroite, and hollandite; e: EDXRF spectrum obtained from sample.

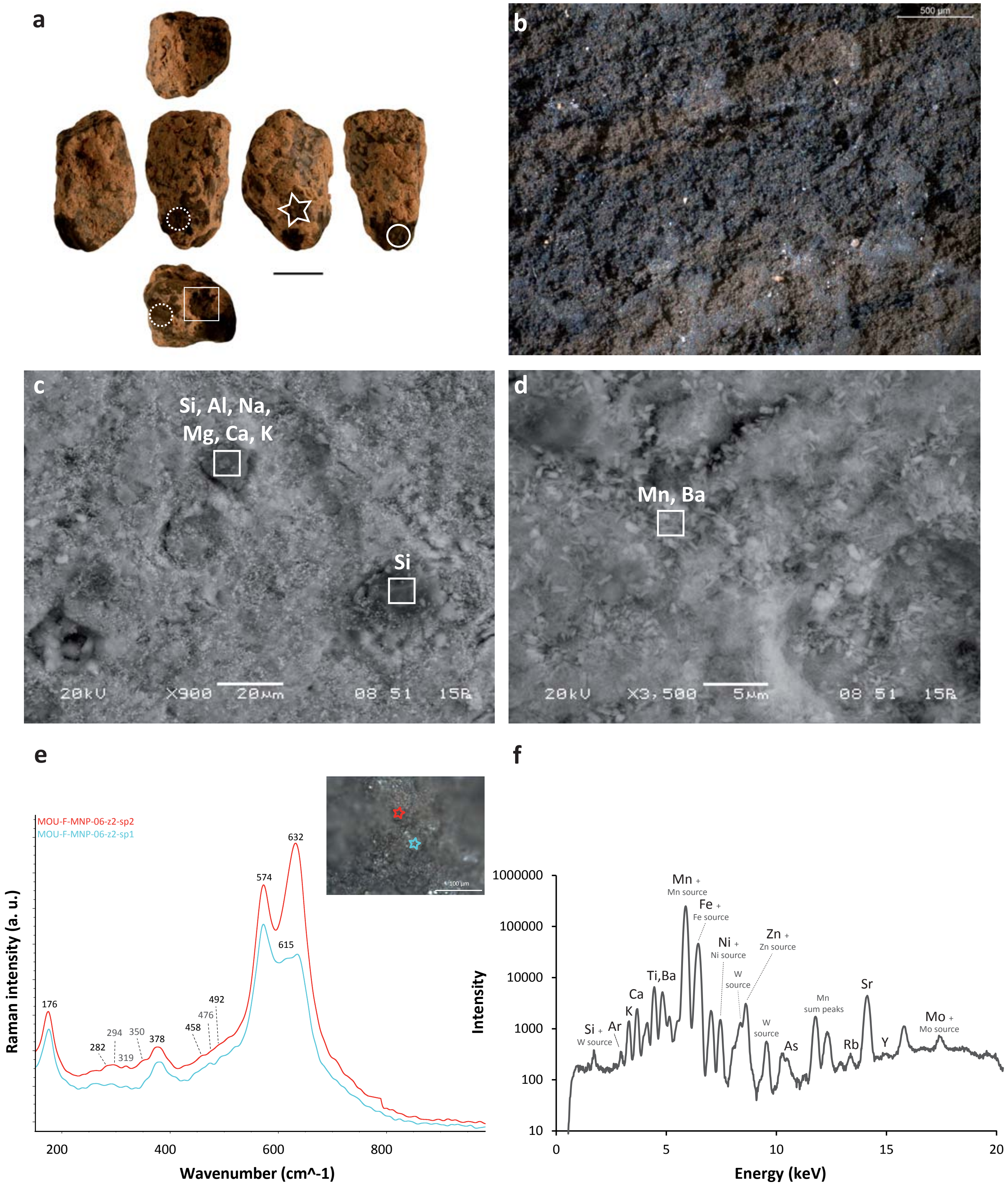

Figure D. Results of analyses conducted on MOU-MNP-06. a: photo of the lump. Square and star indicate the position of macro photo (b) and of SEM-EDS analyses (c, d). Cercles indicate the location of the µ-RS (straight line) and the EDXRF (dotted line) analyses; b: macro photo of the raw surface of the sample; c, d: SEM images in SE mode with results of EDS analyses; e: Raman spectra and micro photo of the analysed spots; f: EDXRF spectrum obtained from sample.

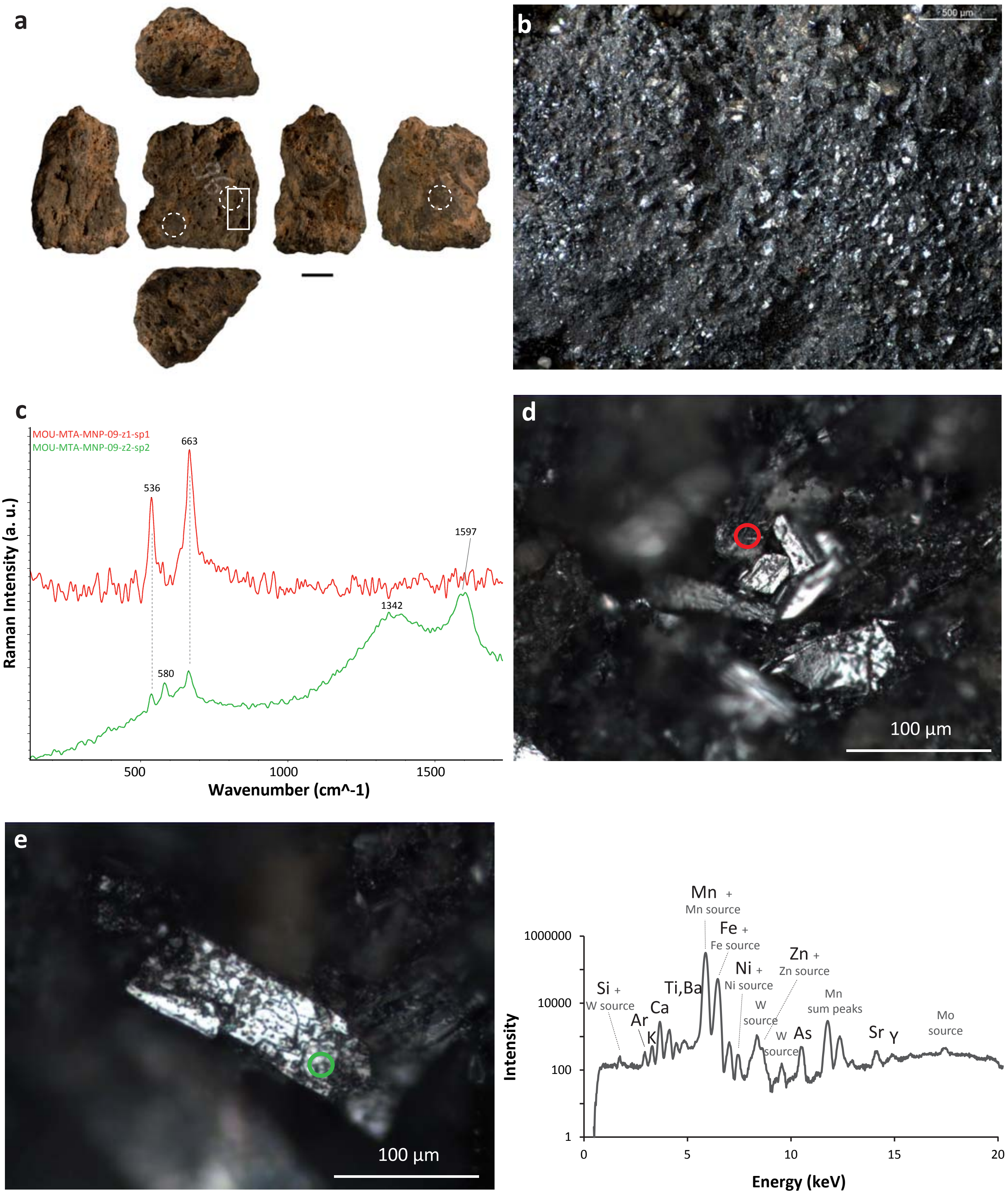

Figure E. Results of analyses conducted on MOU-MTA-MNP-09. a: photo of the sample. Square indicates the position of macro photo (b). Dotted cercles indicate the analysed areas by EDXRF (e); b, c: micro photos of the surface of the sample. Cercles indicate the analysed spots by µ-RS; d: Raman spectra of the 2 points showing the main bands of pyrolusite (red spectrum) and pyrolusite, hollandite, and carbon (green spectrum); e: EDXRF spectrum obtained from sample.

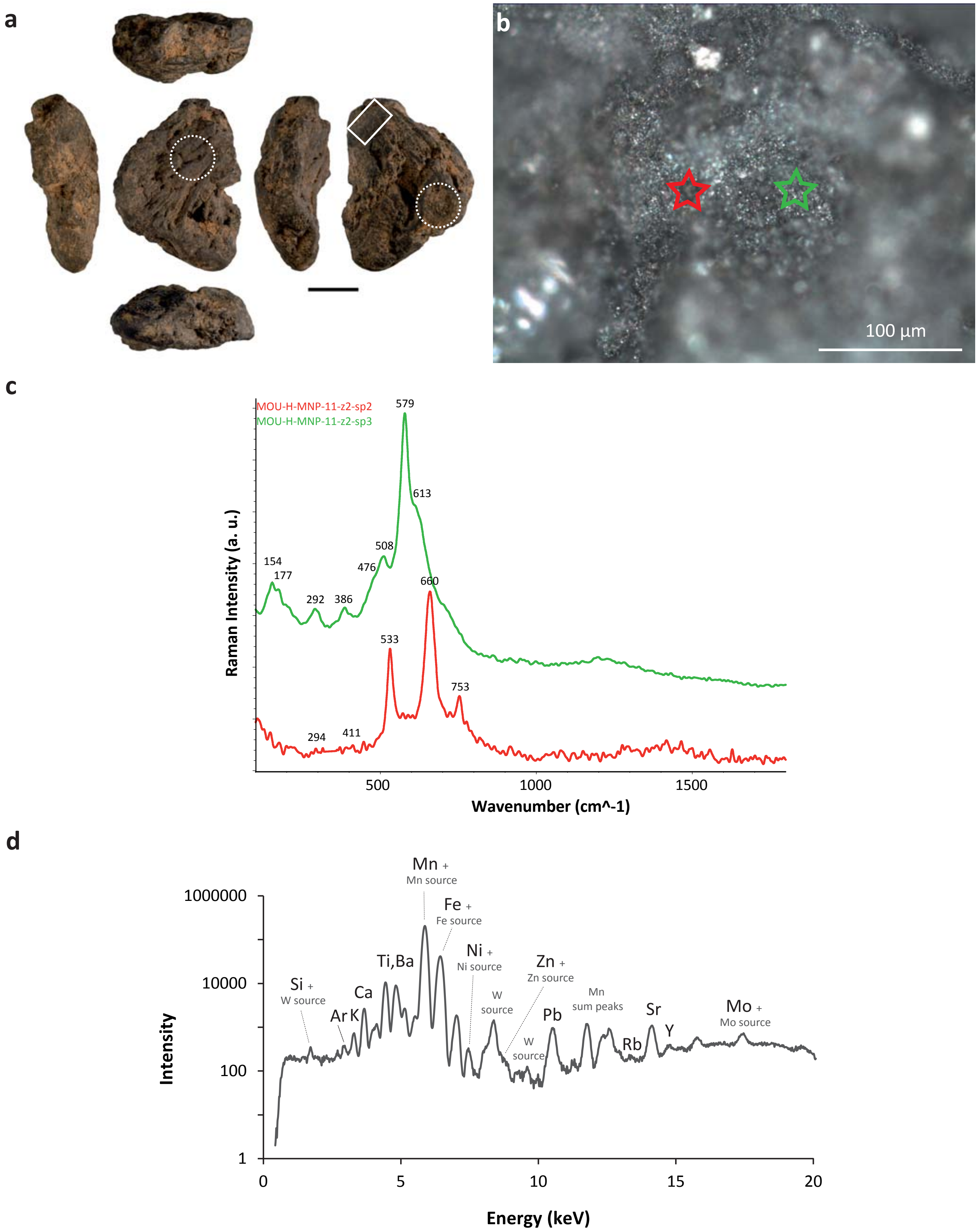

Figure F. Results of analyses conducted on MOU-H-MNP-11. a: photo of the lump. Square indicates the position of micro photo (b). Dotted-line cercles indicate the location of EDXRF analyses (d); b: micro photo of the surface of the sample. Stars indicate the analysed spots by µ-RS; c: Raman spectra of the 2 spots showing the heterogeneity of the sample; d: EDXRF spectrum obtained from sample.

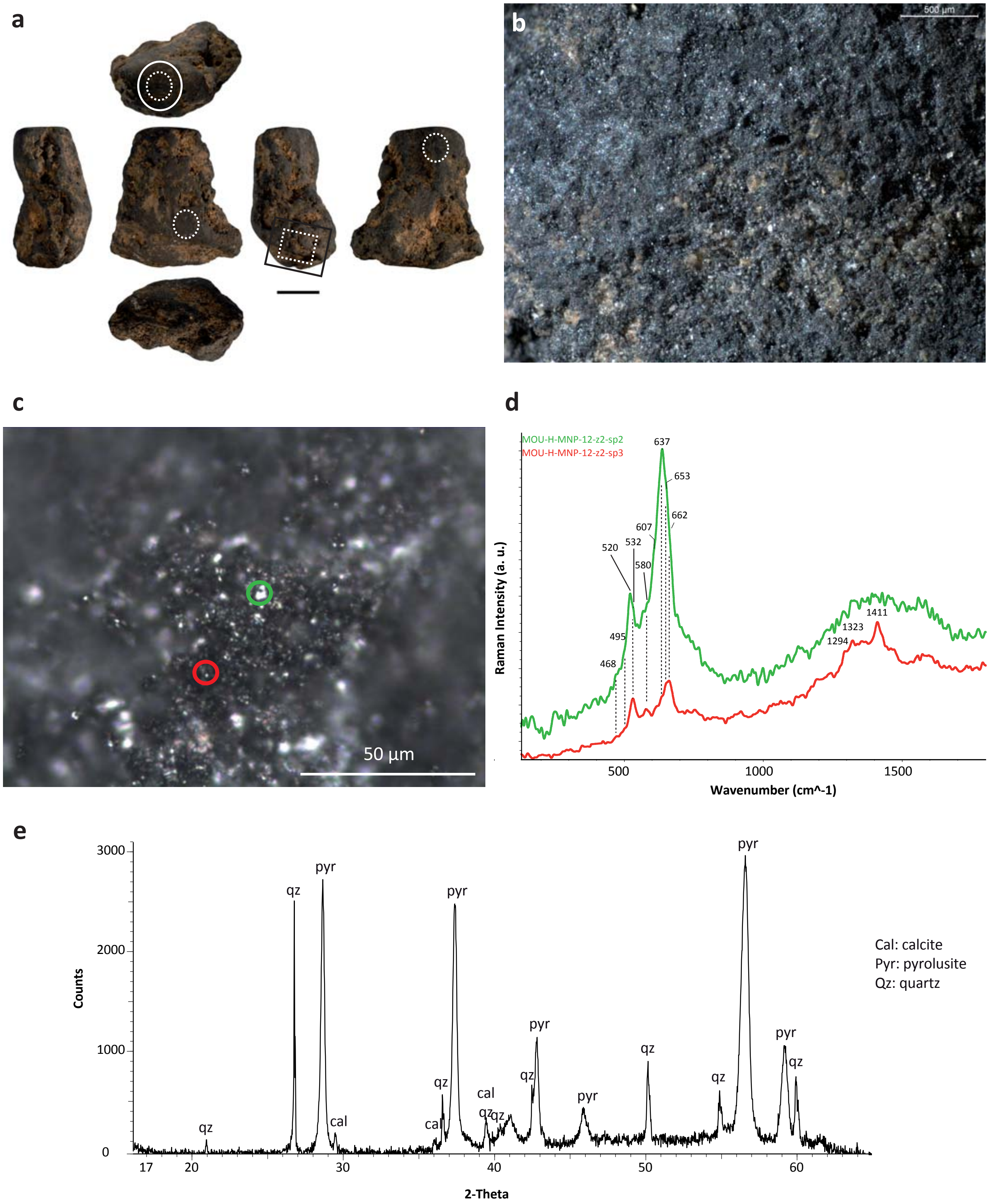

Figure G. Results of analyses conducted on MOU-H-MNP-12. a: photo of the lump. Squares indicate the position of macro photo (in black, b) and of µ-RS analyses (in white, c, d). Cercles show the location of EDXRF (dotted line, not shown) and XRD (straight line, e) analyses; b: macro photo of the surface of the sample; c: micro photo of the studied surface by µ-RS; d: Raman spectra of the 2 showing the main bands of todorokite, pyrolusite, hollandite, and the possible presence of alumino-silicates. Cercles indicate the analysed spots by µ-RS; e: XRD spectrum obtained from sample.

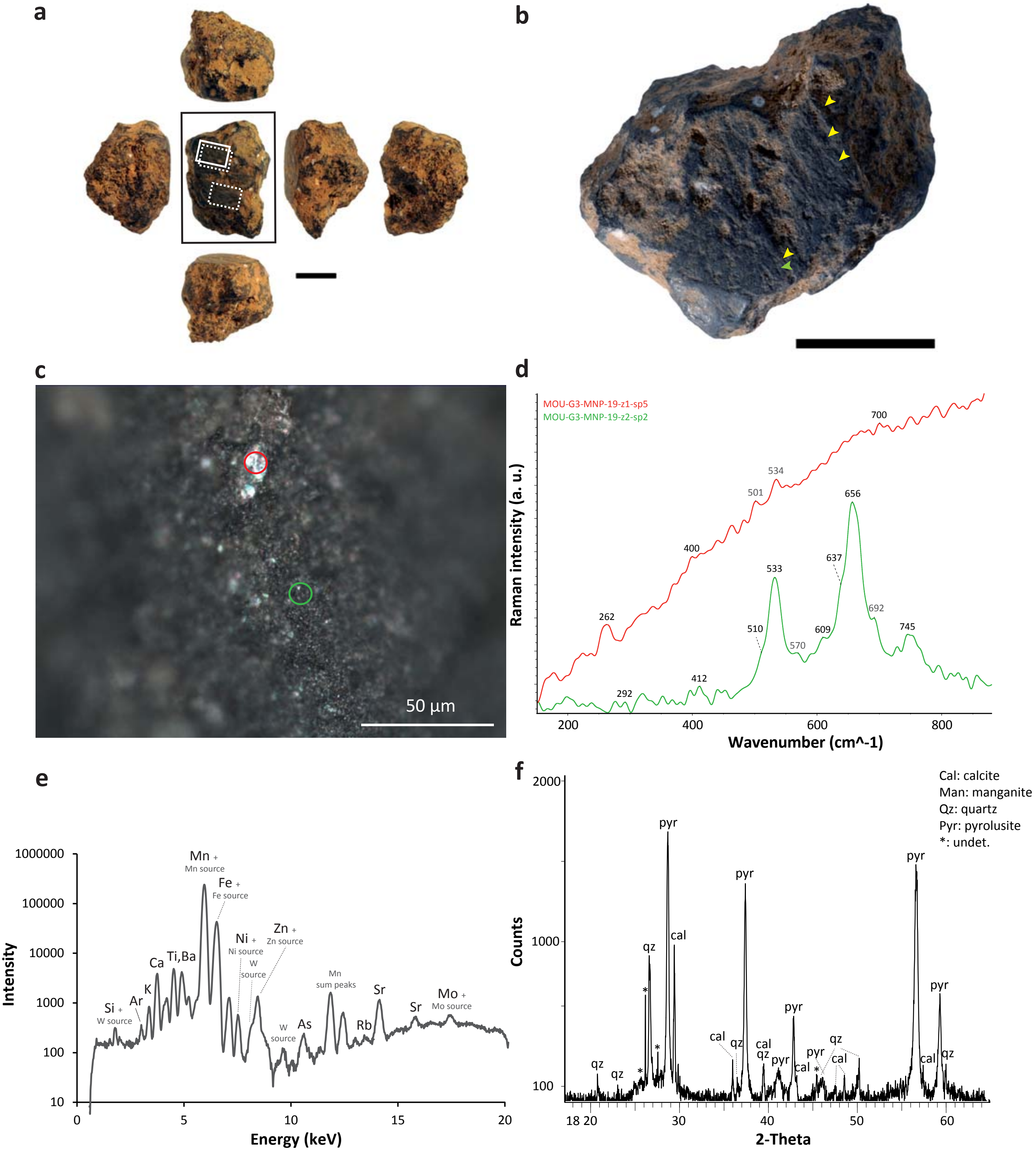

Figure H. Results of analyses conducted on MOU-G3-MNP-19. a: photo of the lump. Black square indicates the position of macro photo of the facet (b) while white squares indicate the location of analyses (dotted line - µ-RS (c, e); straight line - XRD) (d); b: macro photo of the facet; c: micro photo of the surface of the sample. Cercles indicate the analysed spots by µ-RS; d: Raman spectra of the 2 spots showing the main bands of muscovite (red spectrum) and pyrolusite (green spectrum), amongst other mineral phases; e, f: EDXRF and XRD spectra obtained from sample.

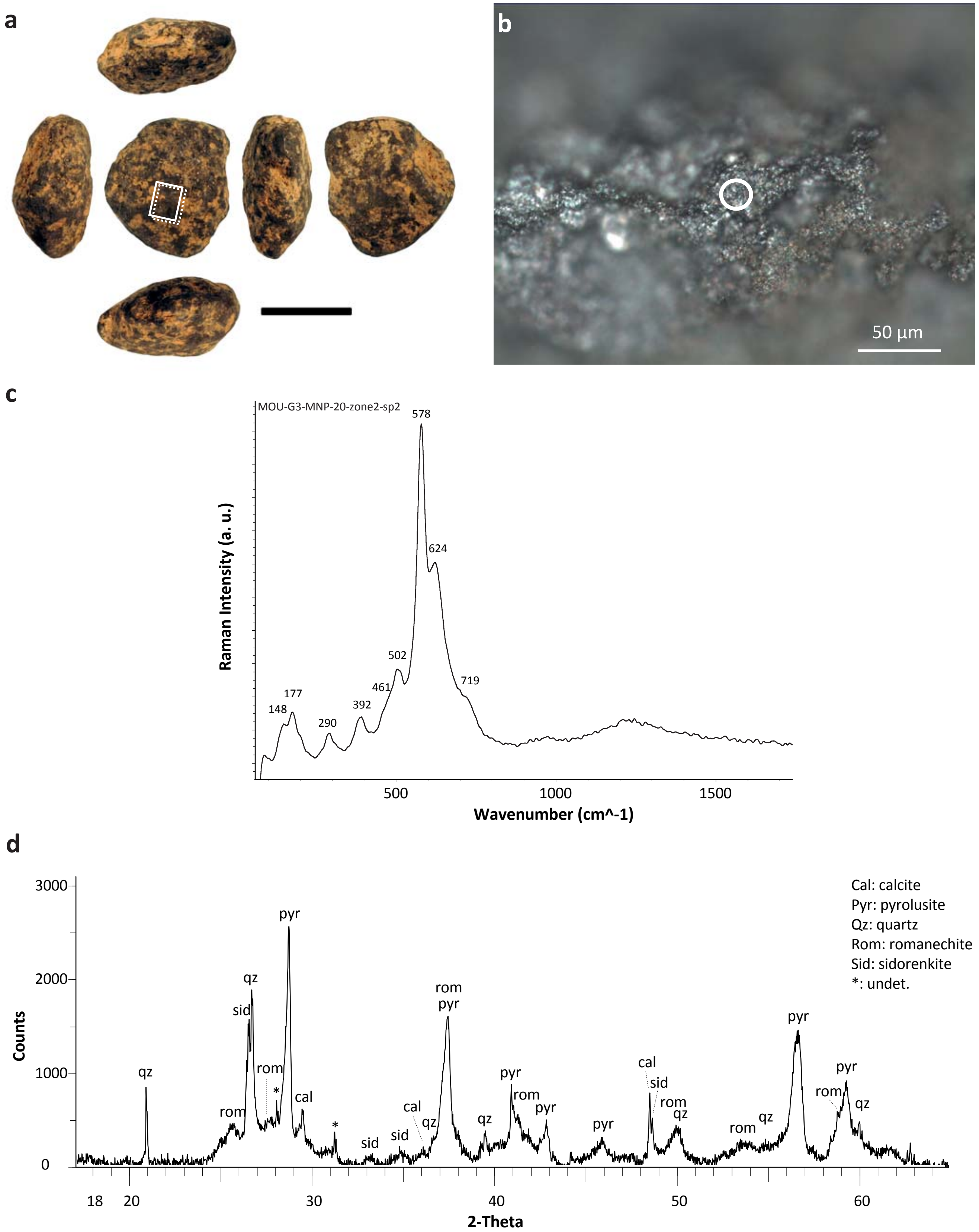

Figure I. Results of analyses conducted on MOU-G3-MNP-20. a: photo of the lump. Squares indicate the position of micro photo (dotted line) and Raman and XRD analyses (white straight line); b: micro photo of the surface of the sample. Cercle indicates the analysed spot by µ-RS; c: Raman spectrum of the spot showing the main bands of hollandite; d: XRD spectrum obtained from sample.

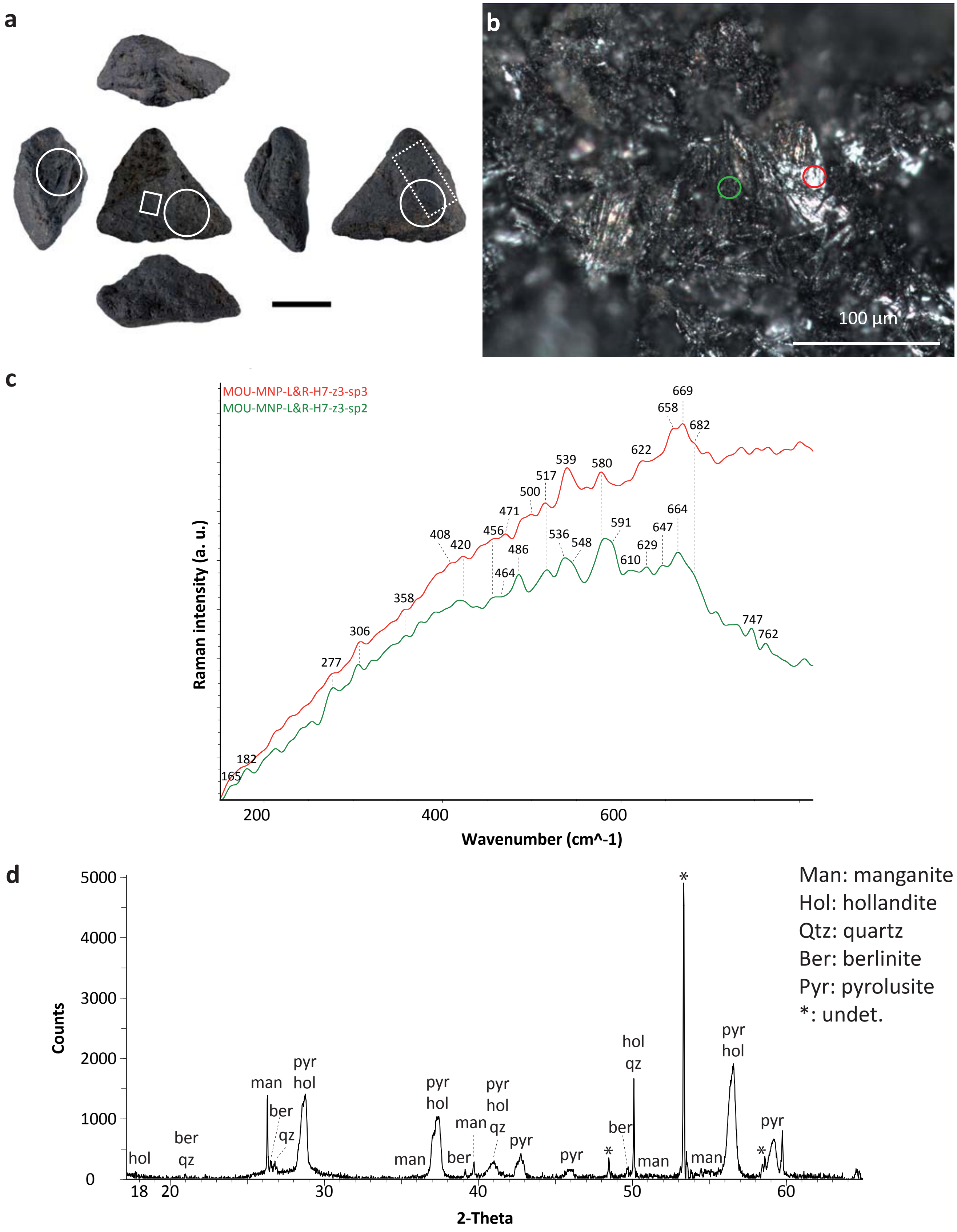

Figure J. Results of analyses conducted on MOU-MNP-L&R-H7. a: photo of the sample. Squares indicate the position of µ-RS and XRD analyses - straight and dotted line - while cercles indicate EDXRF measurements (not shown); b: micro photo of the surface of the sample. Cercles indicate the analysed spots; c: Raman spectra of the 2 spots by µ-RS; d: X-ray diffractogram obtained from sample.

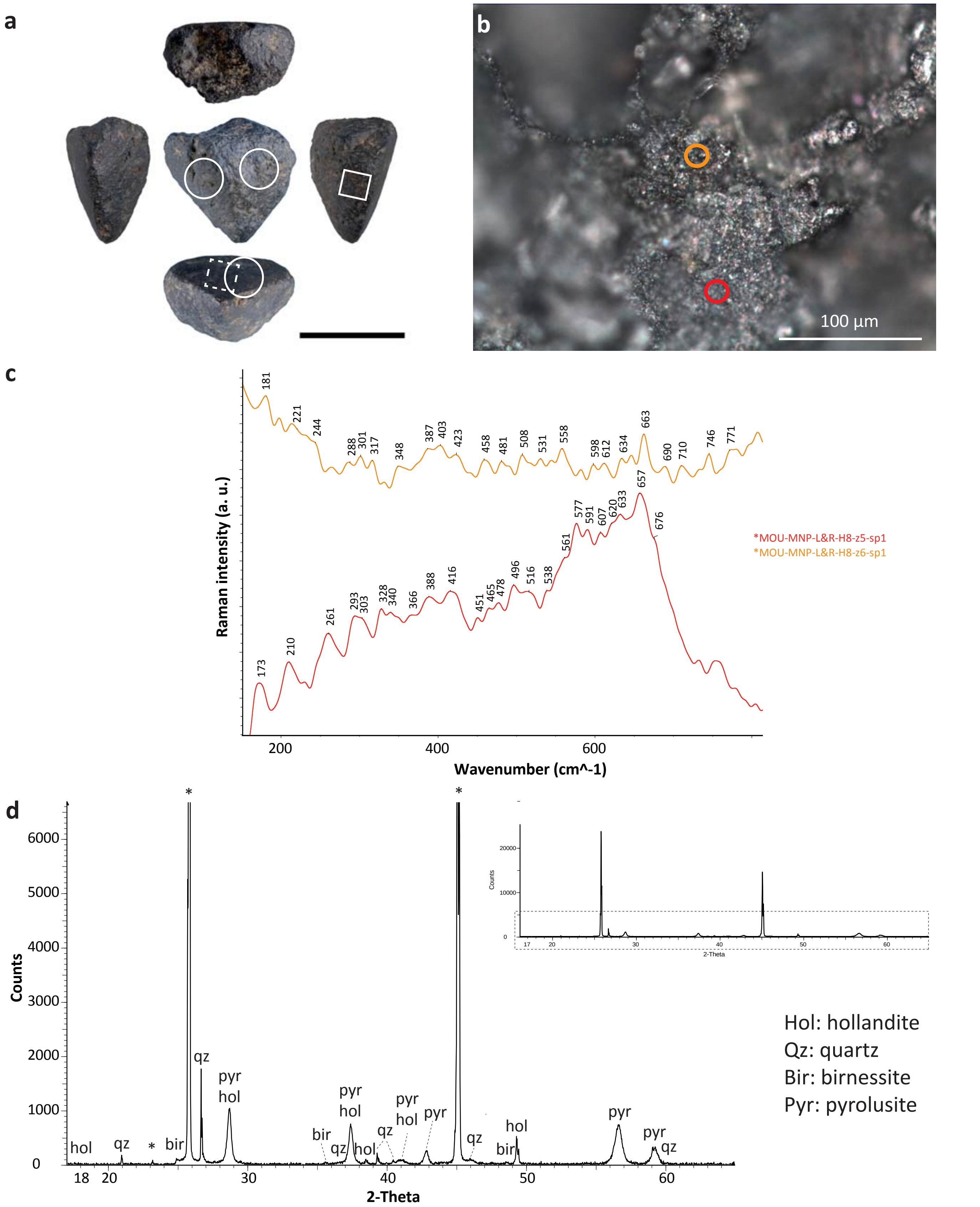

Figure K. Results of analyses conducted on MOU-MNP-L&R-H8. a: photo of the lump. Squares indicate the position of µ-RS and XRD analyses - straight and dotted line - while cercles indicate EDXRF measurements (not shown); b: micro photo of the surface of the sample. Cercles indicate the analysed spots by µ-RS; c: Raman spectra of the 2 spots; d: X-ray diffractogram obtained from

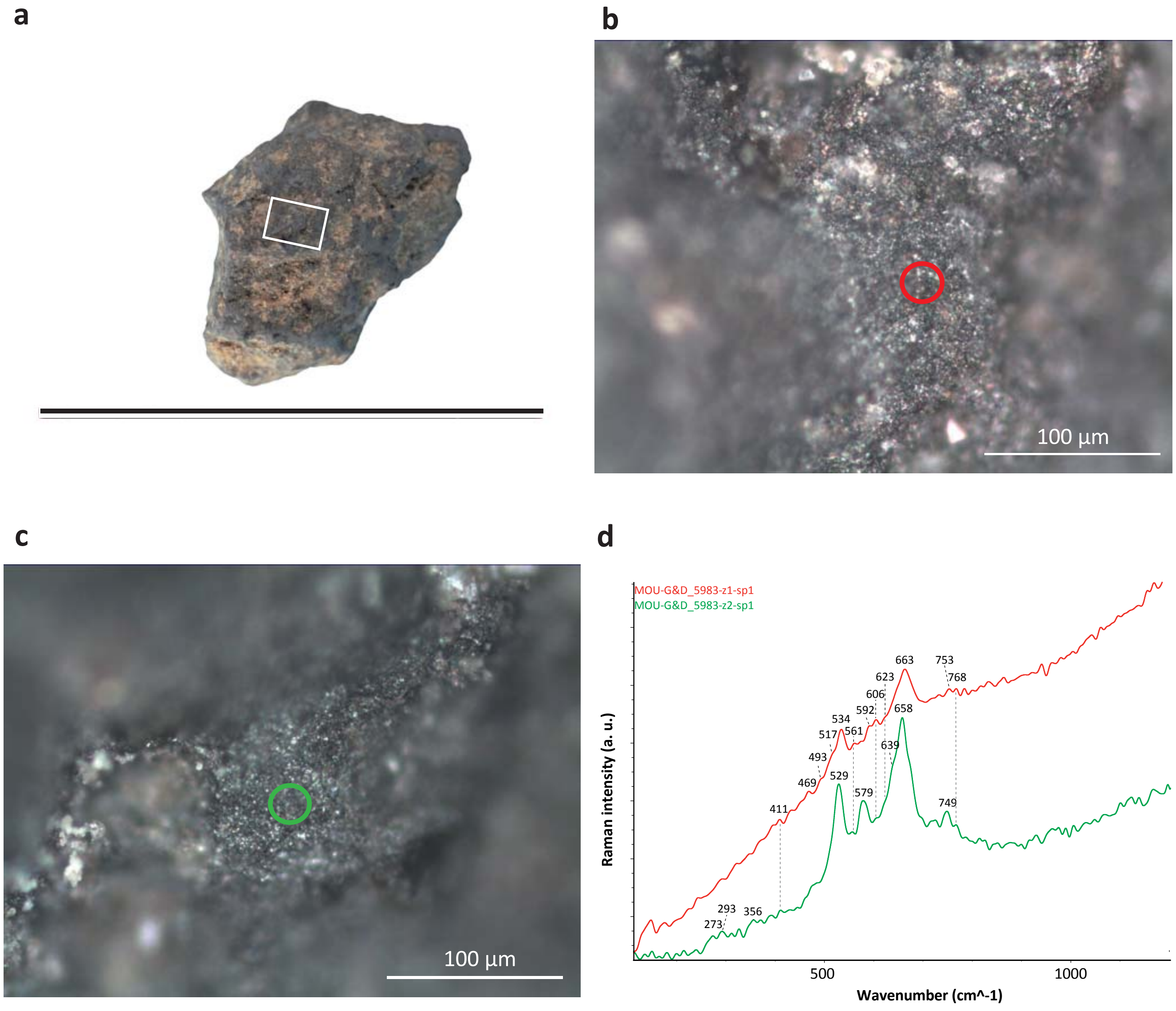

Figure L. Results of analyses conducted on MOU-G&D-5983. a: photo of the fragment. Square indicates the position of micro photos (b, c); b, c: micro photos of the raw surfaces of the sample. Cercles indicate the analysed spots by µ-RS; d: Raman spectra of the 2 spots.

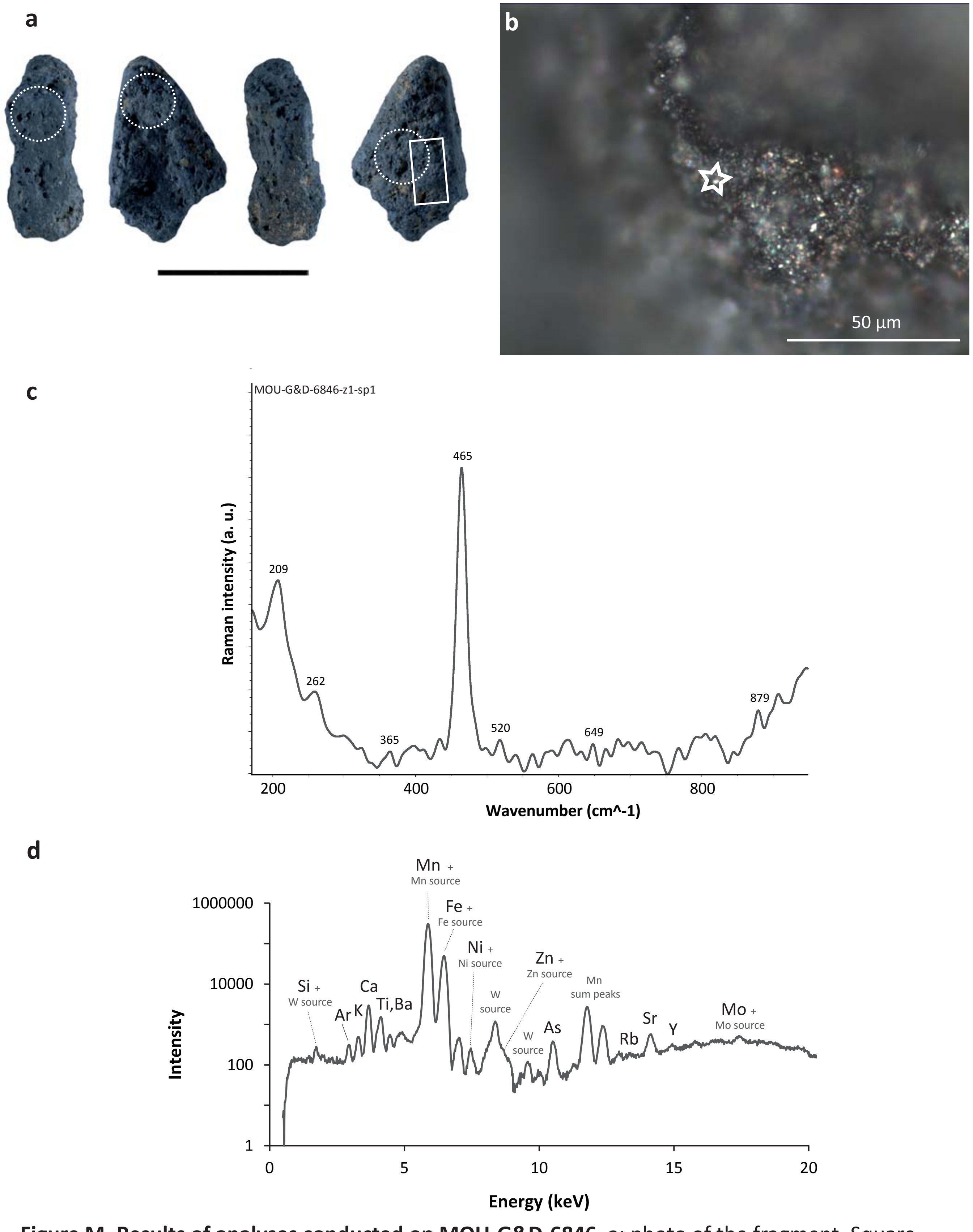

Figure M. Results of analyses conducted on MOU-G&D-6846. a: photo of the fragment. Square indicates the position of micro photo (b). Cercles indicate the location of the EDXRF analyses; b: micro photo of the surface of the sample. Star indicates the analysed spot; c: Raman spectrum of the spot; d: EDXRF spectrum obtained from sample.

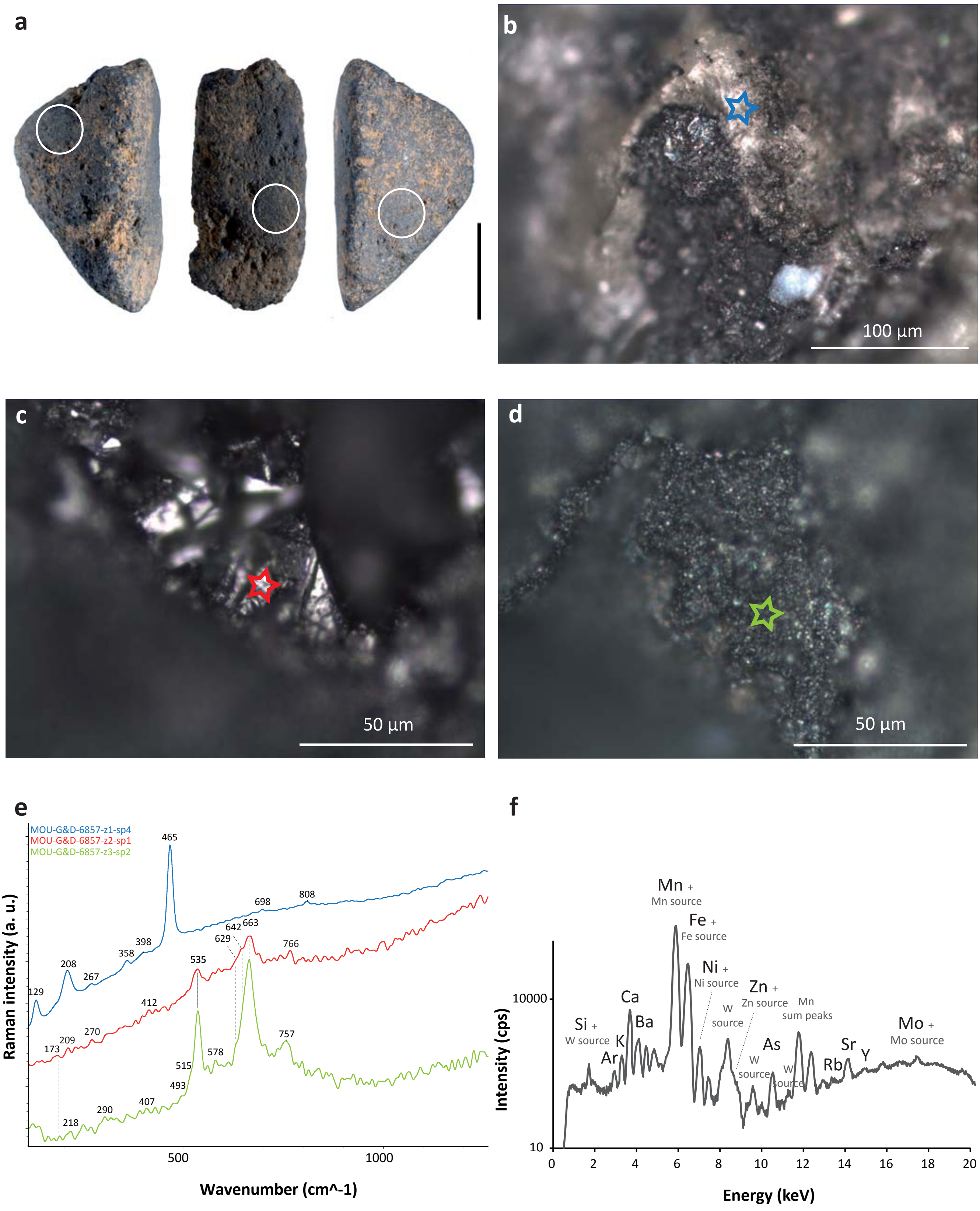

Figure N. Results of analyses conducted on MOU-G&D-6857. a: photo of photo of the lump. Cercles indicate the position of the EDXRF analyses; b, c, d: micro photos of the surface of the sample. Stars indicate the analysed spots by µ-RS; e: Raman spectra of the 3 spots showing the presence of pyrolusite, pyrochroite, and quartz; f: EDXRF spectrum obtained from sample.

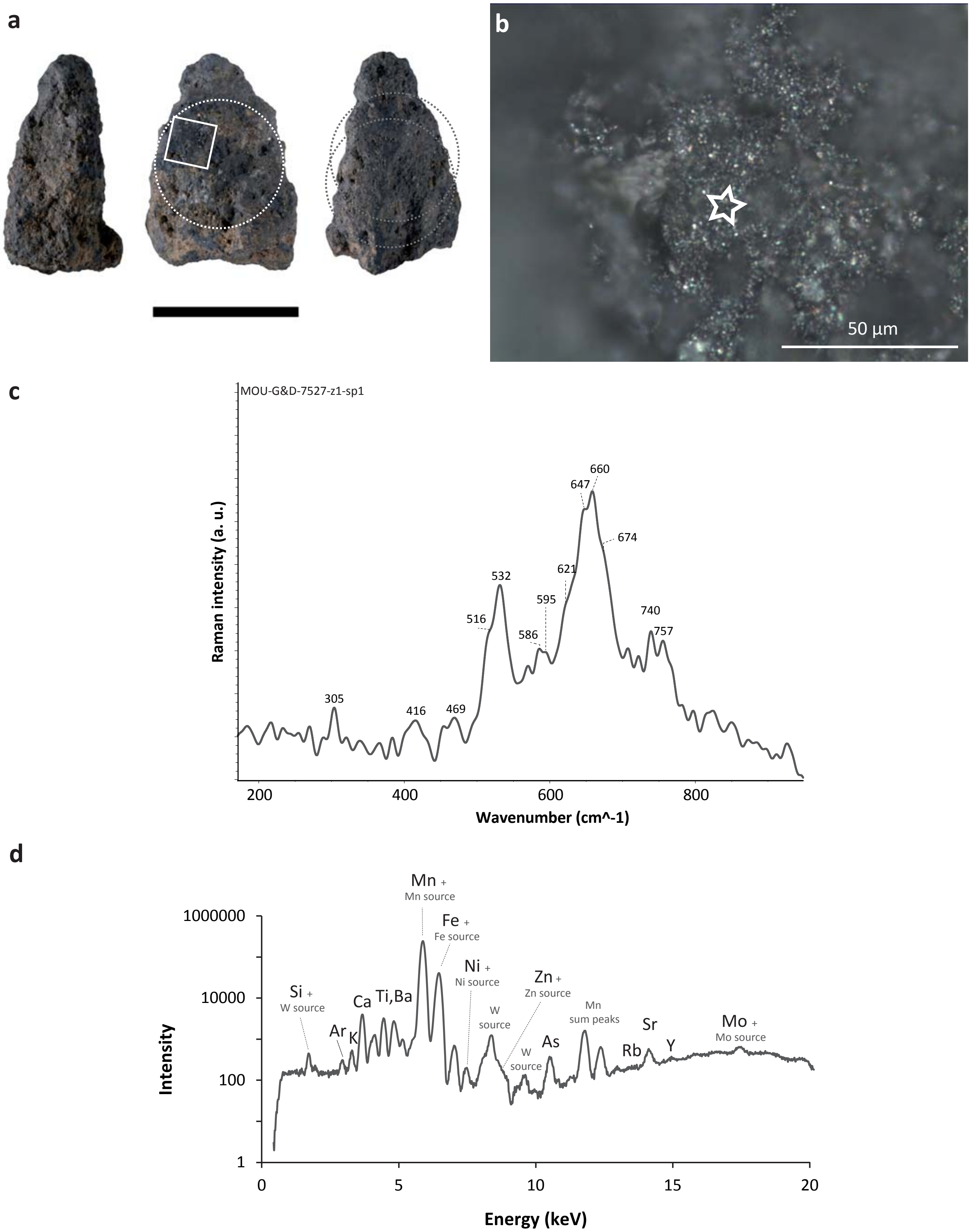

Figure O. Results of analyses conducted on MOU-G&D-7527. a: photo of the fragment. Square indicates the position of the micro photo (b). Cercles indicate the location of EDXRF analyses (d); b: micro photo of the surface of the sample. Star indicates the analysed spot by µ-RS; c: Raman spectrum of the spot showing the main bands of pyrolusite, pyrochroite, and holandite; d: EDXRF spectrum obtained from sample.

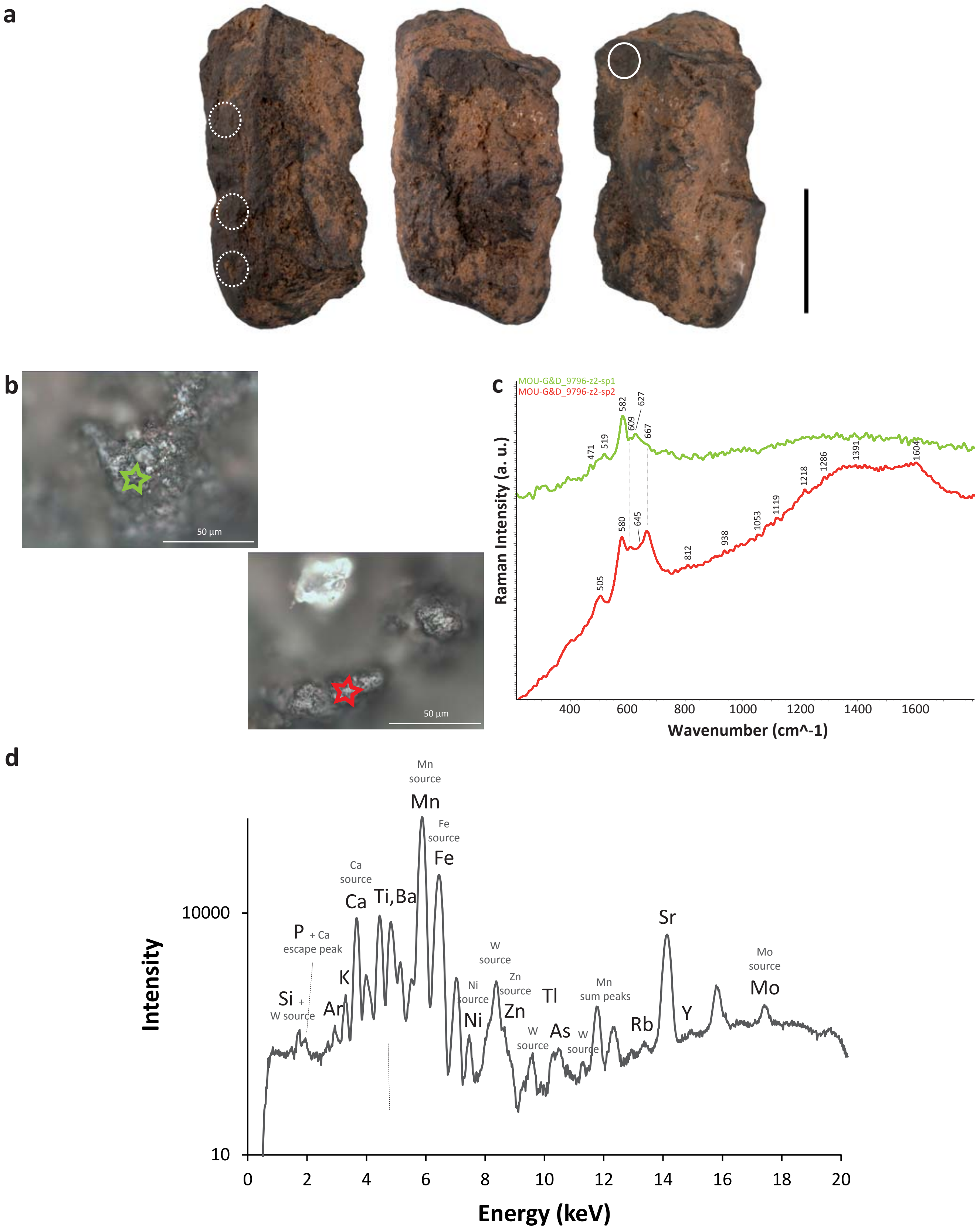

Figure P. Results of analyses conducted on MOU-G&D-9796. a: photo of the object. Cercles indicate the location of µ-RS (straight line) and EDXRF (dotted line) analyses; b, c: microscopic images and Raman spectra of the spots showing the main bands of hollandite, pyrochroite, pyrolusite, and todorokite amongst other mineral phases; d: EDXRF spectrum obtained from sample.

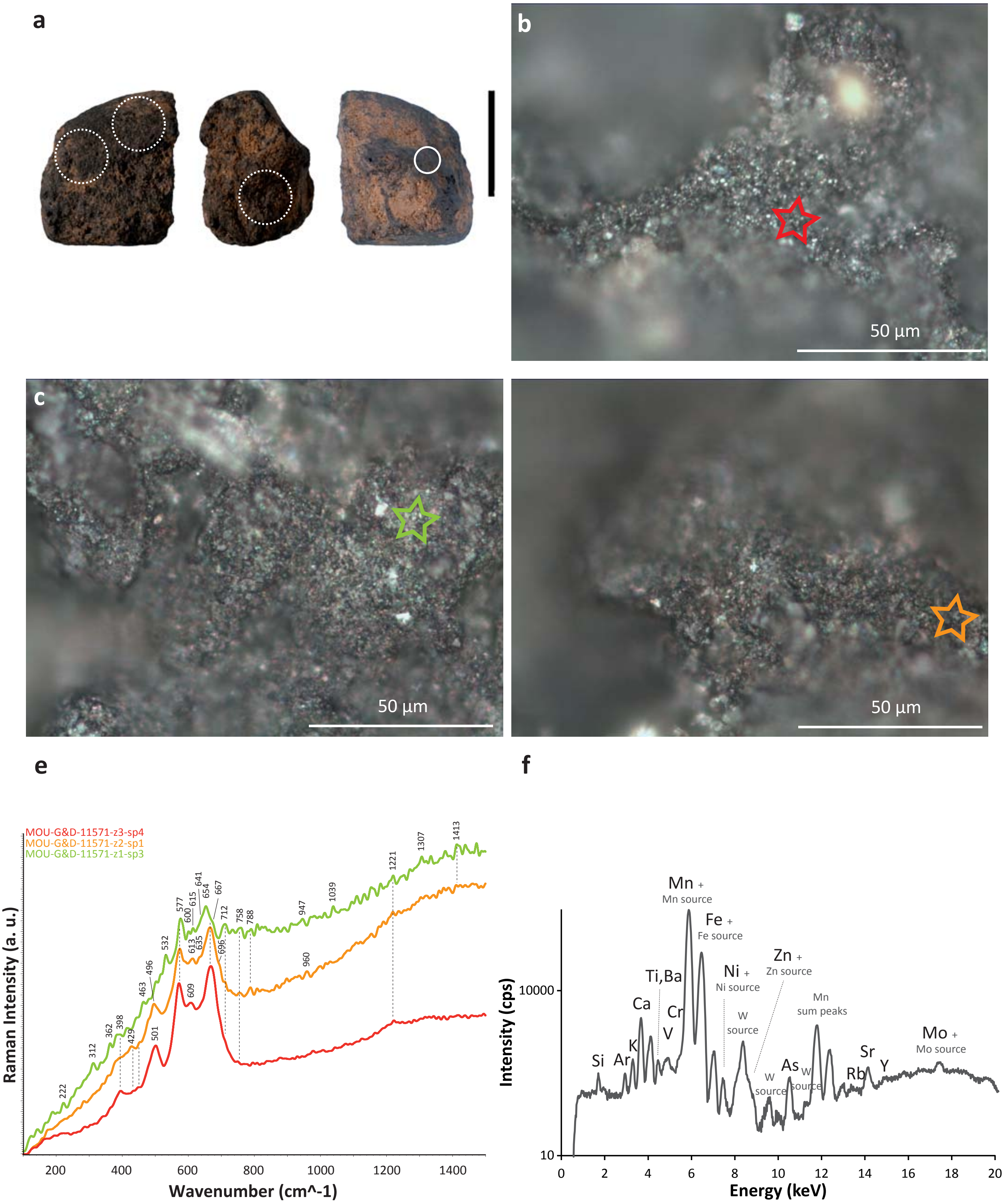

Figure Q. Results of analyses conducted on MOU-G&D-11571. a: photo of the lump. Cercles indicate the location of µ-RS (straight line) and EDXRF (dotted line) analyses; b, c, d: micro photos of the surface of the sample. Stars indicate the analysed spots by µ-RS; e: Raman spectra of the three spots showing the main bands of hollandite and pyrolusite, amongst others; f: EDXRF spectrum obtained from sample.
