# Supplementary material for: Provenance, modification and use of manganese-rich rocks at Le Moustier (Dordogne, France)
Source: PLoS One. 2019 Jul 17;14(7):e0218568. doi: 10.1371/journal.pone.0218568 (PMC6636720; doi:10.1371/journal.pone.0218568)
Supplement: S3 Fig — (PDF) [file pone.0218568.s004.pdf]

Provenance, modification and use of manganese-rich rocks at Le Moustier, Dordogne, France

Africa Pitarch Martí*, Francesco d'Errico, Alain Turq, Eric Lebraud, Emmanuel Discamps, Brad Gravina.

*Corresponding Author

E-mail: africa.pitarch@gmail.com (APM)

S3 Figures

Results of analyses conducted on Mn-rich geological samples from Dordogne and Lot regions of France

Figure A. Back-scattered electron images of representative areas from the geological samples collected in this study....................2

Figures B and C. Results of analyses conducted on samples from *Mine d'Albas*....................3-4

Figure D. Results of analyses conducted on samples from *Mine de Causse du Cluzel*....................5

Figures E and F. Results of analyses conducted on samples from *Mine de Sals*....................6-7

Figures G, H and I. Results of analyses conducted on samples from *Mine de Le Theil*..........8-10

Figure J. Results of analyses conducted on samples from *Grotte-Cave*....................11

Figure K. Results of analyses conducted on samples from *Grotte du Loup*....................12

Figure L. Results of analyses conducted on samples from *Grotte de Beyssac*....................13

Figures M, N and O. Results of analyses conducted on samples from *Grotte du Trou du Vent*....................14-16

Figure P. Results of analyses conducted on samples from *Sarlat*....................17

Figure Q. Results of analyses conducted on samples from *Carrière Le Verdier*....................18

Figure R. Results of analyses conducted on samples from *Teyjat*....................19

Figure S. Results of analyses conducted on samples from *Tranchecouyère*....................20

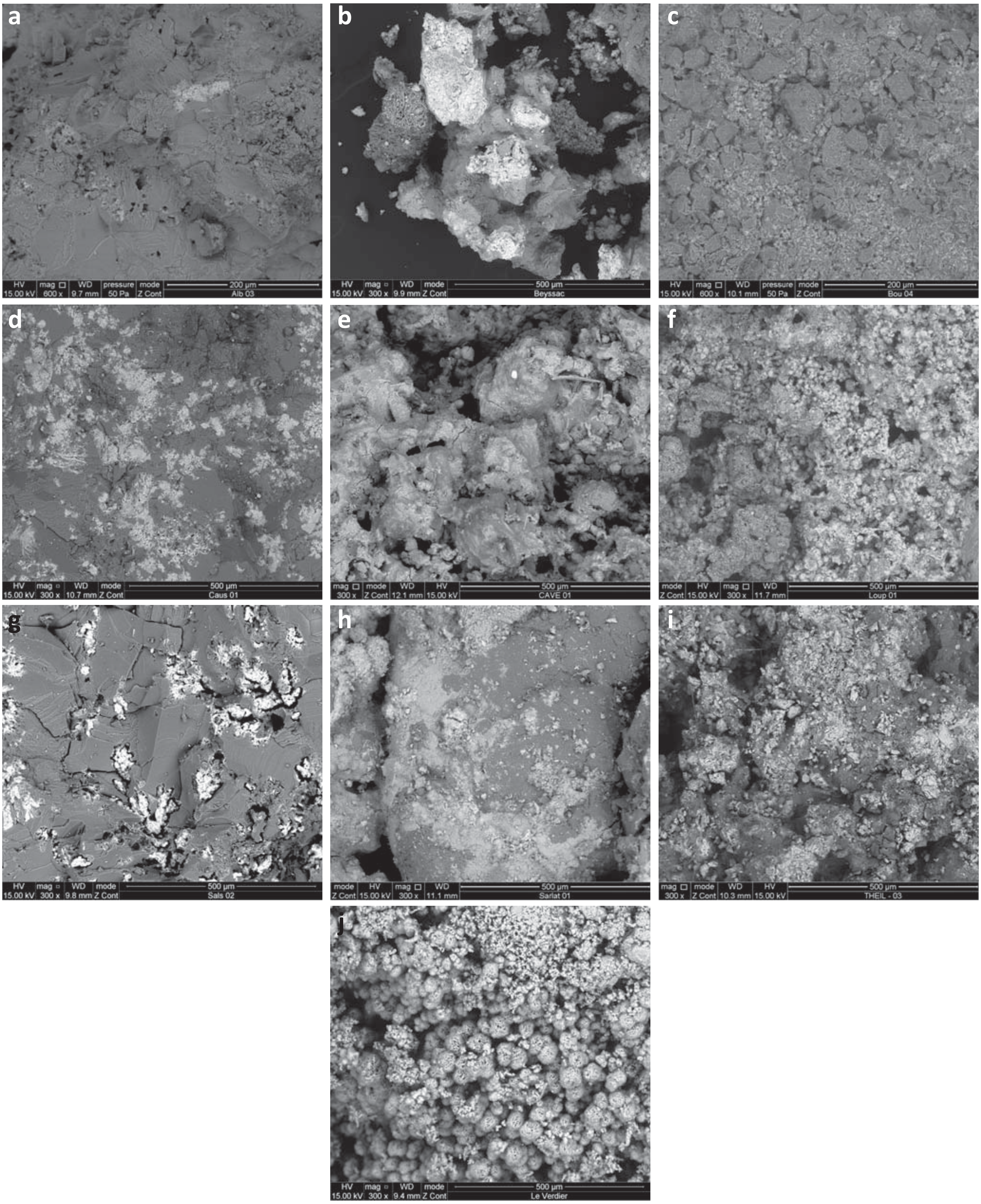

Figure A. SEM images in back-scattered electron mode of selected geological samples; a: *Mine d'Albas* (ALB-03); b: *Grotte de Beyssac* (BEY-01); c: *Grotte du Trou du Vent* (BOU-04); d: *Mine de Causse du Cluzel* (CAU-01); e: *Grotte-Cave* (CAV-01); f: *Grotte du Loup* (LOU-01); g: *Mine de Sals* (SAL-02); h: *Sarlat* (SAR-01); i: *Mine de Le Theil* (THE-03); j: *Carrière Le Verdier* (VER-01).

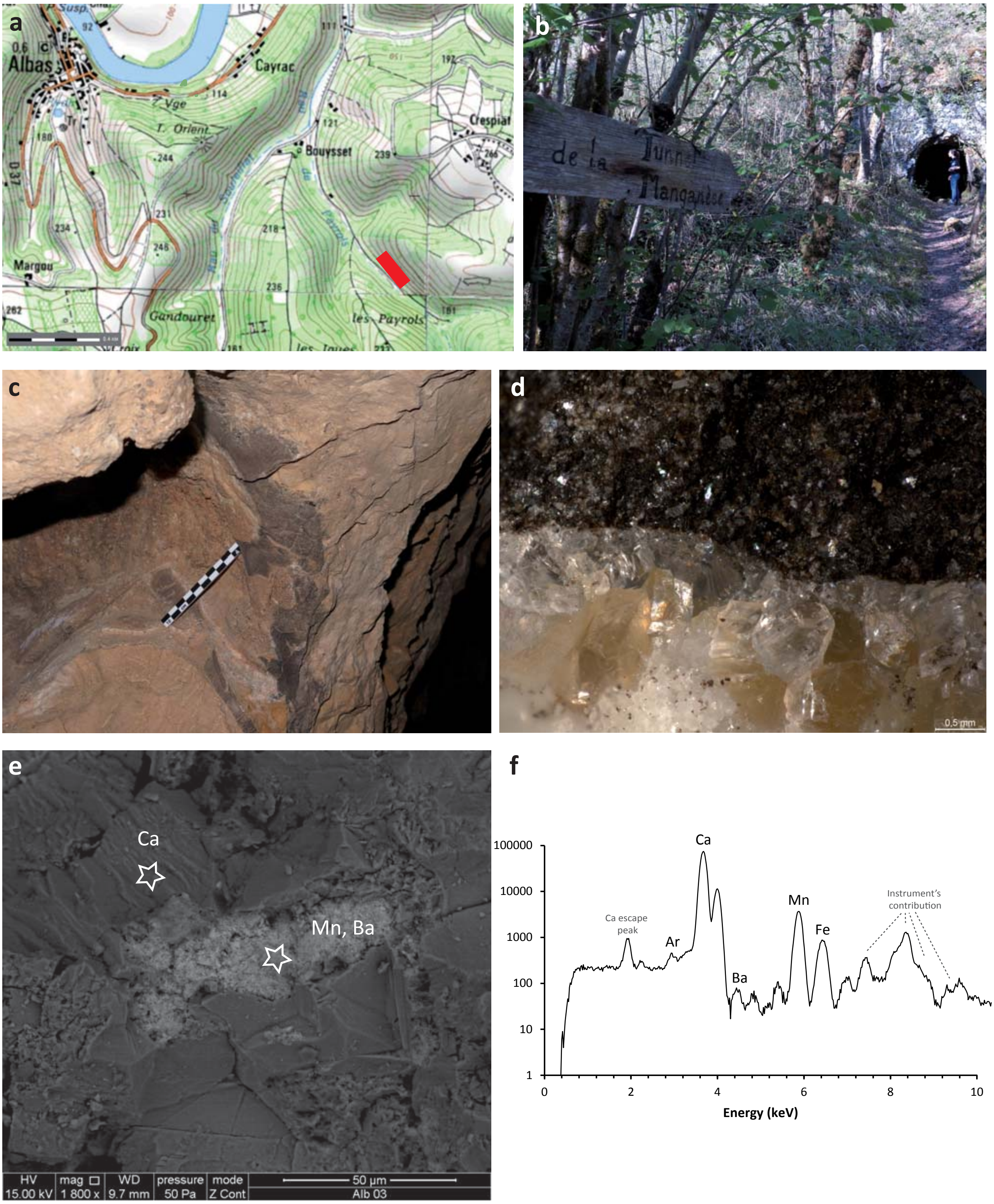

Figure B. Results of analyses conducted on samples from *Mine d'Albas*. a: location of the outcrop (in red); b: view of the entrance of the tunnel (@C. Magarzo); c: photo of the sampled area; d: macro photo of the surface of the sample ALB-03; e: SEM image in BSE mode; f: EDXRF obtained from pulverized sample.

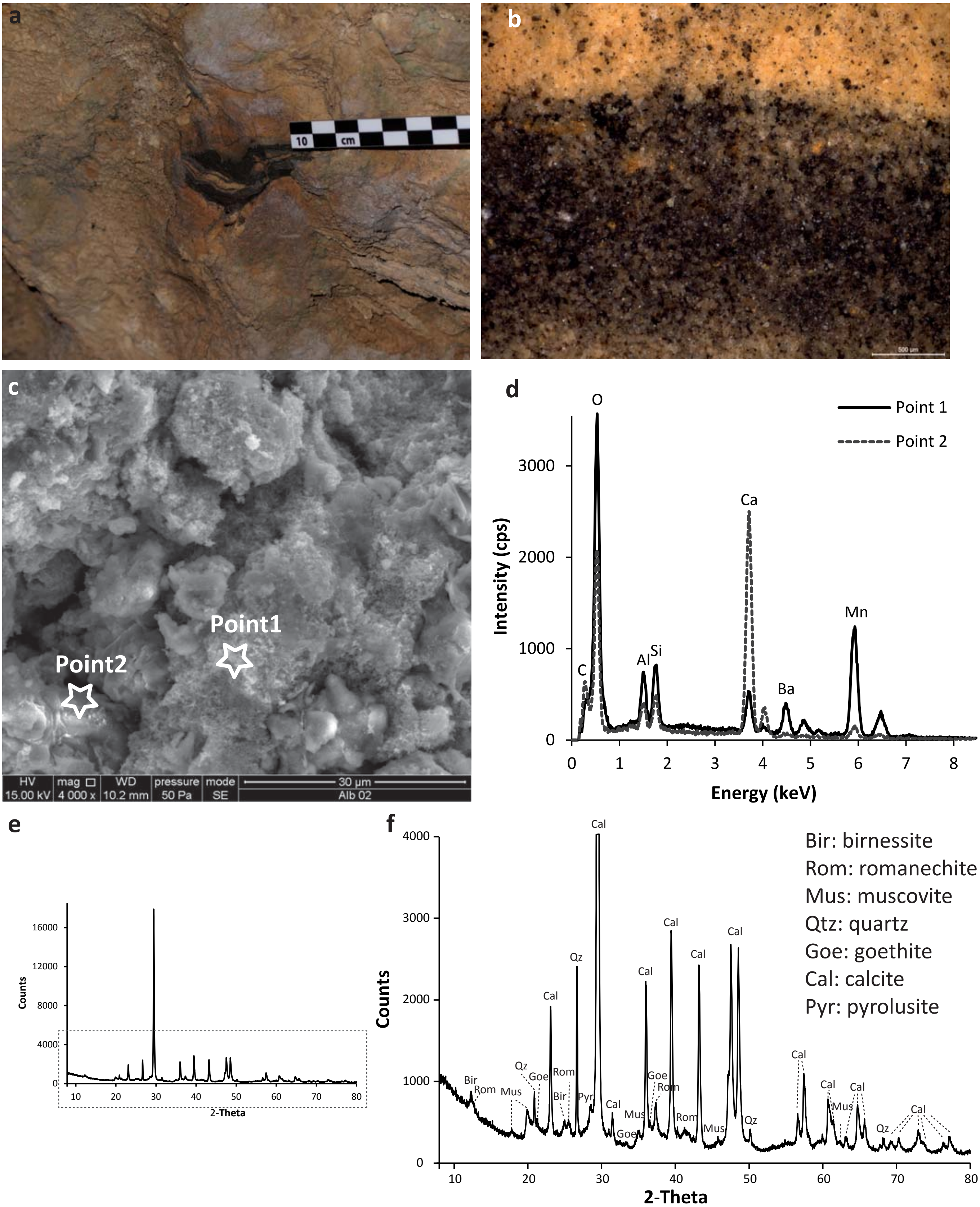

Figure C. Results of analyses conducted on samples from *Mine d'Albas*. a: photo of the sampled area (@C. Magarzo); b: macro photo of the surface of sample ALB-02; c: SEM image in SE mode of sample ALB-02 (stars indicate analysed spots); d: EDS spectra of spots 1 and 2 indicated in c; e, f: X-ray diffractogram obtained from pulverized sample ALB-01.

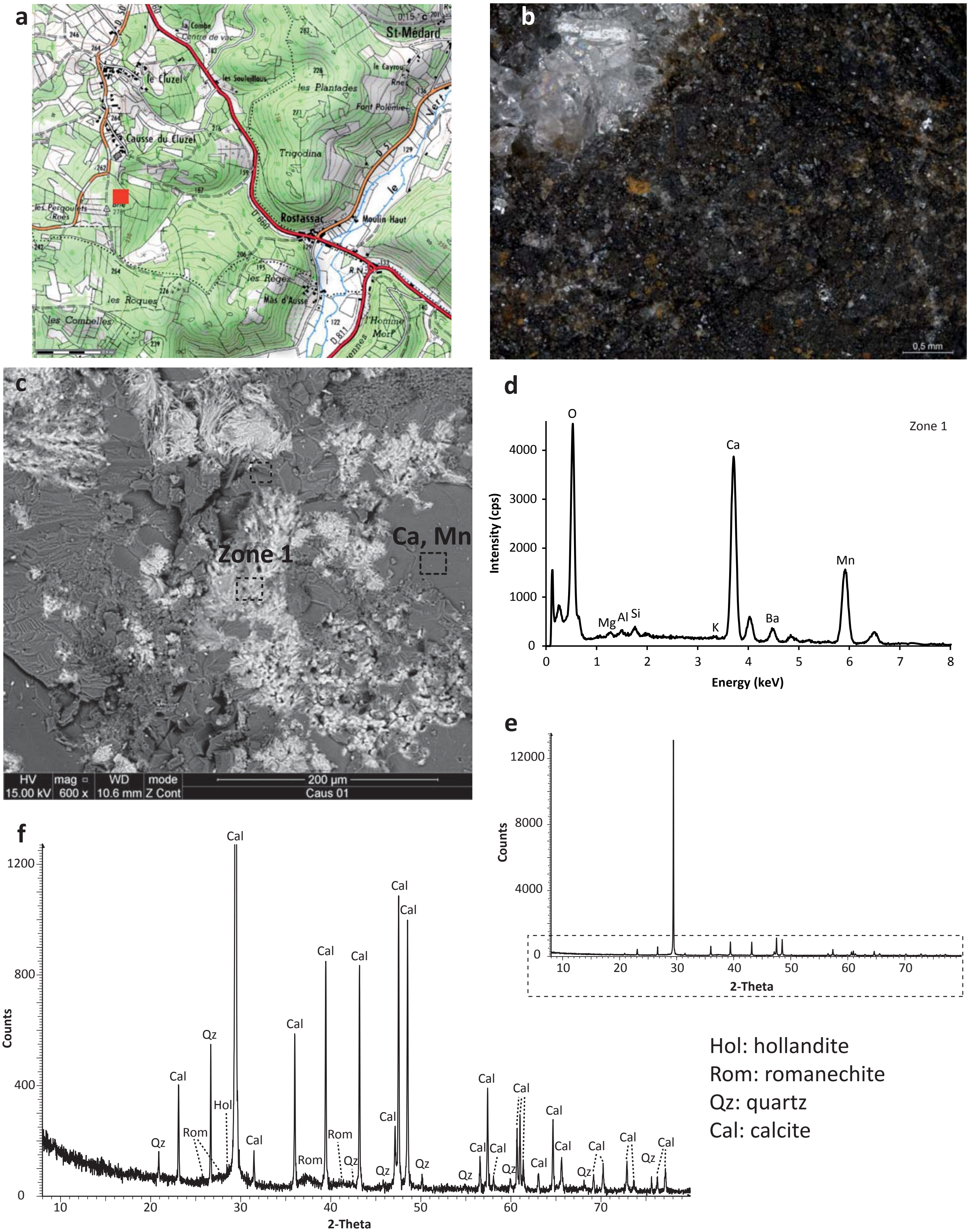

Figure D. Results of analyses conducted on samples from *Mine de Causse du Cluzel*. a: location of the outcrop (red square); b: macro photo of the surface of the sample CAU-01 ; c: SEM images in BSE mode of the sample (zones indicate analysed areas); d: EDS spectrum of zone 1 represented in c; e: X-ray diffractogram obtained from pulverized sample; f: zoom of the X-ray diffractogram represented in e.

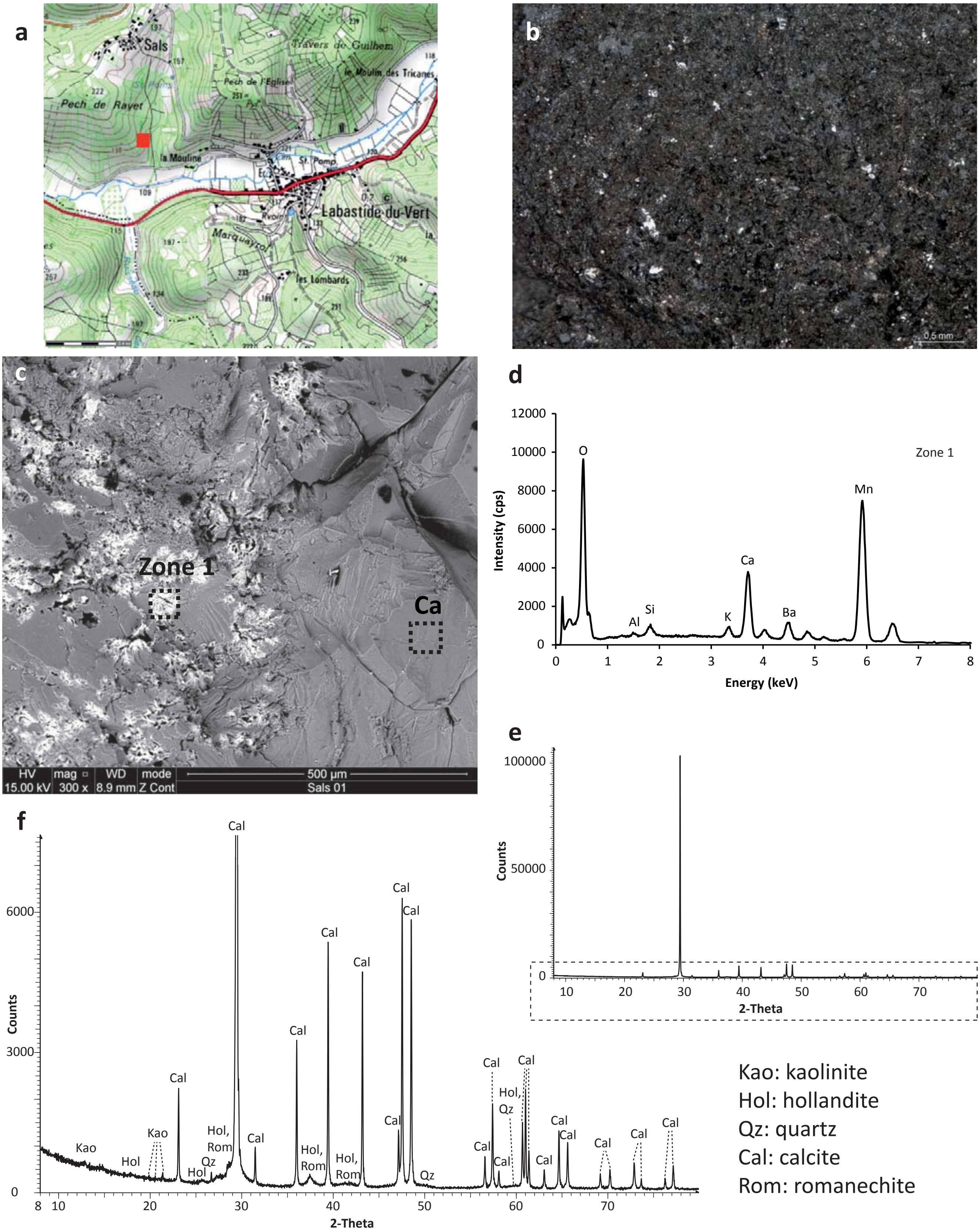

Figure E. Results of analyses conducted on samples from *Mine de Sals*. a: location of the outcrop (red square); b: macro photo of the surface of the sample SAL-01; c: SEM images in BSE mode of the sample (squares indicate analysed area); d: EDS spectrum of zone 1 represented in c; e: X-ray diffractogram obtained from pulverized sample; f: zoom of the X-ray diffractogram represented in e.

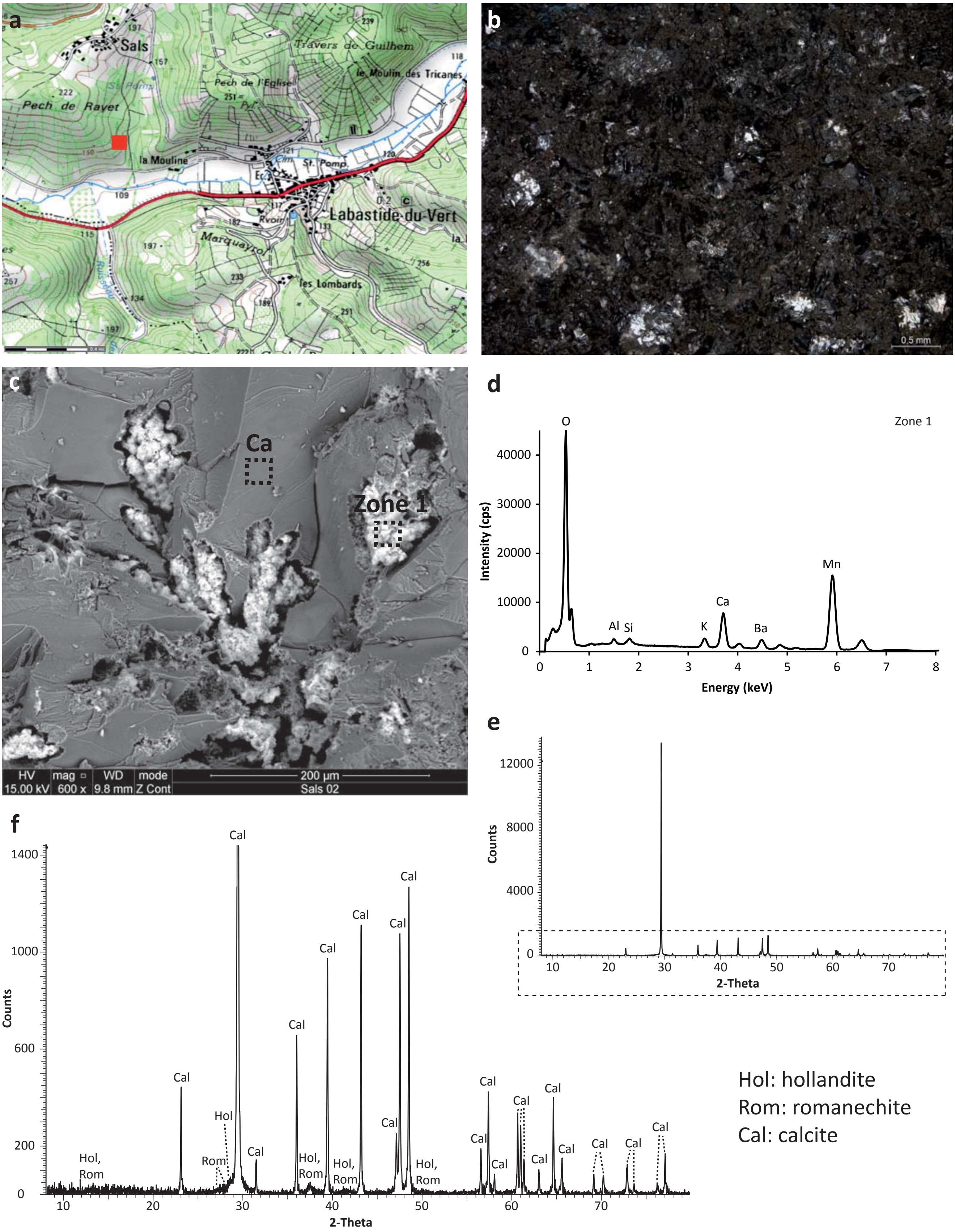

Figure F. Results of analyses conducted on samples from *Mine de Sals.* a: location of the outcrop (red square); b: macro photo of the surface of the sample SAL-02; c: SEM images in BSE mode of the sample (zones indicate analysed area); d: EDS spectrum of zone 1 represented in c; e: X-ray diffractogram obtained from pulverized sample; f: zoom of the x-ray diffractogram represented in e.

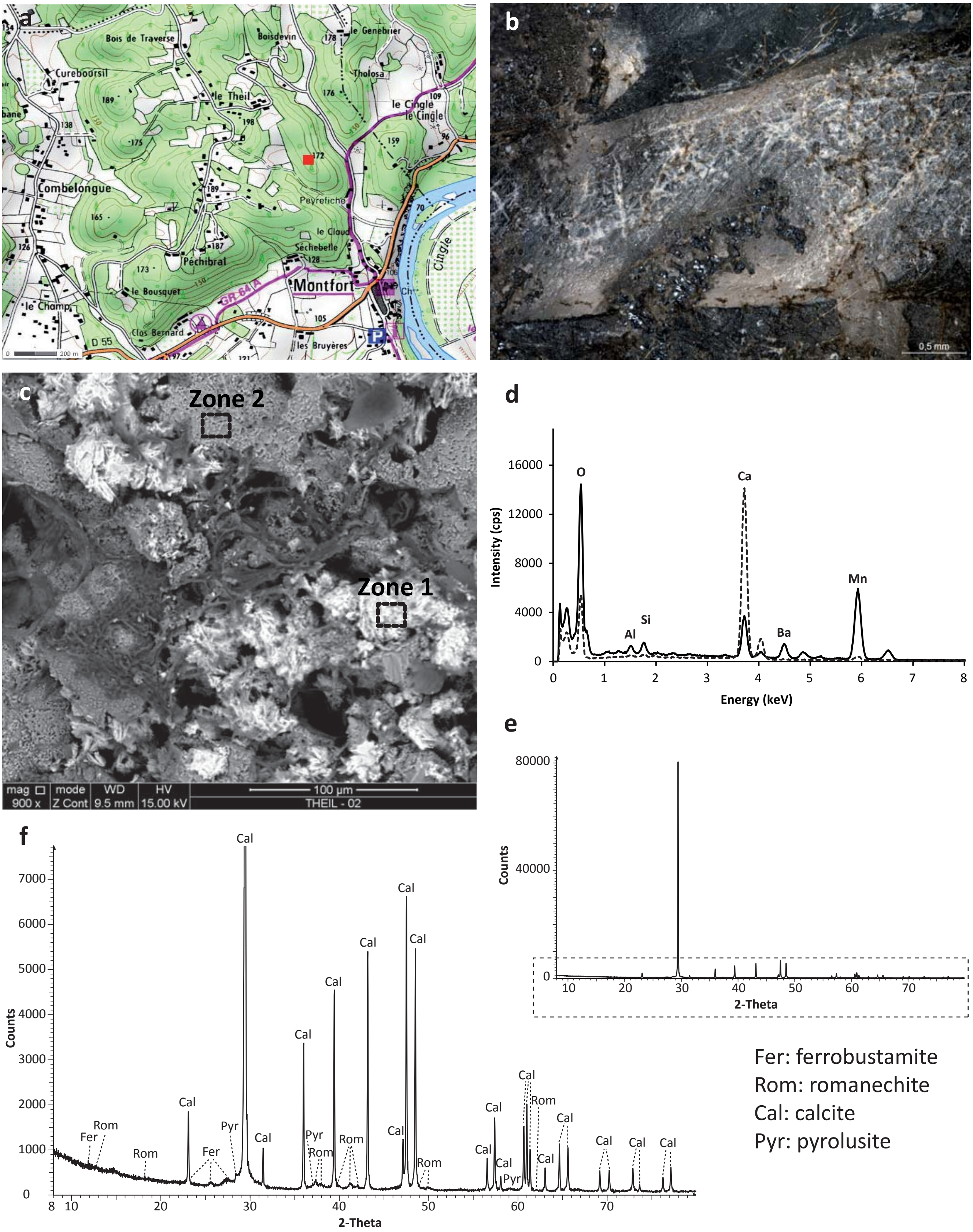

Figure G. Results of analyses conducted on samples from *Mine de Le Theil*. a: location of the outcrop (red square); b: macro photo of the surface of the sample THE-02; c: SEM image in BSE mode of the sample (squares indicate analysed areas); d: EDS spectrum of zones 1 and 2 (straight and dotted line, respectively) indicated in c; e: X-ray diffractogram obtained from pulverized sample; f: zoom of the X-ray diffractogram represented in e.

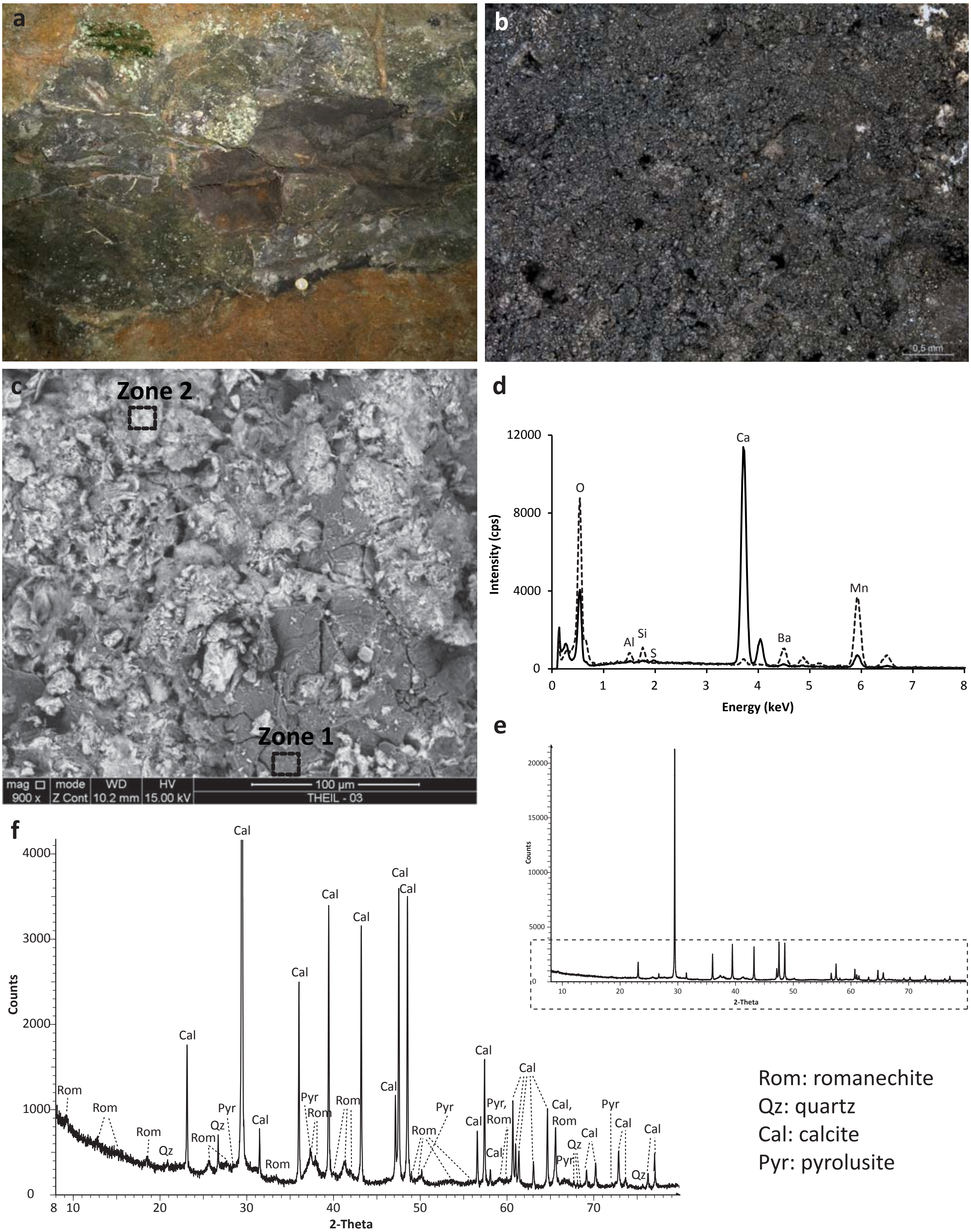

Figure H. Results of analyses conducted on samples from *Mine de Le Theil*. a: photo of the sampled area; b: macro photo of the surface of the sample THE-03; c: SEM image in BSE mode of the sample (squares indicate analysed areas); d: EDS spectrum of zones 1 and 2 (straight and dotted line, respectively) indicated in c; e: X-ray diffractogram obtained from pulverized sample; f: zoom of the X-ray diffractogram represented in e.

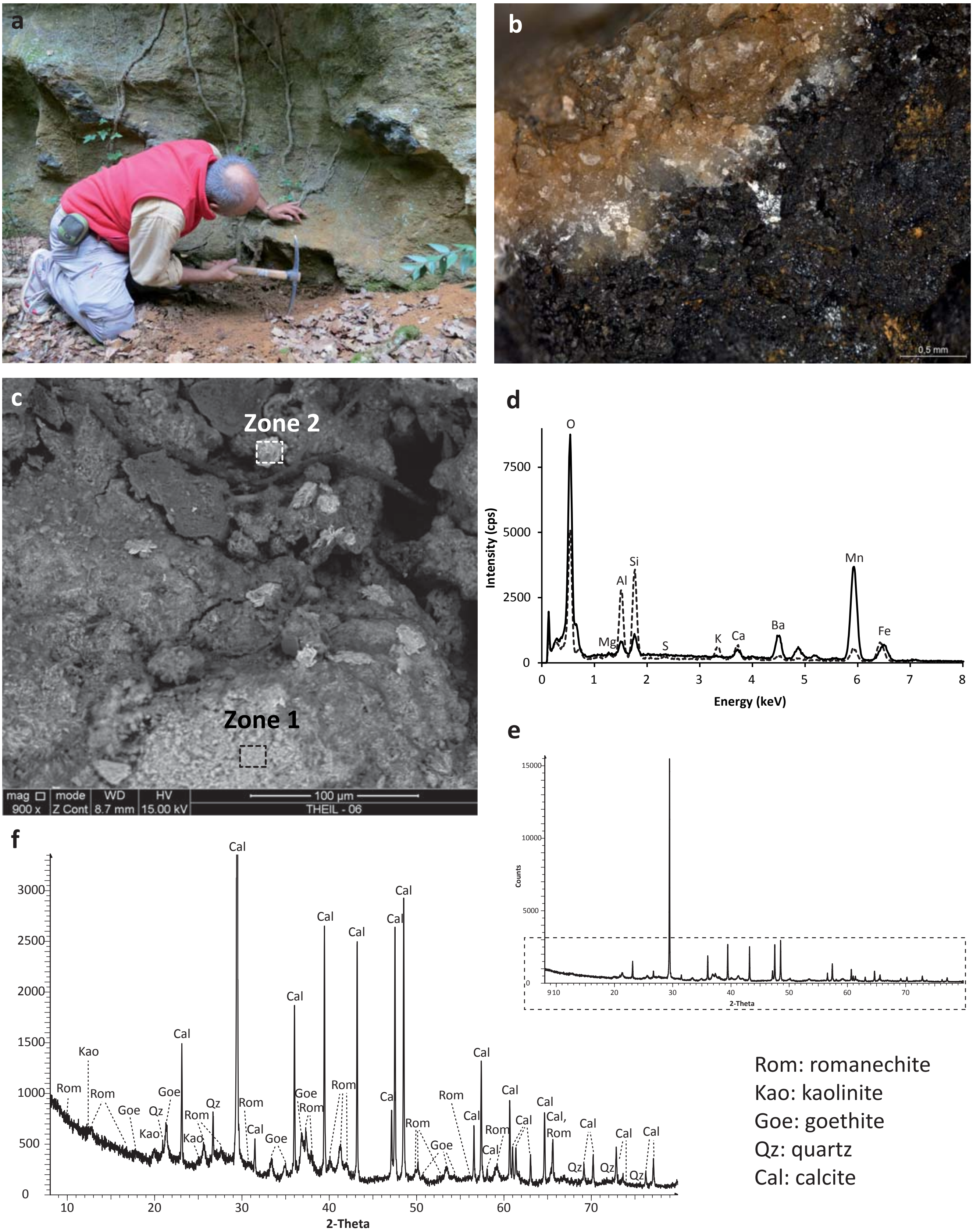

Figure I. Results of analyses conducted on samples from *Mine de Le Theil*. a: photo of the sampled area; b: macro photo of the surface of the sample THE-06; c: SEM image in BSE mode of the sample (squares indicate analysed areas); d: EDS spectrum of zones 1 and 2 (straight and dotted line, respectively) indicated in c; e: X-ray diffractogram obtained from pulverized sample; f: zoom of the X-ray diffractogram represented in e.

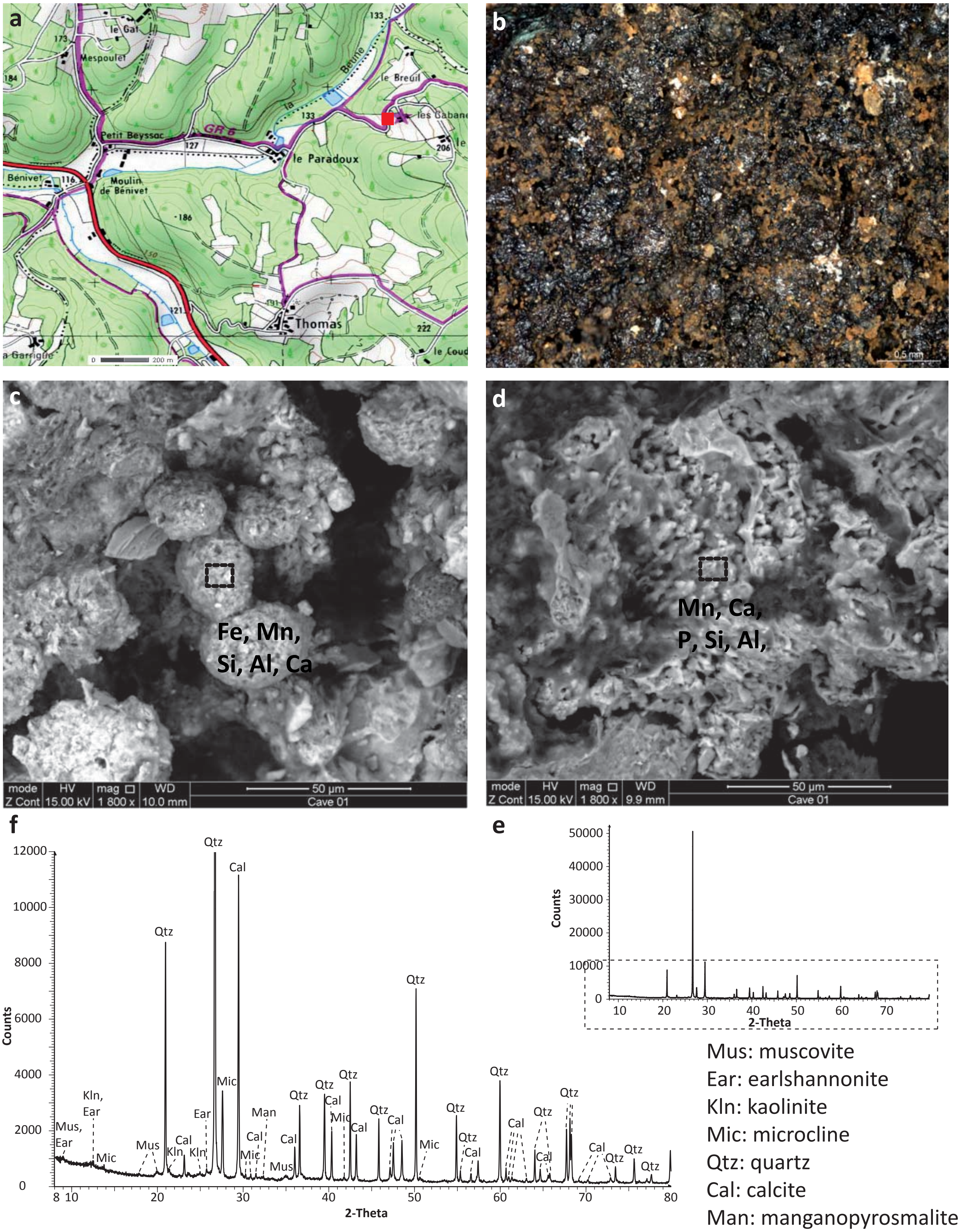

Figure J. Results of analyses conducted on samples from *Grotte-Cave*. a: location of the cave (red square); b: macro photo of the surface of the sample CAV-01; c, d: SEM images in BSE mode of the sample (squares indicate analysed areas); e: X-ray diffractogram obtained from pulverized sample; f: zoom of the X-ray diffractogram represented in e.

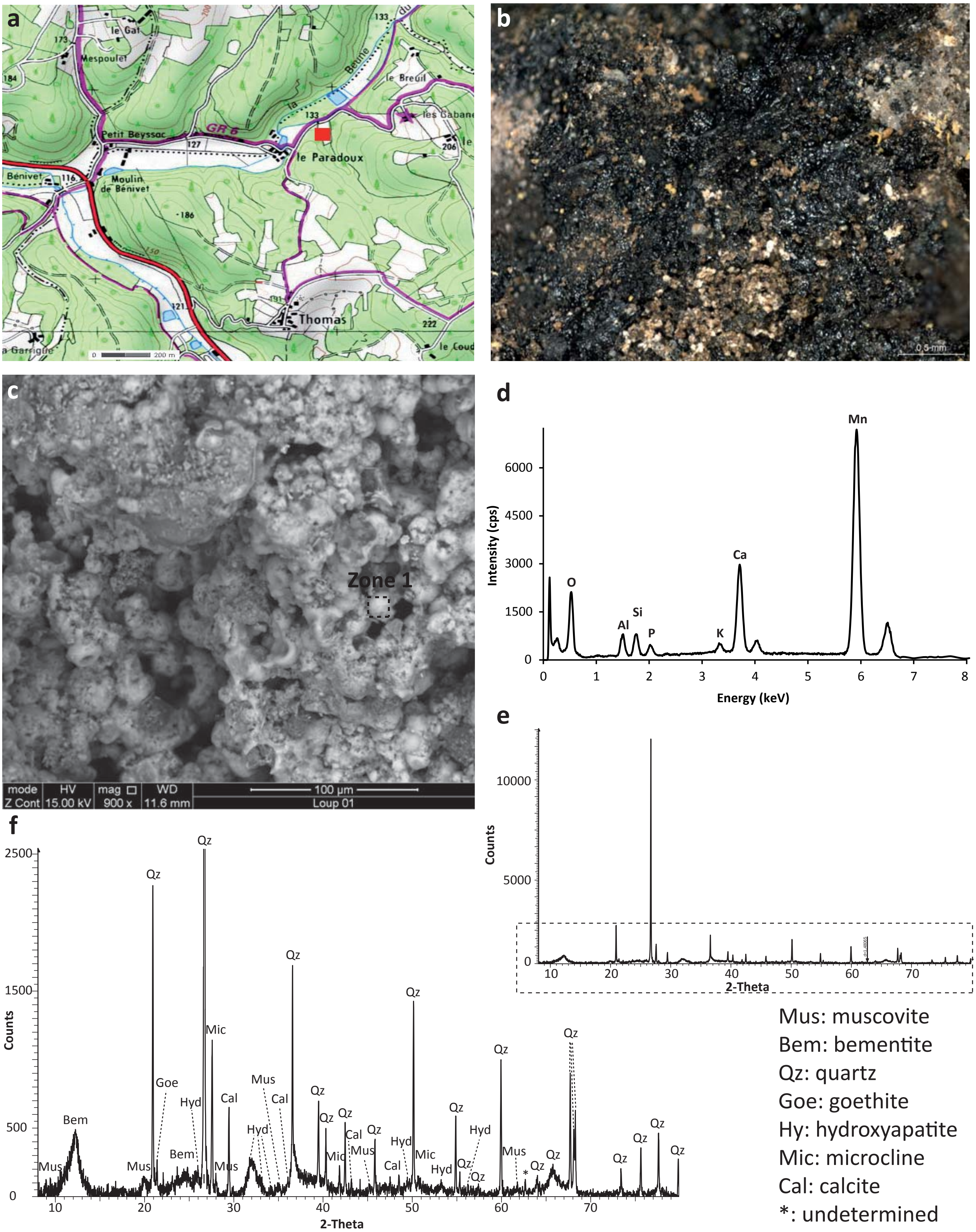

Figure K. Results of analyses conducted in samples from *Grotte du Loup*. a: location of the cave (red square); b: macro photo of the surface of the sample LOU-01; c: SEM images in BSE mode of the sample (square indicates analysed area); d: EDS spectrum of zone 1 represented in c; e: X-ray diffractogram obtained from pulverized sample; f: zoom of the X-ray diffractogram represented in e.

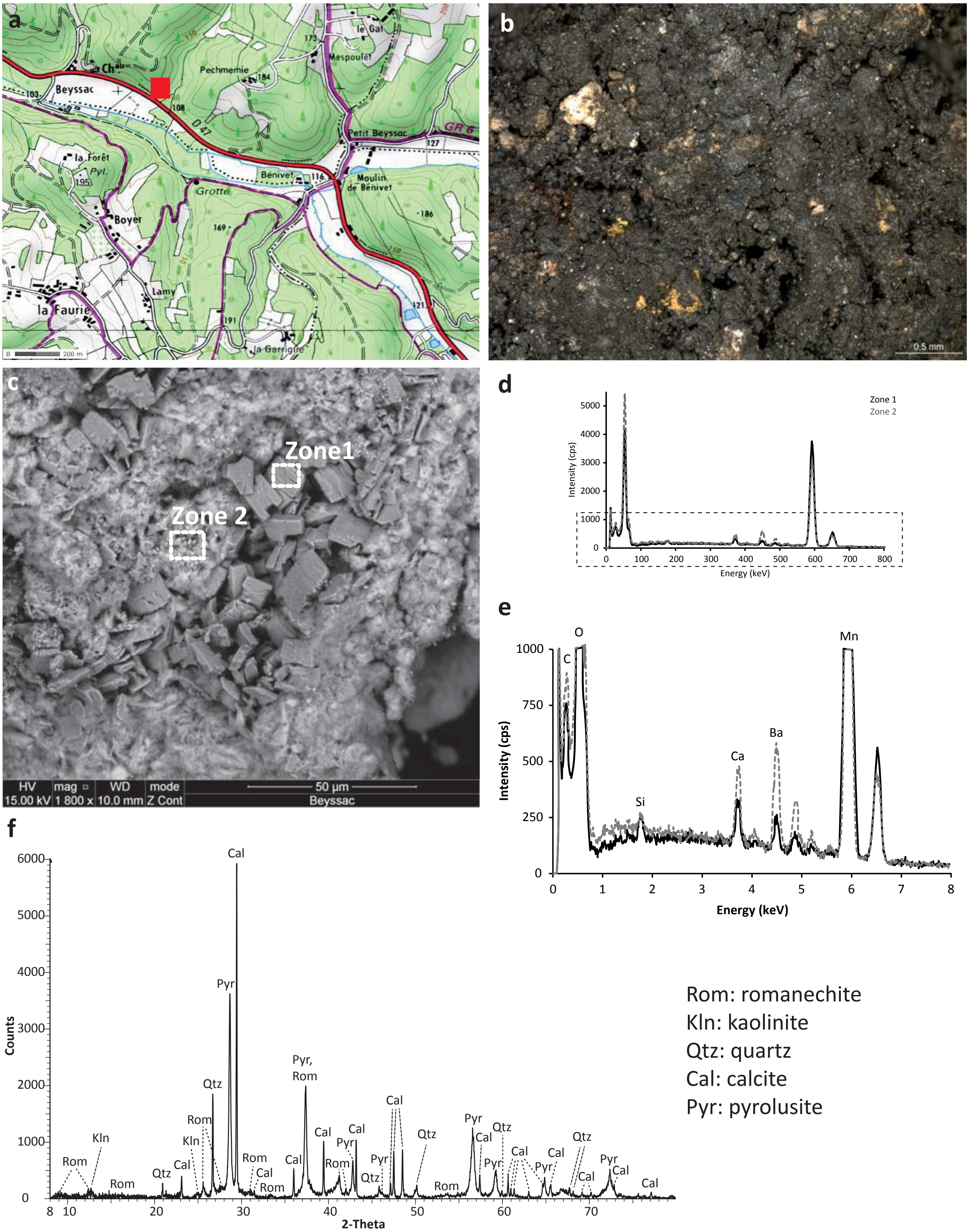

Figure L. Results of analyses conducted in samples from *Grotte de Beyssac*. a: location of the cave (red square); b: macro photo of the surface of the sample BEY-01; c: SEM image in BSE mode of the sample (zones 1 and 2 indicate analysed areas); d, e: EDS spectra at different scales of zones 1 and 2 indicated in c (straight and dotted line, respectively); f: X-ray diffractogram obtained from pulverized sample.

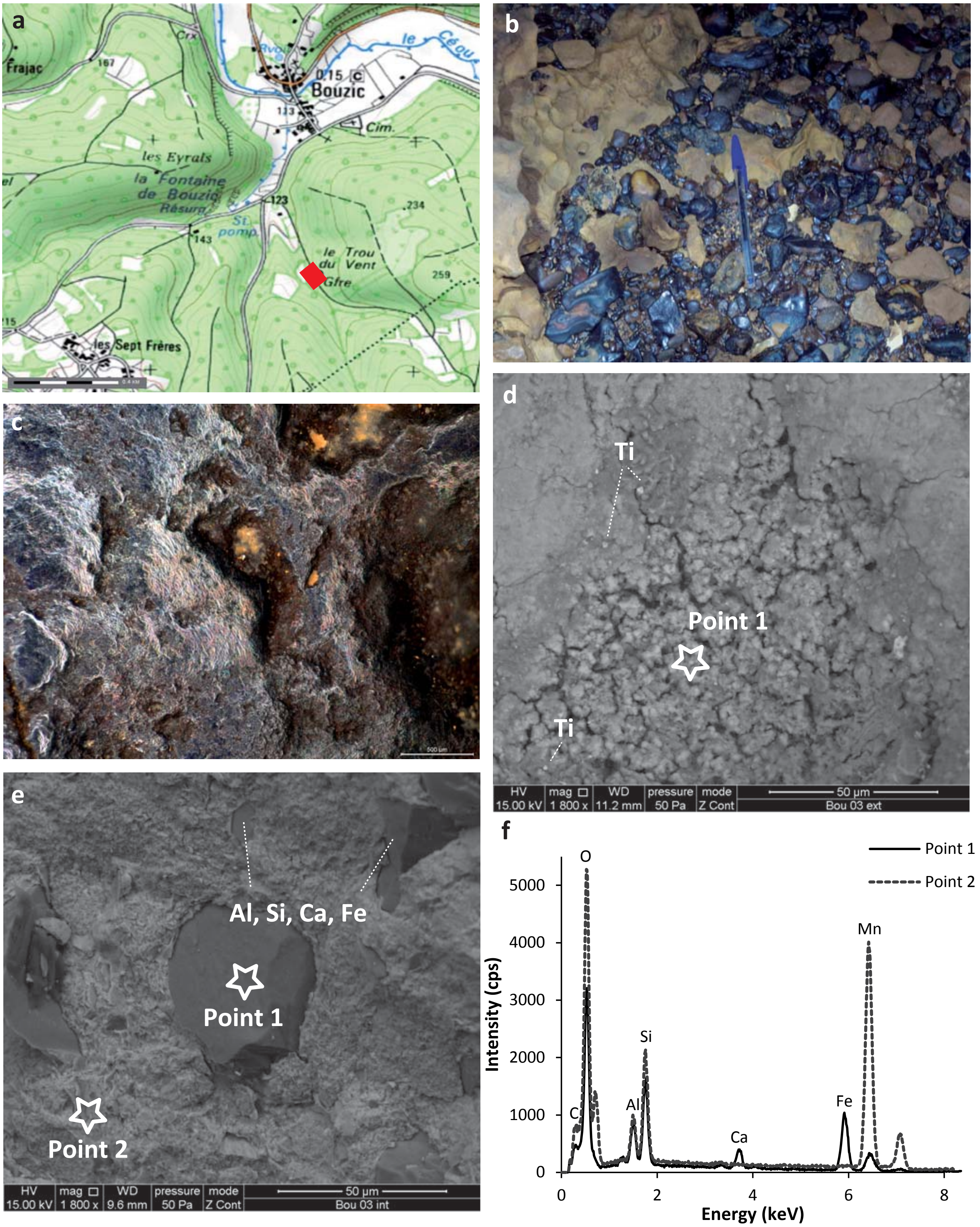

Figure M. Results from analyses conducted on samples from *Grotte du Trou du Vent - Bouzic*. a: location of the cave (in red); b: photo of the sampled area (@O. Huard); c: macro photo of the sample BOU-03 ; d, e: SEM images in BSE mode of the sample (d: external part; e: internal part). Stars indicate analysed spots; f: EDS spectra of spots 1 and 2 indicated in d and e.

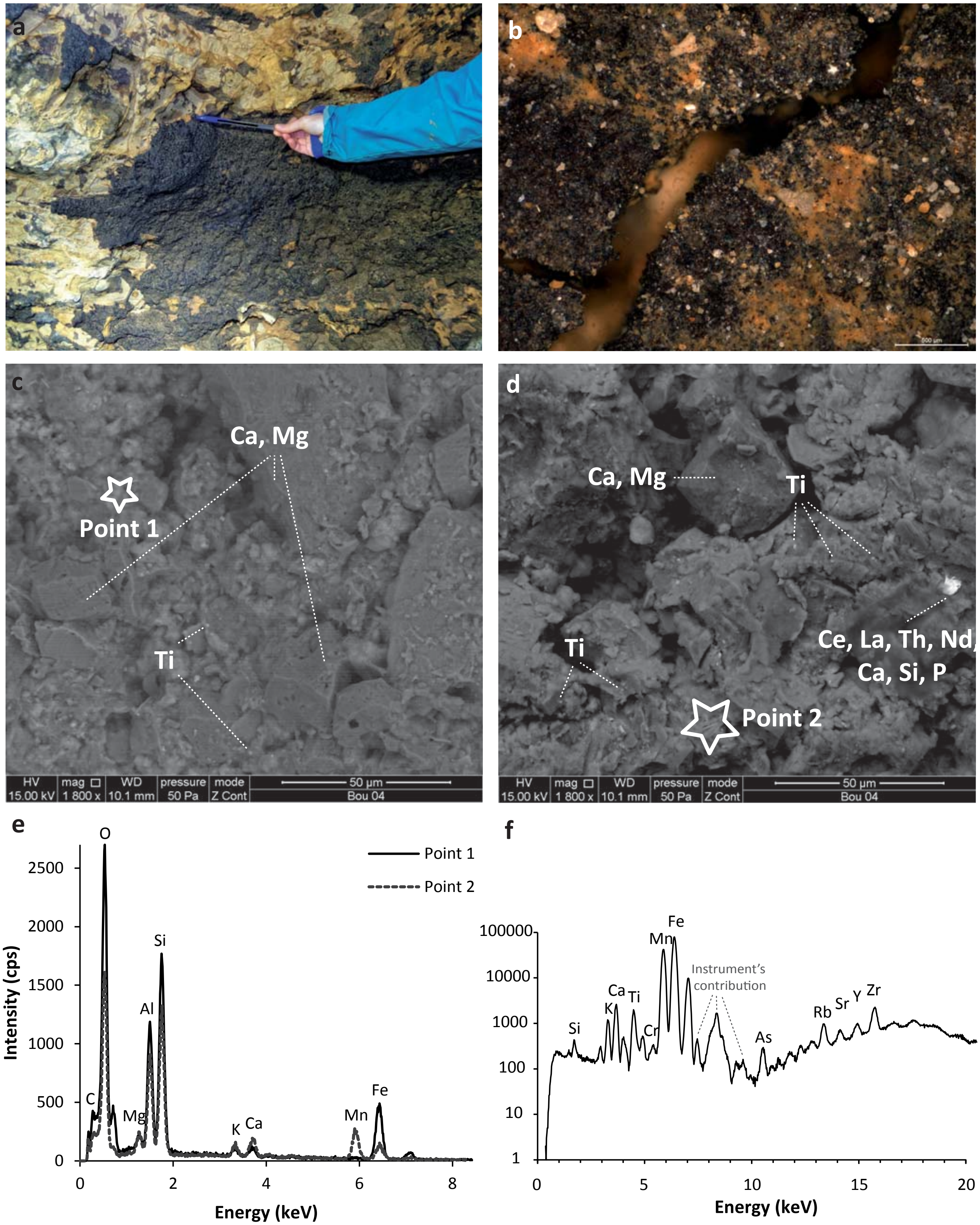

Figure N. Results from analyses conducted on samples from *Grotte du Trou du Vent - Bouzic*. a: photo of the sampled area (@O. Huard); b: macro photo of the sample BOU-04; c, d: SEM images in BSE mode of the sample. Stars indicate analysed spots; e: EDS spectra of spots 1 and 2 indicated in c and d; f: EDXRF sprectum from pulverized sample.

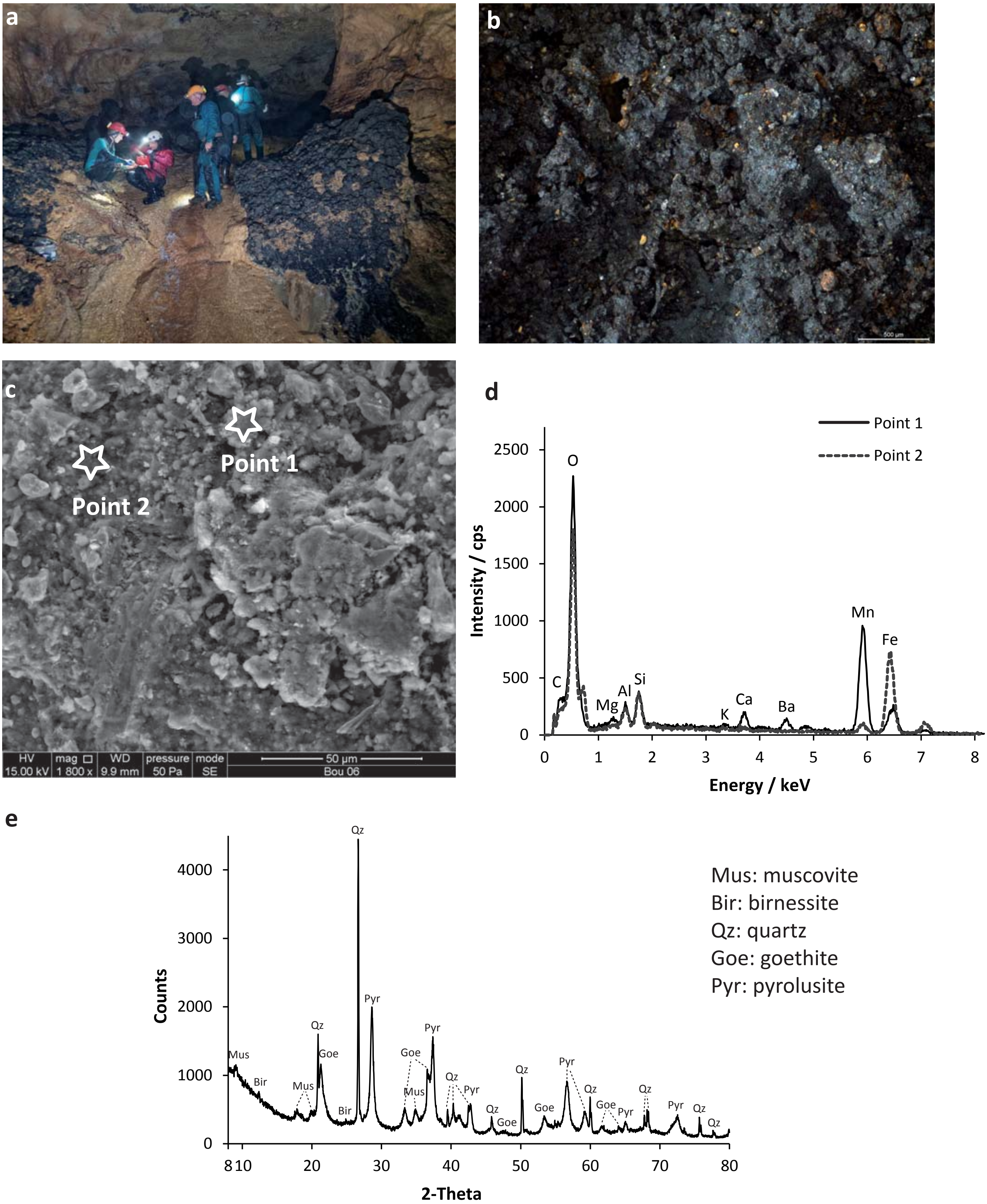

Figure O. Results from analyses conducted on samples from *Grotte du Trou du Vent - Bouzic*. a: photo of the sampled area (@O. Huard); b: macro photo of the sample BOU-06; c: SEM image in SE mode of the sample. Stars indicate analysed spots; e: EDS spectra of spots 1 and 2 indicated in c; e: XRD sprectum from pulverized sample.

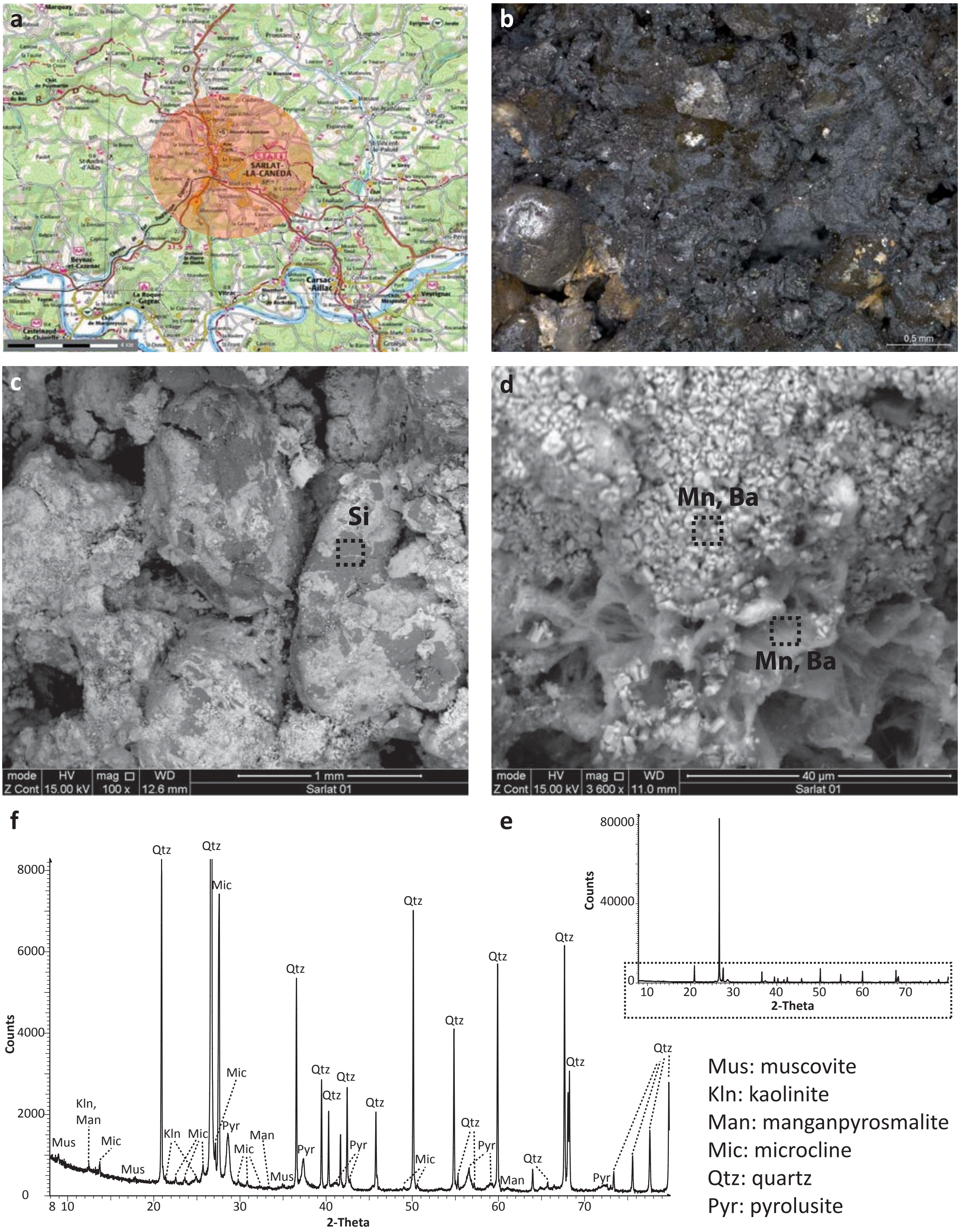

Figure P. Results from analyses conducted on samples from *Sarlat*. a: approximate location of the outcrop; b: macro photo of the surface of the sample SAR-01; c, d: SEM images in BSE mode of the sample (zones indicate analysed area); e, f: X-ray diffractogram obtained from pulverized sample.

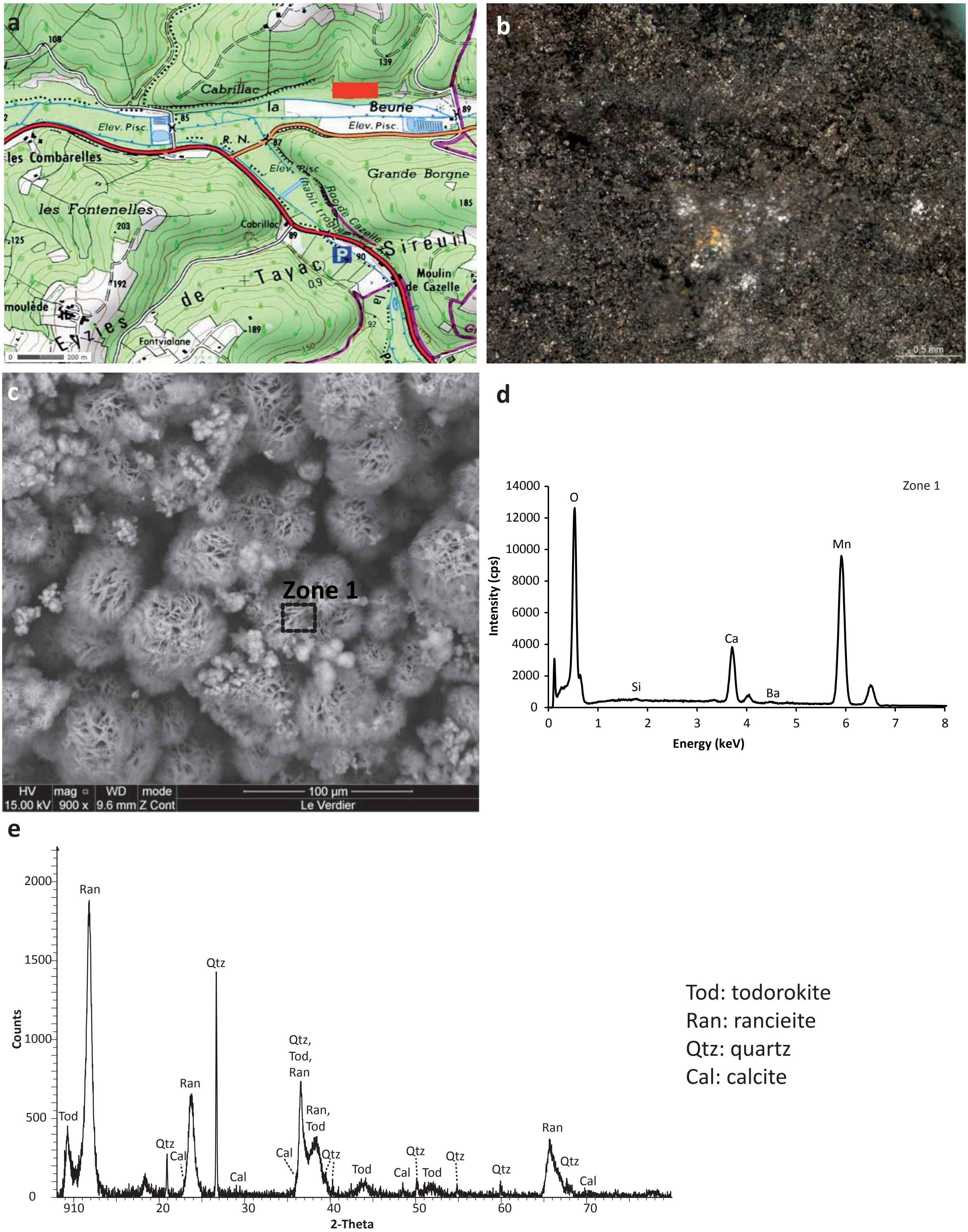

Figure Q. Results of analyses conducted on samples from *Carrière Le Verdier*. a: location of the outcrop (red square); b: macro photo of the surface of the sample VER-01; c: SEM image in BSE mode of the sample (zone 1 indicate analysed area); d: EDS spectrum of zone 1 indicated in c; e: X-ray diffractogram obtained from pulverized sample.

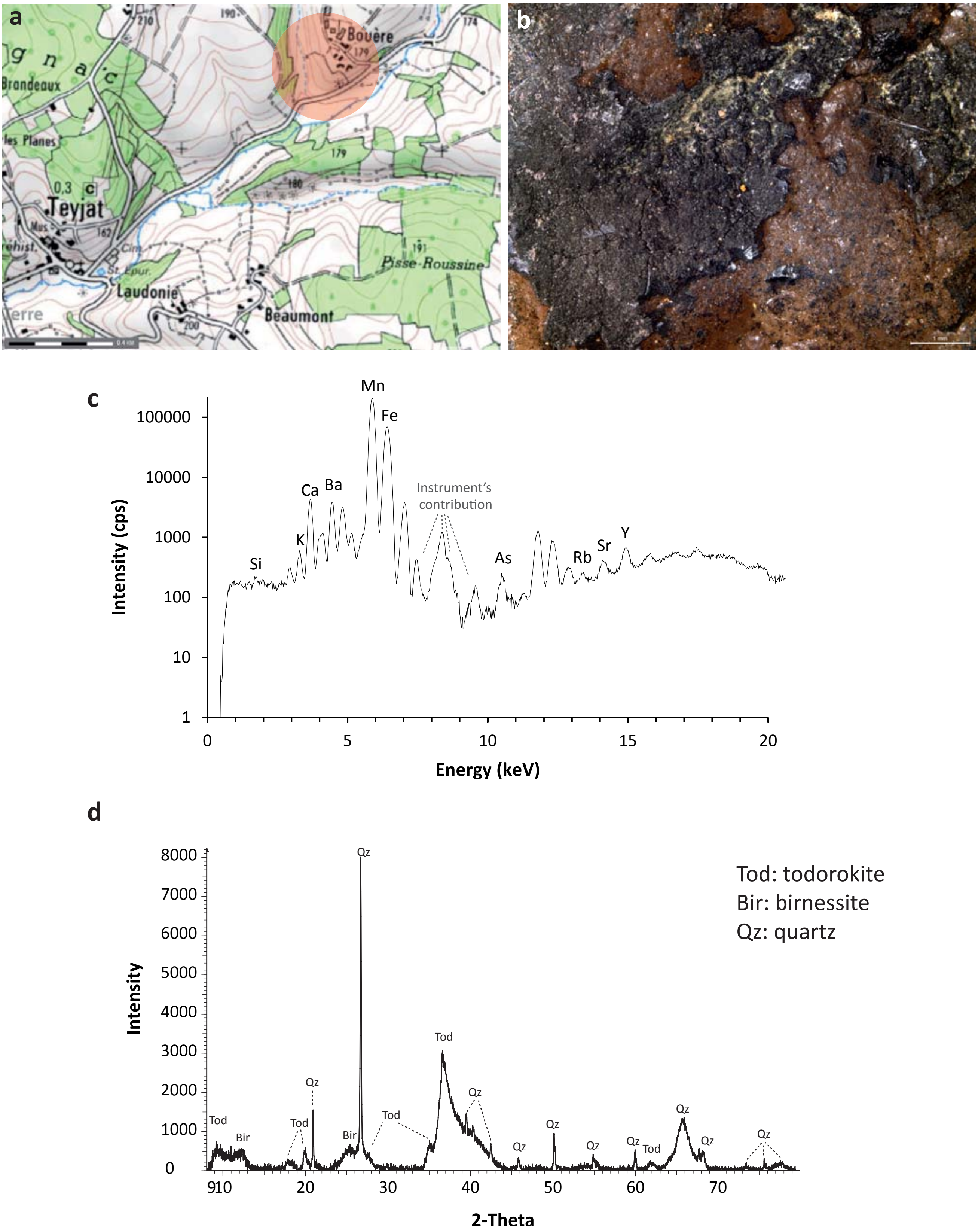

Figure R. Results of analyses conducted on the sample from *Teyjat*. a: location of the outcrop (in red); b: macro photo of the sample; c, d: EDXRF and XRD sprecta from pulverized sample.

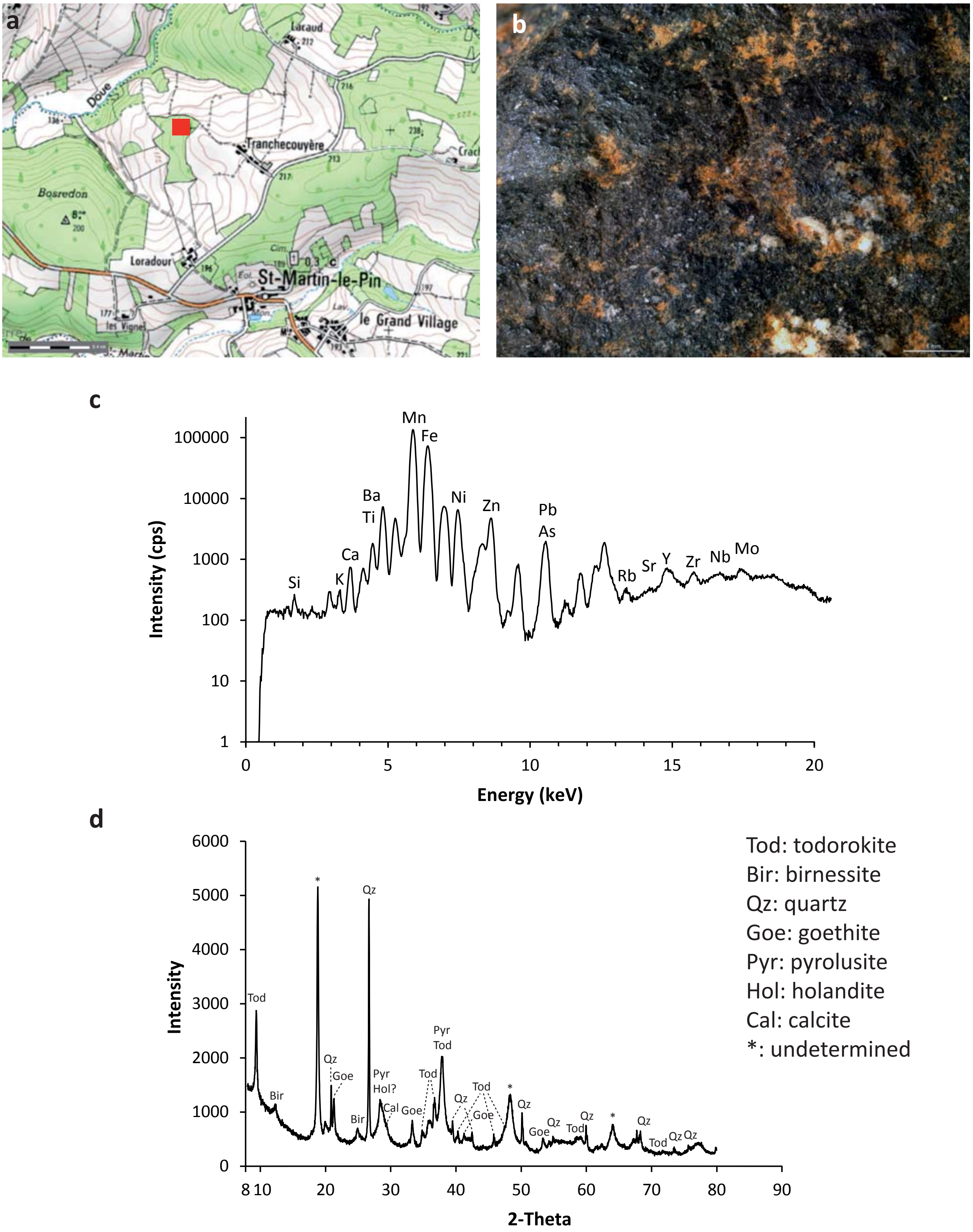

Figure S. Results of analyses conducted on the sample from *Tranchecouyère*. a: location of the outcrop (in red); b: macro photo of the sample; c, d: EDXRF and XRD sprecta from pulverized sample.
