# Supplementary material for: Provenance, modification and use of manganese-rich rocks at Le Moustier (Dordogne, France)
Source: PLoS One. 2019 Jul 17;14(7):e0218568. doi: 10.1371/journal.pone.0218568 (PMC6636720; doi:10.1371/journal.pone.0218568)
Supplement: S1 Text — (PDF) [file pone.0218568.s005.pdf]

Provenance, modification and use of manganese-rich rocks at Le Moustier, Dordogne, France

Africa Pitarch Martí*, Francesco d'Errico, Alain Turq, Eric Lebraud, Emmanuel Discamps, Brad Gravina.

*Corresponding Author

E-mail: africa.pitarch@gmail.com (APM)

S1 Text

Data on Mn-rich samples from geological outcrops including information on provenance, geological context, textural, elemental and mineralogical composition

Mine d'Albas

The *Albas* mine, located in the commune of Albas, known locally as the "tunnel de la manganèse", is a Mn-bearing Jurassic limestone (Astruc, 1986). Excavated at the beginning of the twentieth century to test its potential as a commercial source of Mn, it was, apparently, never exploited. Three samples from different locations within this 30 m long tunnel were collected. Samples ALB-01 and -02 come from the very beginning of the tunnel, on the right side of the entrance. Sample ALB-03 was taken at the end of the tunnel, on its left side. ALB-01 and -02 come from millimetric-thick layers of Mn-deposits sandwiched in the yellow limestone. Under optical microscope translucent yellow sparite crystals are surrounded by tiny black particles. SEM-EDS analysis shows a rather heterogeneous material composed of regular (Ca; c. 10 µm in length) covered by smaller acicular crystals (Mn, Ba) randomly distributed in the sample. XRD analyses detects the presence of simple and complex Mn-rich oxi-hydroxides (pyrolusite, birnessite, romanèchite), carbonates (calcite), iron oxi-hydroxides (goethite), micas (muscovite), and silicates (quartz). Sample ALB-03 is a black limestone with a fine and very uniform grain size. SEM-EDS analysis show the presence of large blocky crystals with smooth surfaces (Ca; c. 40 µm in length) and inter/intragranular regions filled by small needle-like crystals (Mn, Ba). This last specimen is similar to samples from *Causse du Cluzel* and *Sals*.

Grotte de Beyssac

The *Beyssac* cave or *Grotte des douze entrées*, located just below the eponymous castle-fort, in the commune of Les Eyzies de Tayac- Sireuil, is part of a karstic system formed in a yellow Cretaceous calcarenite formation (Capdevila and Rigaud, 1987). The manganese deposits, consisting of loose masses of an homogeneous black matter, are enclosed in the limestone. Under optical microscopy sample BEY-01 is composed of aggregates of micrometric irregular translucent crystals coated with submicrometric black particles. SEM-EDS analysis indicates that the sample is composed of medium size irregular grains of carbonates and silicates (Ca, Si; ≤50 µm in length) interspersed with submicrometric irregular, needle-like, and lath-like crystals (c. 5 x 15 µm) of Mn compounds (Mn, Ba). EDS analyses detect Si, Al, Na, Mg, and P, consistent with aluminosilicates and phosphates. XRD analyses confirm the presence of

carbonates (calcite), silicates (quartz), simple and complex Mn oxides (pyrolusite, romanechite) as well as clay minerals (possibly from the kaolinite group).

Mine de Causse du Cluzel

The Mn-rich samples from this outcrop, located in the commune of Pontcirq, close to the eponymous village, were collected from the surface close to the entrance (c. 1.5 – 2 m wide) of a small abandoned mine dug into the local bedrock - a Jurassic micritic limestone (Astruc, 1986). Under optical microscopy, all samples (CAU-01, 02, 03) appear as a fine-grained, dark-coloured limestone. SEM-EDS analyses indicate that sample CAU-01 is a heterogeneous material composed of medium size blocky grains interpreted as carbonates (Ca; ≤350 μm in length) and inter/intragranular regions filled with Ba-rich manganese compounds (Mn, Ba). Mn mineralization takes the form of fine fibrous felted conglomerates organized in tree-like or radiating patterns of c. 5 μm in length, and in in stacked platelets (c. 30-40 μm in length). EDS reveals the presence of Si, Al, Fe, P, K, Mg, and Na. XRD analyses confirm the presence carbonates (calcite), simple and complex manganese oxides (pyrolusite, romanechite, and hollandite), silicates (quartz), and iron hydroxides (goethite).

Grotte-Cave

The *Grotte-Cave*, located in the commune of Saint André d'Allas, is part of a karst system hosted in a yellow Cretaceous calcarenite (Capdevila and Rigaud, 1987). The manganese deposits take the form of >5 mm thick, hard, compact masses of heterogeneous matter coating the cave walls. Under optical microscopy these concretions appear as agglomerates of particles of different granulometry, texture and colour (with shades ranging from white to orange, and from brown to black). SEM-EDS reveals medium size tabular crystals (Si, K, Al) interpreted as feldspars, coated with Mn compounds comprising gel-like mineralization (Mn, Si, Al, Ca, Fe, K, Mg, Na, P), ~25 μm spherical agglomerates of submicrometric irregular crystals (Fe, Mn, Si, Ca, Al, P, K, Mg, Ti, Na, S) and amorphous masses (Mn, Ca, Si, P, Al, P, K, S, Na, Mg). The diffractograms identify carbonates (calcite), clay minerals (kaolinite group), micas (muscovite), silicates (quartz and K-rich feldspars, probably microcline), and some Mn compounds (a Mn phosphate – earlshannonite - and a Mn silicate - manganopyrosmalite).

Grotte du Loup

The *Loup* cave, located next to *Grotte-Cave*, belongs to the same karst system (Capdevila and Rigaud, 1987). The manganese concretions are found on the cave walls. They have a thickness of <4 mm and appear as fragile, compact masses of heterogeneous matter. At microscopic scale the samples (LOU-01, 02, 03, 04) are composed of agglomerates of micrometric to millimetric irregular translucent grains coated with dark brown to black particles and white to beige residues. SEM analysis of sample LOU-01 identifies the presence of poorly crystallized mineralizations taking the form of botryoidal agglomerates and amorphous masses with cracks. EDS detects Mn as the major element; Ca, Al, Si, Zn, K, Na, P, Mg, and S are also present. XRD detects carbonates (calcite), iron oxi-hydroxides (goethite), micas (muscovite), phosphates (hydroxyapatite), and different types of silicates (quartz, K-rich feldspars -probably microcline-, and Mn-rich silicates (possibly bementite). On samples LOU-03 and -04 a complex Mn oxi-hydroxide (birnessite) was detected.

Mine de Sals

At this outcrop, located in the commune of Labastide-du-Vert, thin bodies of Mn-rich material are enclosed in a limestone dated to the Jurassic (Astruc, 1986). According to informants from the closeby village, small trenches were dug at the beginning of the twentieth century to test the potential of the Mn ore deposit. Under optical microscopy, the samples (SALS-01, 02, 03) appear as a dense, fine grain black limestone. Under SEM-EDS samples SALS-01 and SALS-02 show 350 µm long blocky grains with smooth surfaces interpreted as carbonates (Ca) and inter/intragranular regions occupied by Mn compounds (Mn, Ba, K). These compounds take the form of fine fibrous felted conglomerates showing various crystal arrangements (dentritic and radiating) of c. 5 µm in length). Si, Al, Mg, P, and S point to the presence of clay minerals, phosphates, and sulphates. XRD confirm the presence of carbonates (calcite), clay minerals (probably from the kaolinite group), simple/complex Mn oxides (pyrolusite, hollandite) and phosphates (earlshannonite). It also detect different types of silicates (quartz and ferrobustamite).

Sarlat

No precise information is available about this Mn-rich ore deposit located in the commune of Les Eyzies-de-Tayac Sireuil. Samples were collected long ago, during the excavation of a large foundation trench. Apparently, this Mn-rich clayey sand deposit was filling dissolution cavities within Cretacic calcarenites. Under optical microscopy SAR-01 appears as a coarse grained sandstone impregnated with black manganese oxi-hydroxides showing a good degree of cohesion between particles. Yellow to orange deposits are present between the grains. SEM-EDS analysis indicates that this material is composed of coarse rounded grains of silicates (Si; ≤1 mm in length) coated with Mn compounds taking the form of fine equiaxed platy-like particles (Mn, Si, Ba, Ca; ~1 µm in length) and needle-like crystals (~15 µm in length) forming structures radiating from a central point. Al, K, and P were detected. The presence of silicates (quartz and feldspars), simple (pyrolusite) and more complex Mn oxides (manganopyrosmalite) is confirmed by XRD analyses. Clay minerals (kaolinite group) and micas (possibly muscovite) were also detected. Ba-containing minerals are not detected in the diffractograms, but this is no proof of their absence in the sample.

Mine de Le Theil

The *Le Theil* is a depleted manganese ore mine located in the Beunes valley, near Montfort. According to Chalmin (2003), it is a surface manganese orebody formed in a karstic environment. The Mn deposits are at the surface of a yellowish Cretaceous calcarenite (Capdevila and Rigaud, 1987). Several samples were collected as they appeared heterogeneous in the field. Their analysis reveals the following features:

#THE-02: coarsely translucent Mn-bearing calcite with scalenohedron-rhombohedron habit. Manganese oxi-hydroxides take the form of dendrites. SEM-EDS identifies large irregular grains of carbonate (Ca; skeletal shape) and inter/intragranular regions occupied by agglomerates of platelets (Mn, Ba; ~10 µm in length). The Ca-rich crystals are partially replaced by Mn oxi-hydroxides. Such replacement apparently moves gradually from the periphery toward the centre of the grain. Silicates, clay minerals, iron oxi-hydroxides, phosphates, sulphates, and chlorides are probably also present since EDS also identifies Si, Al, Fe, K, Na, Mg, S, P, and Cl.

#THE-03: soft, porous Mn-rich deposits are contained in a clayey matrix. Under optical microscopy, the sample appears composed of aggregates of fine-grained dark brownish grey powder. A few translucent grains are also identified. SEM-EDS also identifies large irregular grains of carbonates (Ca) and inter/intragranular regions occupied by agglomerates of stacked platelets and poorly crystallized mineralizations (Mn, Ba; ~10 in length). Contrary to #THE-02 Ca-rich crystals are almost entirely replaced by Mn oxi-hydroxides. EDS also reveals the presence of Si, Al, Cl, Mg and P.

#THE-06: Mn-rich hard concretions embedded in a calcarenite. Microscopically, the sample appears as a compact and hardened heterogeneous crust composed of fine-grained brownish black powder associated with a few small accumulations of fine-grained yellow to bright orange powder, as well as heterogeneous black grains. SEM-EDS shows that this sample is composed of medium size grains (0.5 – 1 mm in length) probably corresponding to carbonates and silicates covered with sub-micrometric platy-like particles interpreted as clay minerals, Mn compounds in the form of micrometric platy-like particles (Mn, Ba, Fe; 2 µm in length), and tiny flower-like agglomerates of platelets (Ba, Fe, Mn; ~15 µm in diameter) randomly distributed through the surface. EDS analyses also highlight the presence of Si, Al, K, Mg, Na, and S.

XRD analyses of the three samples detected the presence of carbonates (calcite), silicates (quartz), and both simple and complex Mn oxides (pyrolusite, romanechite), which is consistent with the results obtained with SEM-EDS. Clay minerals (probably from the kaolinite group), iron oxi-hydroxides (goethite), and a complex Ca-Fe-Mn-rich silicate (ferrobustamite) are also identified.

Teyjat

We were not able to collect samples from this mine, located between Beaumont, La Ronde, Chauffour and Boisseuil. Extensively exploited in the 19th century, the mine was subsequently closed and manganese ore veins are now at a certain depth. However, we have identified a stream near the close village of Bouère in which all the sediments, composed of pebbles, cobbles and boulders, were black in colour. This probably indicates that waters pass through the Teyjat Mn mineralization and enriching themselves in this element. Under optical microscopy, the sample collected in the stream (TEY-01), consisting of a large pebble, is coated with a thin black metallic patina. XRD analysis of this patina highlights the presence of complex oxi-hydroxides such as birnessite and todorokite, and silicates (quartz).

Tranchecouyère

Tranchecouyère is a small village located northwest of Saint Martin Le Pin. Small black nodules can be found in the vicinity of this locality, within the Tertiary clayish sediment. Under optical microscopy sample TRA-01 reveal a c. 4 mm thick, compact, hard black coating displaying a metallic sheen. The core is composed of limestone. XRD of the external coating identifies complex oxi-hydroxides (birnessite, hollandite, todorokite), carbonates (calcite), iron oxi-hydroxides (goethite), and silicates (quartz).

Grotte du Trou du Vent

The *Trou du Vent* cave, in Bouzic, is part of a karstic system hosted in Jurassic limestones (Astruc, 1990). The cave is closed to the public. We obtained a special permit to survey it. The

cave comprises 12 km galleries in which five types of Mn-rich deposits were identified: 1) nodules with a mat appearance (BOU-01); 2) patinas (BOU-02); 3) nodules presenting a metallic sheen (BOU-03, -05); 4) clayey deposits (BOU-04); 5) thick crusts covering the cave walls (BOU-06, -07). When analysed by EDXRF, #BOU-02 turned to be mostly composed of Fe and was excluded for this study.

#BOU-01: This dark brown hard, compact nodule has a diameter of c. 5 cm. Microscopically, it appears as a rather heterogeneous material composed of agglomerates of greyish-black particles associated with small accumulations of orange to brown particles and coarse irregular translucent crystals.

#BOU-03, 05: Dark greyish-brown in colour, these nodules were collected in different locations, including the "chaos" and the "planche à clous". They systematically appeared in the form of accumulations on the cave floor. Under optical microscopy these samples are composed of an external layer presenting a metallic sheen and a core comprising white and grey crystals embedded in a brownish matrix. SEM observations and EDS analyses identify a thin compact layer composed of agglomerated Mn-rich sub-circular crystals and a core composed of Ca-rich regular and irregular crystals embedded in a Fe/Si/Al-rich matrix of stacked platelets.

#BOU-04: Brownish black in colour, this clayey deposit was taken from the cave wall in a location known as "chaos". Under the microscope the sample appears to be composed of fine grained black particles associated with fine-grained yellow to bright orange particles, as well as medium translucent grains. Observations and analysis with SEM-EDS identify an admixture of medium sized platelets (Fe, Mn, Si, Al; c. 5 µm in length) probably corresponding to aluminosilicates, tiny irregular crystals (Ti; <1 µm in length) interpreted as Ti-rich minerals, and coarse irregular crystals (Ca, Mg; 40 µm) possibly carbonates. Rare earth minerals and carbon particles were also detected.

#BOU-06, 07: These botryoidal brownish-black crusts, found near a cascade, were covering the cave floor and, at places, the cave walls. They are one to two-centimeter thick. Microscopically, they appear composed of agglomerates of greyish-black particles associated with small accumulations of orange fine-grained particles, and coarse irregular translucent crystals. SEM-EDS analyses show a rather heterogeneous material composed of an admixture of complex Ba-rich manganese compounds, taking the form of agglomerates of acicular sub-micrometric, regular micrometric, and stacked micrometric platelets, and some kind of iron-rich aluminosilicate appearing as amorphous masses or agglomerates of stacked micrometric platy-like particles. Phosphates were also detected. XRD analysis of BOU-06 confirms the presence of complex Mn oxi-hydroxides (birnessite) and simple Mn oxides such as pyrolusite, as well as carbonates (calcite), iron oxi-hydroxides (goethite), micas (muscovite), and silicates (quartz).

Carrière Le Verdier

This outcrop is an abandoned gravel quarry located in the commune of Les Eyzies de Tayac-Sireuil. It consists of loose deposits of rock debris accumulated at the base of a slope (Karnay, 1999), which include nodules of Mn-rich material. The nodule sampled is dark brown, almost black in colour. Under optical microscopy the nodule is composed of particles with a low degree of cohesion. SEM shows a fairly homogeneous matter composed of agglomerates of tiny spheres (1 to 60 µm in diameter) presenting a spongy-like structure. EDS identifies Mn,

Ca, Ba, K, and Si. XRD analyses detects complex hydrous manganese oxide minerals (rancieite and todorokite), carbonates (calcite) and silicates (quartz).

References

ASTRUC J-G. Carte géol. France (1/50 000), feuille Puy-L'Evêque (856), Orléans : BRGM. Notice explicative par J.-G. Astruc, J. Galharague, Ch. Vautrelle., J.-C. Soule (1986) 38 p.

ASTRUC J-G. Carte géol. France (1/50 000), feuille Gourdon (832), Orléans : BRGM. Notice explicative par J.-G. Astruc (1990) 45 p.

CAPDEVILA J-P, RIGAUD, J-P. Carte géol. France (1/50 000), feuille Sarlat-la-Canéda (808), Orléans : BRGM. Notice explicative par J.-P. Capdevila, J.-P. Rigaud, D. Fauconnier, C. Azema, Ch. Monciardini (1987) 28p.

CHALMIN E. Caractérisation des Oxydes de Manganèse et Usage des Pigments Noirs au Paléolithique Supérieur. Thesis, Université de Marne-La-Vallée, Paris, France, 2003.

KARNAY G. Carte géol. France (1/50 000), feuille Le Bugue (807), Orléans : BRGM. Notice explicative par G. Karnay, N. Aujoulat, S Konik, B. Mauroux, E. Pluchery, A. Turq (1999) 86 p.
